# Empirical methods that provide physical descriptions of dynamic cellular processes

**DOI:** 10.1016/j.bpj.2024.12.003

**Published:** 2024-12-04

**Authors:** Ian Seim, Stephan W. Grill

**Affiliations:** 1 Max Planck Institute of Molecular Cell Biology and Genetics, Dresden, Germany; 2 Center for Systems Biology Dresden (CSBD), Dresden, Germany; 3 Cluster of Excellence Physics of Life, TU Dresden, Dresden, Germany

## Abstract

We review empirical methods that can be used to provide physical descriptions of dynamic cellular processes during development and disease. Our focus will be nonspatial descriptions and the inference of underlying interaction networks including cell-state lineages, gene regulatory networks, and molecular interactions in living cells. Our overarching questions are: How much can we learn from just observing? To what degree is it possible to infer causal and/or precise mathematical relationships from observations? We restrict ourselves to data sets arising from only observations, or experiments in which minimal perturbations have taken place to facilitate observation of the systems as they naturally occur. We discuss analysis perspectives in order from those offering the least descriptive power but requiring the least assumptions such as statistical associations. We end with those that are most descriptive, but require stricter assumptions and more previous knowledge of the systems such as causal inference and dynamical systems approaches. We hope to provide and encourage the use of a wide array of options for quantitative cell biologists to learn as much as possible from their observations at all stages of understanding of their system of interest. Finally, we provide our own recipe of how to empirically determine quantitative relationships and growth laws from live-cell microscopy data, the resultant predictions of which can then be verified with perturbation experiments. We also include an extended supplement that describes further inference algorithms and theory for the interested reader.

## Significance

A major goal of cell and developmental biology is to provide quantitative, mechanistic descriptions of living cells and organisms. Perturbations such as gene knockouts have successfully been used to infer functions of molecules at the cell or organism scale. However, cells have evolved to be robust, and as a result, strong perturbations can give rise to compensation phenomena including restructuring of underlying molecular interaction networks. The interpretation of results is challenging in such cases. In this article, we review inference approaches that rely only on observational data of cells and organisms with interaction networks subject to minimal perturbations, ideally as they naturally occur. We present a range of perspectives and techniques, including statistical relationships, causal inference, and dynamical systems approaches.

## Introduction

### The success of biological perturbations and the need for observational studies

In the endeavor to understand molecular cell biology, scientists have leveraged perturbations of living cells to observe their effects and infer their underlying structures. Genetic perturbations are among the most widely used and successful examples of these approaches. The earliest studies used chemicals or radiation to randomly mutate DNA and screened resulting organisms based on phenotypes to infer gene functions. Perhaps the first example of such an approach came in 1927 with Hermann Muller who discovered that x rays mutate DNA in *Drosophila* (1,2). A major advance came with the advent of site-directed mutagenesis techniques in the 1970s, in which researchers could target specific genes (3,4,5). Since then there have been astounding advances in the ability to precisely and easily modify genomes of living organisms. Perhaps most notably, the understanding in 2012 and 2013 that the bacterial/archaeal viral defense system, clustered regularly interspaced short palindromic repeats (CRISPR), could be used broadly as a gene editing tool has transformed biological research (6,7). Another important genetic manipulation tool with historical ties to antiviral responses is RNA interference (RNAi) (8,9), first definitively demonstrated in *Caenorhabditis elegans* in 1998 (10). RNAi limits or eliminates gene expression via mRNA degradation and can therefore be used as a titration of the effects of a gene of interest (11,12,13). Altogether, gene knockouts, knockdowns, and specific mutations allow researchers to remove or alter genes and their expression to determine their effects on cell biology and development.

Although these tools and others have been important in uncovering the components of molecular pathways and associations with cellular physiology, we argue that observational approaches can be useful for obtaining a quantitative and complete understanding of cellular systems. In what follows, we distinguish between observational and perturbative studies. Observing a particular biological process often requires the use of transgenic reporters, and we here consider the addition of reporter molecules that only minimally impact the system’s dynamics as essential components of a nonperturbative observational study. However, experiments in which molecules have been knocked down, removed, or mutated (other than tagged) to change the underlying interaction networks will be considered perturbations.

Our focus on observational studies is motivated by several concepts. First, observational studies are simpler and can often provide a wealth of data. Second, knockdown and knockout experiments help to make hypotheses about the components of interaction networks and correlations among them, but in general they are not sufficient to describe the quantitative nature of these relationships. Third, perturbations always entail the risk of altering the interaction networks since cells can compensate with rewired or redundant networks (14,15,16). However, predictions from observational studies must ideally be checked with targeted perturbations in which the results can be understood within their natural context. Therefore, both approaches are necessary for unraveling the complex spatiotemporal processes that underlie living systems. We first provide an example of successful observational inference in the history of the discovery of gravity.

### The inference of gravity from observations

The perhaps most striking example of scientific inference using only observational data can be found in the history of the discovery of gravity. The story begins when, as a boy, Tycho Brahe saw a solar eclipse and decided to dedicate his life to astronomy (17). In 1571 he constructed his own observatory where he began a project that resulted in the most accurate measurements that had ever been collected of the positions of the Sun, the Moon, the Earth, and the five other known planets. Tycho also dabbled in theory and proposed a geocentric model of the universe due to his view that the Earth was too heavy to move much (18), which gave equivalent predictions about planetary positions as the heliocentric Copernican model upon a coordinate transformation (19). As neither theory provided a mechanistic explanation for the motions, neither could be ruled out (19).

In 1600, Tycho met Johannes Kepler in Prague, and Kepler became his assistant. During their one year working together before Tycho’s death, Tycho refused to share his observations with Kepler, who stole them afterward (17). With this world-class data, Kepler sought to improve upon Tycho’s model (20). As the geocentric view was popular at the time, Kepler made his money as a court astrologer and worked on his theories on his own time (17). Ultimately, Kepler used observations of the distance of planets from the Sun along with measurements of the heliocentric longitude to show that orbits were not circular (as stated in the models of both Tycho and Copernicus), but instead were ovals (21). He also noted that the speeds of the planets were inversely proportional to their distance from the Sun, a pivotal example of empirical data analysis. From this, he proposed the existence of an “invisible solar force” pushing planets along and dying off with distance (20). He also proposed a similar force emanating from the Earth, which affects the Moon (22). Combining these observations and inferences, he showed that the orbits in fact had to be elliptical, in addition to his other two laws.

Although Kepler’s model was not immediately widely adopted, astronomers came to realize that his framework gave the most accurate predictions of the planetary positions of any existing model (20). Seventy-five years later, Isaac Newton published his theory of universal gravitation, in which he derived Kepler’s laws as a consequence of the more fundamental gravitational force and unified celestial and earthly phenomena (21). In this step, Kepler’s hypothesized invisible solar force was revealed quantitatively, and classical mechanics and the Industrial Revolution soon followed. Then, 240 years later, Einstein proposed a yet deeper understanding of gravity in his theory of general relativity, in which gravity is a consequence of the curvature of space time (23). The theory was supported to a definite degree by careful observations of gravitational lensing of starlight, predicted by general relativity and visible during a solar eclipse in 1919 (24). The theory of general relativity has led to predictions well beyond the original scope envisioned by Einstein, including black holes and gravitational waves, which have since been experimentally observed (25).

Together, this almost 300-year-long story of discovery provides a beautiful example of careful analysis of precise observations yielding the very deepest of insights. Importantly, these insights came about without the necessity to perform a perturbation experiment by, for example, removing the sun to investigate the impact on the motions of planets.

### Towards a comprehensive understanding of molecular cell biology

Next, we will summarize how some of the perspectives and techniques from observational studies have been or may be applied to cell biology. We will focus on the goal of constructing quantitative descriptions of cellular and molecular interaction networks that ultimately give rise to cellular physiology and organismal development. This goal is lofty due to the complexity of cells and developmental processes, which are influenced by the large size of molecular interaction networks (26) and the near certainty of unobserved variables; the presence of noise, including intrinsic noise due to chemical reactions and external noise (27); redundancy in networks in which several molecular interaction networks can achieve the same function (12,14); and nonergodic dynamics in which the topology of interaction networks changes over time (28,29,30). Despite these challenges, we focus on techniques to gain understanding using an observational approach. Therefore, in this review we focus our thoughts on the following two questions: How much can we learn from just observing? What level of detail about relationships can we obtain from observations? We end with a suggested recipe for scientific inquiry in molecular cell biology using an example in *C. elegans* oocytes: learn as much as possible from observations, make precise predictions, and do targeted perturbations to either confirm or refute those predictions.

## Inference for dynamic biological systems

**Figure 1:**
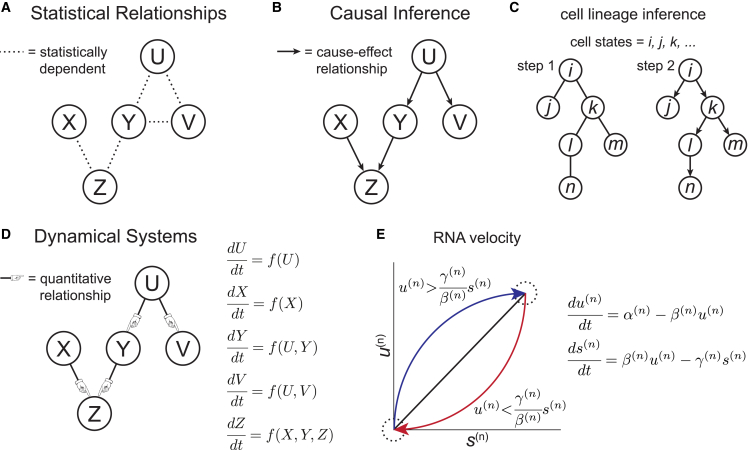
Hierarchy of descriptions of interaction networks. (*A*) Statistical dependence between variables is indicated with dashed lines. Variables with a common cause (*Y* and *V*) are statistically dependent. (*B*) Arrows indicate directional causal interactions among variables forming a directed acyclic graph (DAG). (*C*) An example of causal inference in cell lineage inference is shown. In step 1, an undirected network is constructed, and in step 2, the edges are directed to indicate ancestor-descendant relationships. (*D*) Variables and their dynamics are represented as a DAG with an accompanying system of ordinary differential equations in which the dynamics of each variable are determined by its own state and the state of its direct causes. (*E*) The example of RNA velocity is shown, with a phase portrait indicating typical trajectories according to the system of ODEs. The solid black line indicates the steady state corresponding to $u^{\left(n\right)}=\left(\gamma^{\left(n\right)}/\beta^{\left(n\right)}\right)s^{\left(n\right)}$, the dashed circles indicate equilibria for either a constant or 0 transcription rate, and the colored arrows represent typical trajectories during induction (*blue*) and repression (*red*) of transcription. (*E*) Adapted from (31).

We summarize a set of inference techniques that define varying levels of mechanistic detail but are highly interrelated and mutually reinforcing (Fig. 1). First, we review statistical relationships, in which statistical dependence, correlations, and connections to causality are of interest (Fig. 1 *A*).

Next, we discuss causal inference, in which the goal is to go beyond establishing statistical dependencies and to uncover directed interactions that reveal cause-effect relationships (Fig. 1 *B*). An important topic in developmental biology is the inference of cell-state lineages, which are ideally represented as causal graphs that link precursor cells to their descendants and will be a major focus of this section (Fig. 1 *C*). In the case of cell differentiation during development, an ideal experiment results in a time series of single-cell measurements, such as RNA sequencing profiles and/or morphological descriptors, and connections can then be made between ancestor cells at time *t* and their descendants at time t+1 using similarity measures and without ambiguity in assignment of the direction of connections (32). In cases of development, regeneration, reprogramming, or disease in which detailed time ordering or previous knowledge about lineages is unavailable (33,34), this task is more complex. In general, cell states are first connected into an undirected network using statistical similarity measures applied to expression profiles or morphological features (33) (Fig. 1 *C*). Next, directionality must be assigned to these edges to establish ancestor-descendent (cause-effect) relationships (Fig. 1 *C*). A number of techniques have been recently developed to accomplish this task, which include the use of classical causal inference techniques (35), pseudotime ordering, which relies on random-walk methods (36), and RNA velocity inference (33), although each technique has drawbacks and requires caution since time rates of change are not directly computed but rather inferred. We end this section with a review of causal inference of transcription factor (TF) and target gene interactions, which leverages an inferred cell lineage graph (37). Finally, we also discuss causal inference using light microscopy data, including a study of causal relationships among cell-cycle states and mechanisms of cell division in *E. coli* (38).

We conclude with a discussion of inference techniques that rely on dynamical systems approaches. These frameworks assume that the underlying dynamics can be expressed as a system of ordinary differential equations (ODEs), with states of the system evolving according to the ODEs along trajectories in a state space spanned by the variables of interest (Fig. 1 *D*). The ODEs can be represented by a vector field that is annotated by typical steady states, dynamics, and trajectories, altogether called a phase portrait (Fig. 1 *E*). We review several examples of inferred phase portraits in this section, but here we introduce the RNA velocity technique as a simple example that does not require time series data (Fig. 1 *E*). RNA velocity assumes simple relationships between the abundances of spliced and unspliced mRNA in cells to infer the time derivative of the gene expression state from single-cell RNA sequencing (scRNA-seq) data sets (31). Spliced and unspliced RNAs are distinguished by genomic alignment, with reads aligning to intronic references assigned to unspliced RNAs, and reads aligning to exon-exon splice junctions assigned to spliced RNAs (39). The fundamental idea behind RNA velocity is that the dynamics of spliced and unspliced mRNA abundances for a given gene can be represented by a simple set of ODEs:

(1) $$\frac{du^{\left(n\right)}}{dt}=\alpha^{\left(n\right)}-\beta^{\left(n\right)}u^{\left(n\right)},\frac{ds^{\left(n\right)}}{dt}=\beta^{\left(n\right)}u^{\left(n\right)}-\gamma^{\left(n\right)}s^{\left(n\right)}$$

where $\alpha^{\left(n\right)}$, $\beta^{\left(n\right)}$, and $\gamma^{\left(n\right)}$ are the transcription, splicing, and degradation rates of gene *n*, respectively, and $u^{\left(n\right)}$ and $s^{\left(n\right)}$ are the abundances of unspliced and spliced mRNA corresponding to gene *n*, respectively. This system defines a dynamical system that is represented by a simple phase portrait (Fig. 1 *E*). Under a constant transcription rate, unspliced mRNA levels first grow, followed by spliced mRNA levels until a steady state is reached at the line $u^{\left(n\right)}=\left(\gamma^{\left(n\right)}/\beta^{\left(n\right)}\right)s^{\left(n\right)}$ (Fig. 1 *E*). If the transcription rate is returned to 0, unspliced mRNA levels first fall, followed by spliced mRNA levels until they both return to 0 (Fig. 1 *E*) (31). The authors of the RNA velocity technique claimed that a large majority of genes obey these simple dynamics (31). Therefore, using measurements of the ratio of unspliced to spliced mRNA levels for gene *n* across cells, the ratio $\gamma^{\left(n\right)}/\beta^{\left(n\right)}$ can be estimated by choosing an ensemble of cells where gene expression levels appear to be at steady state, corresponding to cells that populate the upper-right and lower-left corners of the phase portrait (Fig. 1 *E*). Then, for each cell *i*, the RNA velocity corresponding to gene *n* can be estimated as the deviation of the observed amounts of unspliced and spliced mRNA from the inferred steady-state amounts, $v_{i}^{\left(n\right)}=u_{i}^{\left(n\right)}-\left(\gamma^{\left(n\right)}/\beta^{\left(n\right)}\right)s_{i}^{\left(n\right)}$ (39). The final result is a high-dimensional vector associated to each cell. We review a recent update to the method, which instead uses a chemical master equation (CME) formalism to model bursty transcription and discrete counts of RNA molecules, which is more biophysically realistic (39,40). However, we stress that RNA velocity infers the time rate of change of gene expression in single cells without directly measuring quantities over time. The difficulty of obtaining these direct measurements experimentally has led to ambiguity about the quality of the inferences made by RNA velocity (41), as well as other pseudotime approaches. By comparing RNA velocity estimates with simulated data, Zheng et al. have shown that data preprocessing steps using k-nearest neighbor clustering can strongly distort estimates of both the direction and magnitude of RNA velocity vector estimates (41). Therefore, caution is warranted when interpreting results from RNA velocity inferences. Finally, we conclude with a study that utilized experimental calculation of phase portraits to characterize molecular interactions during cortex activation in the *C. elegans* oocyte (13).

### Can you claim any dependents? Statistical preliminaries

The first step in understanding interactions between molecules, cells, or their associated states is to determine whether or not an interaction exists at all. The notions of statistical dependence and correlation are central, although the common phrase *correlation does not imply causation* cautions against overinterpretation. Many familiar statistical concepts are more subtle than is commonly appreciated, and we begin with a brief summary of some important points.

We focus on uncovering relationships between variables using observational data. Consider two random variables, *X* and *Y*. As an example, let *X* and *Y* be the concentrations of two proteins in a cell, and let $x_{i}$ and $y_{i}$ be simultaneous measurements of the total cellular intensities of the two fluorescently tagged proteins at a single point in time in cell *i*, normalized by the cell volume. We would like to know which details of the relationship between *X* and *Y* are discoverable from the observations. Arguably, the most important property of their relationship is whether or not *X* and *Y* are statistically dependent, since this determines if *X* and *Y* belong to a common interaction network. Our hypothetical data set consists of *N* measurements of these two proteins in *N* cells, each at the same point in time of the cell cycle. To make progress, we assume that these sets of observations are independent and identically distributed, meaning that observations in different cells originate from the same underlying processes but are subject to random variations that arise from intrinsic fluctuations in the cell due to the stochastic nature of chemical reactions and thermal noise, in additional to variations due to experimental error. Then, the collections $\left\{x_{i}\right\}$ and $\left\{y_{i}\right\}$ are *ensembles* that reflect the underlying random variables *X* and *Y*, the concentrations of the two proteins. Histograms of $\left\{x_{i}\right\}$ and $\left\{y_{i}\right\}$ will approach the probability distributions of *X* and *Y* as N→∞.

It is important to note that, despite even the most ambitious experimentalist’s efforts, *N* will always be finite and therefore any inferred information about *X* and *Y* will always be approximate. For example, we can estimate the mean of *X* by computing the sample mean $\bar{X}$ of the observations $\left\{x_{i}\right\}$. The quality of the estimate $\bar{X}$ is given by another estimate, the sample standard error of the mean:

(2) $$\sigma_{\bar{X}}=\frac{\sigma_{X}}{\sqrt{N}}$$

where $\sigma_{X}$ is the sample standard deviation. $\bar{X}\pm 1.96\times \sigma_{\bar{X}}$ is then an approximation of the 95% confidence interval for the sample mean, which can be interpreted to indicate that out of every 100 realizations of the set of observations $\left\{x_{i}\right\}$, approximately 95 of them will produce confidence intervals around $\bar{X}$ that contain the true mean of *X*. It is important to note that the error decreases as $\sqrt{N}$, which implies that a decrease by a factor of 10 of the confidence interval requires a factor of 100 more observations. Further, biological systems often exhibit large degrees of variability, which is estimated by $\sigma_{X}$ and upon which the confidence interval depends linearly. It is essential to remember that all of the approaches presented in this review must contend with finite samples and are subject to interpretation in light of approximation and error.

Regardless, we continue with the task of determining whether *X* and *Y* are dependent. There are several statistics that can be computed from the observations to determine dependence structures. The most familiar is likely the correlation between *X* and *Y*. Often, what is meant by the correlation is Pearson’s correlation coefficient, $\rho_{X,Y}$, which is defined as their covariance divided by the product of their standard deviations:

(3) $$\rho_{X,Y}\equiv \frac{\text{Cov}\left(X,Y\right)}{\sigma_{X}\sigma_{Y}}$$

Pearson’s correlation coefficient indicates the degree of linear dependence between variables, which is a weaker statement than statistical dependence. Specifically, independence of two variables always implies Pearson’s correlation coefficient is 0, but the converse is not true, since the correlation coefficient only detects linear dependence. For example, consider a random variable *X* that is symmetrically distributed around 0 and let $Y=X^{2}$. Their correlation is 0 despite a clear dependence of *Y* on *X*. Correlation is therefore a poor metric for detecting general dependence structures.

Other quantities have a closer correspondence with statistical dependence. For example, Spearman’s rank correlation coefficient is Pearson’s correlation coefficient applied to the ranks of the variables instead of their values. It quantifies the extent to which one variable is a monotonic function of the other and is not restricted to linear dependence (42). The mutual information quantifies all dependencies among two variables such that zero mutual information implies independence, and it therefore has greater descriptive power for dependence structures than correlation coefficients (27). Fundamental to the calculation of mutual information is the Shannon entropy, defined by Claude Shannon in 1948 as

(4) $$H\left(X\right)\equiv -\sum_{x\in X}p\left(x\right)\log\left(p\left(x\right)\right)$$

where p(x) is the probability distribution of *X* (43). If we use $\log_{2}$ in the definition, then the units of *H* are bits, and the entropy quantifies the expected amount of information gained (in bits) from a single observation of *X*. The mutual information between *X* and *Y* quantifies the amount of information we gain about *X* by coobserving *Y*. The mutual information is defined in terms of the Shannon entropy as

(5) I(X,Y)≡H(X)−H(X|Y)≡H(Y)−H(Y|X)

where H(X|Y) is the conditional Shannon entropy of *X* given *Y*. In practice, since we only have finite data sets $\left\{x_{i}\right\}$ and $\left\{y_{i}\right\}$, we can only compute approximations of and confidence intervals around correlation coefficients and mutual information to infer dependence between *X* and *Y*.

**Figure 2:**
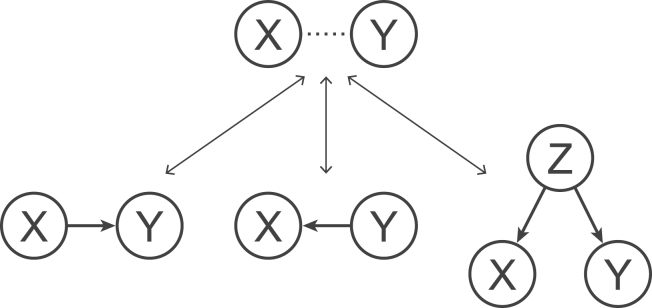
The common cause principle. If *X* and *Y* are statistically dependent, as indicated by the dashed line, then either *X* causes *Y*, *Y* causes *X*, or an unobserved variable *Z* causes both.

**Figure 3:**
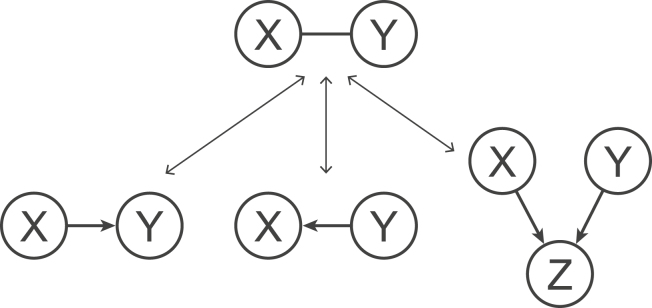
Collider bias. If *X* and *Y* are dependent after conditioning on all other variables, indicated by the solid line, then either *X* causes *Y*, *Y* causes *X*, or *X* and *Y* both cause a common effect *Z*.

If there is evidence of dependence between *X* and *Y*, an immediate implication is the existence of some causal structure as stated by Reichenbach’s common cause principle: if two random variables *X* and *Y* are dependent, then either *X* causes *Y*, *Y* causes *X*, or some other variable *Z* causes both (Fig. 2) (44). The terminology “common cause” can be understood if we regard the common cause *Z* as reducing to either *X* or *Y* in the case of a direct causal relationship between *X* and *Y*. If *X*, *Y*, and *Z* are all observed, conditional independence tests can be used to narrow down the possible causal structures, and will be discussed in the context of a recent study (38). However, conditional independence tests can introduce a new kind of spurious link, called collider bias. Collider bias states that if *X* and *Y* both cause *Z*, then *X* and *Y* are dependent after conditioning on *Z*. We are thus left with a common effect principle: two variables *X* and *Y* are dependent after conditioning on all other variables if *X* causes *Y*, *Y* causes *X*, or *X* and *Y* both cause a common effect *Z* (Fig. 3). Again, *Z* reduces to *X* or *Y* in the case of a direct causal relationship between *X* and *Y*. Conditioning therefore removes spurious links associated with common causes, but introduces new spurious links in the case of common effects. These spurious links have been cleverly leveraged to constrain causal relationships in causal inference algorithms (45), two of which are discussed in supporting material, section 1: causal inference from observations. How are the common cause and common effect principles related to the phrase correlation does not imply causation? If we take correlation to mean Pearson’s correlation coefficient, then the familiar phrase could, perhaps, be more accurately (but less memorably) restated as: correlation implies a common cause, conditional correlation implies a common effect, but the absence of correlation does not imply independence*.*

For a discussion of auto- and crosscorrelation and stationarity in time series, see supporting material, section 2: correlations and dependence among and within time series. We now review several recent studies that go beyond approximations of pairwise dependencies to infer causal interactions.

## Beyond dependence: Causal inference

A primary goal of scientific research is to identify cause and effect relationships. We represent such relationships using directed, acyclic graphs (DAGs), in which an arrow pointing from *X* to *Z* indicates that *X* causes *Z* (Fig. 1 *B*) (46,47). A causal relationship between *X* and *Z* contains more information than a dependence between *X* and *Z*. While dependence is a symmetric relation, the statement that *X* causes *Z* indicates that a perturbation of *X* will change the probability distribution of *Z*, but not vice versa. Causal inference therefore enables prediction of the effects of perturbations, while knowledge of statistical dependence alone does not.

The ability to establish causality is a contentious topic and has been debated by philosophers and scientists dating back at least to Aristotle (48). Our goal is to discuss the extent to which it is possible to infer causal relationships from observational data in the context of developmental and cellular biology. Due to all of the issues discussed above stemming from statistical dependencies and their obfuscation of causal relationships due to common causes and/or effects, it may seem like an impossible task. Also, since the goal of causal inference is to make predictions about perturbations, the most straightforward approach appears to be perturbation and experiment. It turns out that there are cases in which causal inference is possible from observational data and provides an assessment of cellular systems that does not have the risk of network rewiring entailed by perturbations. We begin by first reviewing some concepts in causality as they relate to cell biology, and proceed with examples of work in the fields of cell-state lineage inference and causal inference from microscopy data.

For a discussion of causal inference theory and algorithms, see supporting material, section 1: Causal inference from observations.

### Causal inference in the cell

The cell presents unique challenges with respect to the establishment of causality. We introduce some important concepts that help to clarify the meaning of causes and effects in the context of the cell. The following ideas are discussed in great clarity and more depth in (49,50); we briefly discuss them in the case of molecular biology.

#### Direct and indirect causes

We distinguish types of causes in terms of their proximity to an effect in a DAG. For the effect *Z*, we say that *Y* is a direct cause of *Z* and *U* is an indirect cause of *Z* (Fig. 1 *B*). In practice, these distinctions depend upon the extent to which we can actually observe *U*, *Y*, and *Z* (49). For example, if we only observe *U* and *Z*, we would infer that *U* is a direct cause of *Z*. This is related to the issue of unobserved causes discussed below.

#### Feedback

**Figure 4:**
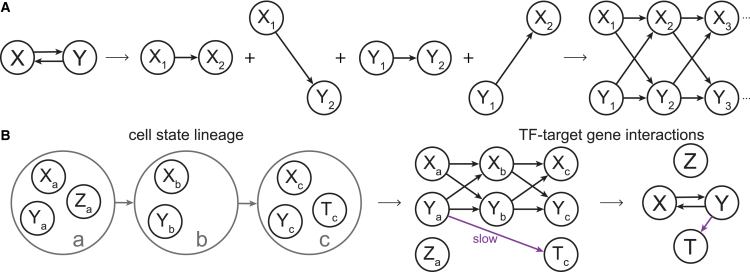
DAGs with time indices. (*A*) Without considering time, feedback structures result in cycles (*left*). However, feedback structures can be represented as DAGs if the value of each variable at discrete points in time is represented as a node and causal interactions flow forward in time. In this example, *X* and *Y* both cause each other over a time lag of 1. (*B*) TF-target gene interactions can be inferred according to a cell-state lineage (*left*). Cell states are shown as gray circles and indexed *a*–*c*, with expression of genes in each cell state. An example set of interactions are shown on the right, with genes indexed by cell state, fast interactions shown in black, and slow interactions shown in magenta. (*B*) Adapted from (37).

There are countless examples of feedback structures in molecular interaction networks. Graphically, these are represented by loops or cycles. Since causal interactions are represented by DAGs, there appears to be a problem with representation of feedback structures. However, introducing time resolves the issue by assigning a node for each variable at each point in time, which requires a time discretization (49). We can then understand feedback interactions as propagating forward through time and create DAGs for arbitrary feedback structures (Fig. 4 *A*).

#### Unobserved causes

The complexity of molecular interaction networks in cells all but guarantees the presence of unobserved variables that are relevant to any observational study. For example, a phosphorylation event early in a signaling cascade could set off a chain of interactions that ultimately lead to activation of a TF. How then do we understand causality in this context if we only observe the phosphorylation event and the TF activation, inferring a directed edge between the two? It is helpful to understand the concepts of component causes and sufficient causes (50). In general, a sufficient cause of an effect is made up of several component causes which are all necessary to cause the effect. In our example, the set of interactions in the signaling cascade comprise a sufficient cause of the TF activation, and each of the interactions are component causes. Perturbation of any of them would alter the activation of the TF. Therefore, whenever we infer a causal link, there is always the possibility that the link is indirect, and that there are hidden component causes which belong to the same sufficient cause.

#### Multicausality

Redundancy of molecular interaction networks is a widely appreciated phenomenon that ensures robustness in cell physiology (12,14). Basically, redundancy arises from multiple sets of interaction networks that can give rise to the same outcome. This reality corresponds to the concept of multicausality (50), in which one event can have multiple sufficient causes. Each of the event’s sufficient causes in general comprises several component causes, which can be shared among different sufficient causes (50). Therefore, identification of one cause does not in general rule out the possible existence of another cause. Multicausality also poses problems for the interpretation of perturbation experiments, since alteration of a redundant component may lead to the false conclusion of no causal significance when it is in reality compensated for by redundant processes.

The discussion of the above points is not intended to discourage the pursuit of understanding causality in molecular cell biology. Rather, it is to outline the extent to which we can interpret causal inferences and to encourage a broader understanding in which multiple causes and explanations can actually reflect the underlying truth of highly complex and robust systems such as living cells.

### Cell-state lineage inference

The goal of cell-state lineage inference is to identify distinct cell states or types and to connect them in a DAG that represents ancestor-descendant relationships (Fig. 1 *C*). Cell states can be defined by genomic data, such as scRNA-seq profiles, and/or by other characteristics such as cell morphology, mechanical responses, morphogen patterns, etc. Here, we discuss several cases which use genomic data, and we briefly discuss a study that generalizes the notion of cell states in the conclusion of the review.

**Figure 5:**
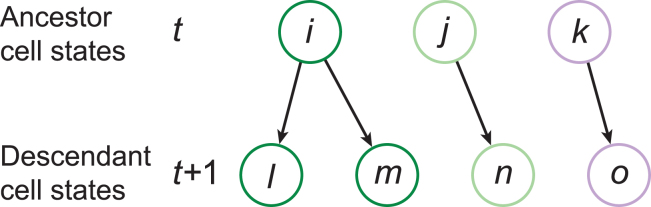
Connecting ancestor cell states with descendant cell states. Ancestor cell states at time *t* are connected to descendant cell states at time *t + 1* according to gene expression similarity, denoted by the colors of the circles. Figure adapted from (32).

The first study of interest utilized time series of scRNA-seq data that were collected for the well-studied model organism *Xenopus tropicalis* during the first day of life after fertilization (32). The authors obtained RNA expression profiles for all of the cells in *Xenopus* embryos across 10 developmental stages ranging from before the onset of zygotic transcription through the early tail bud stage when dozens of cell types have differentiated (32). After annotating 87 cell types and 259 cell states, their goal was to connect these cell states with directed edges, forming a cell-state lineage. Since their data comprised time series of observations, they limited the possible connections between cell states to point from cells at time *t* to cells at time t+1, avoiding the directionality inference problem by using direct observation (Fig. 5). Therefore, the only remaining task was to determine connections between ancestor and descendant cells. Since a descendant cell state can only arise from a single ancestor cell state, they used similarity measures among expression profiles to assign the most similar cell in time *t* to cell states in time t+1 (Fig. 5). Specifically, they first embedded all cells from adjacent time points *t* and t+1 into the principal component (PC) space arising from the t+1 cells. They then computed the Euclidean distance between cell clusters in adjacent time points and inferred a connection for the closest distances (32).

This scheme allowed for a branching architecture of the lineage tree in which branches can form going forward in time and mostly matched known lineage relationships. However, the authors found that several cell types emerged earlier in development than thought previously, including an endothelial/hemangioblast progenitor, tail bud, and several epidermal cell types (32). They conclude that this finding indicates previously unknown early transcriptional dynamics. In a companion piece (51), some of the authors applied the same technique to zebrafish embryos during development, which allowed for a comparison of the cell fate lineages between the two species. In both species, they observed a gradual increase in the complexity and combinatorial nature of TF expression that coincided with embryonic cell-state differentiation (32). However, they found large differences in tissue-specific expression of individual genes between the two species and instead found that orthologous cell states shared expression of only a subset of genes that were mostly TFs (32). With such a rich set of cell states connected by ancestor-descendant (causal) links, the authors were able to make wide conclusions about the developmental programs of two species.

Acquiring time series of organism-level scRNA-seq data during multiple developmental stages is challenging and expensive. In addition, we are also interested in systems in which lineages and cell types have not been extensively studied previously, as in regeneration, reprogramming, or disease models. Is it possible to infer such rich graphs from a single snapshot scRNA-seq data set in which time ordering and substantial previous information about the system is not known a priori? Several techniques have been recently developed to facilitate such inference. The first step is always to construct an undirected network that connects cell states based on gene expression similarity. A discussion of undirected network inference can be found in supporting material, section 3: Undirected network inference. Several approaches have been taken to assign directionality to edges. CellRank is a recently developed method that uses RNA velocity information to assign directionality (33). The rationale is that RNA velocity gives information about the likely future state of the gene expression levels in a cell and thus can be used to assign directionality to cell-state transitions. CellRank models cell-state changes as a Markov chain, in which each state is defined by a scRNA-seq profile, and transitions to descendant states only depend on the most recent ancestor state and are probabilistic. CellRank uses the inferred, single-cell gene expression vectors from RNA velocity to orient the edges detected in the previous step. The assignment is probabilistic, with neighbors who are closest to the direction indicated by the velocity vector given the higher transition probability. This is implemented by calculating the Pearson correlation, $c_{ik}$, between each velocity vector $v_{i}$ for cell *i*, and each connection $s_{ik}$ to neighboring cell *k* (33). Finally, the set of transition probabilities is defined as:

(6) $$p_{ik}=\frac{e^{\sigma c_{ik}}}{\sum_{j}e^{\sigma c_{ij}}}$$

where $\sigma =1/\text{median}\left(\left|c_{ik}\right|\right)$. The authors note, however, that the RNA velocity vectors are inherently noisy (33), which propagates into their estimates of transition probabilities. To cope with this uncertainty, they compute a weighted average of their transition probability matrix and a similarity-based transition probability matrix (33). The end result is then a weighted, directed graph connecting cell states, where edge weights correspond to transition probabilities. The authors used CellRank to identify trajectories through delta cell precursor states in murine pancreatic cells, and to predict and confirm that goblet cells dedifferentiate toward basal cells during murine lung regeneration (33).

### Gene regulatory network inference on DAGs

Establishment of cell-state lineages is valuable, but we would also like to know the extent of the interactions between certain genes in the context of gene regulatory networks (GRNs). A recently developed method, Velorama, leverages cell lineages to better infer GRNs (37). The primary goal of Velorama is to infer causal TF-target gene interactions. Velorama first requires an inferred cell-state lineage, and the authors report that using the output of CellRank leads to the best results. The algorithm then uses the lineage, a DAG, to temporally order genes with respect to one another and uses Granger causal inference to detect cause-effect relationships between genes (Fig. 4 *B*) (37). Granger causality was originally developed in the context of economics and determines causality among variables *X* and *Y* by asking whether the past history of all variables including *X* improves predictions of the current values of *Y*, relative to the past histories of all other variables without *X* (49). If the predictions of *Y* are better when including the history of *X*, then *X* Granger causes *Y*. Granger causality thus is able to distinguish direct and indirect causes when they are all observed (49). For a system with *G* variables, variable *j* is modeled as a function of the previous *L* observations of the other variables as:

(7) $$\begin{array}{l} z_{j}\left(t\right)=f_{j}(z_{1}\left(t-L;t-1\right),z_{2}\left(t-L;t-1\right),\ldots ,\\ z_{G}\left(t-L;t-1\right))+e_{j}\left(t\right) \end{array}$$

where $z_{j}\left(t\right)$ is the value of variable *j* at time *t*, $z_{k}\left(t-L;t-1\right)$ is the sequence of values of variable *k* from times t−L to t−1, and $e_{j}\left(t\right)$ is an error term (37). The authors of Velorama extend Granger causality from the typical case in which the $f_{j}$ are linear functions and all variables have a global ordering t,t+1,…, to the case of complex TF-target gene interactions on an inferred DAG. They instead model the $f_{j}$ as multilayer neural networks, and replace a global time index in the observations $z_{k}$ with ancestor-descendant lineages according to the inferred DAG (37). They are thus able to infer causal relationships between TFs and target genes, in which the set of relevant TFs for a given target gene belong to cell-state ancestors of the given target gene’s cell state. Because Velorama considers whole sets of TFs across different lag times (parent, grandparent, etc., cells in the lineage), it is able to account for multicausality (TF cooperativity) and different speeds of TF influence on target genes (37) (Fig. 4 *B*).

The authors used Velorama to study TF-target gene interactions in a data set comprising paired scRNA-seq and chromatin accessibility data of human fetal cortical samples during midgestation (37). They first demonstrated that their inferred speeds of TF action on target genes were verified by the chromatin accessibility data (37). They next found that the fast TFs were mostly specifically expressed in brain cells, while the slow TFs were generally expressed in a broader set of cell types. They also found that fast TFs are more implicated in neuropsychiatric disorders, while the slow TFs are more implicated in the formation of gliomas. They hypothesize that the specific expression patterns of the fast TFs are consistent with neural diseases, while the more general expression of the slow TFs are consistent with more systemic diseases such as cancer (37). Finally, they analyzed the degree of cooperativity among TFs, finding that TFs of different speeds often cooperate. They identified a specific system, which comprises the slow TFs SATB2 and HMGB2, which are chromatin remodelers that cooperate with the fast TFs BCL11B and EOMES, which drive cell fate changes during differentiation (37). Velorama therefore leverages two levels of causal inference, first creating cell-state lineages that restrict the space of possible causal interactions among the second layer of TF-target gene interactions. The identification of causal, cooperative TF-target gene interactions promises to reveal deep insights into gene regulation during development and disease.

### Causal inference using microscopy data

We now review a recent study that leveraged causal inference using microscopy data (38). Kar et al. used simple conditional independence tests to infer causal relations among stages of the cell cycle in *Escherichia coli* (38). The research leverages structural causal models (SCMs) (44,52), which naturally follow from DAGs and which we define now. For the example DAG:

(8) X→Y→Z,A→Y

the corresponding SCM is:

(9) $$\begin{array}{l} X=f_{X}\left(\eta_{X}\right),Y=f_{Y}\left(X,A,\eta_{Y}\right)\text{,}\\ A=f_{A}\left(\eta_{A}\right),Z=f_{X}\left(Y,\eta_{Z}\right) \end{array}$$

where the $\eta_{i}$ are independent noise terms. In an SCM, each variable is a function of its own noise term and its direct causes (44). A new feature of SCMs relative to DAGs are the explicit noise terms, which reflect the reality that each node in the DAG is a random variable. The assumption of independent noise terms implies that all common causes are observed, because if the noises were dependent, their associated variables would have some unobserved common cause. Using specific functional forms of the $f_{i}$ allows one to make predictions about perturbations of any variable and to choose the most appropriate conditional independence tests for inference.

**Figure 6:**
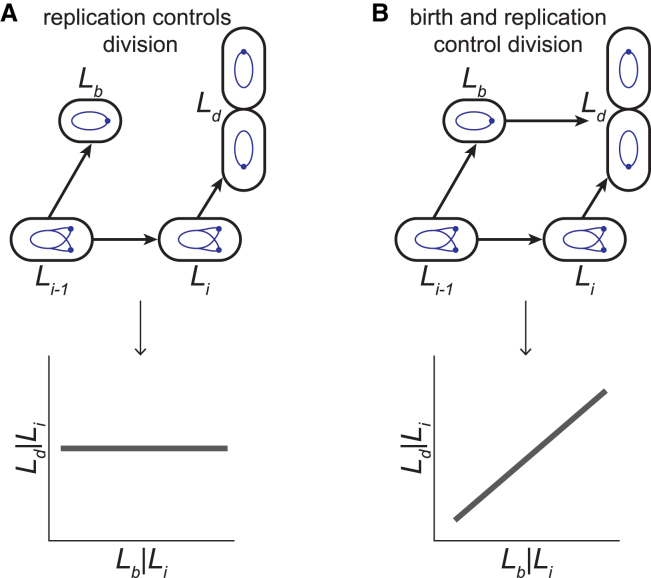
Conditional independence tests to distinguish models of *E. coli* cell-cycle control. (*A*) Causal links are shown in the model in which only replication cues control division (*top*). $L_{i-1}$ is the length of cells during the previous replication initiation event, $L_{i}$ is the length during the current initiation event, $L_{b}$ is the length at birth, $L_{d}$ is the length at division. Based on the causal graph, there is no expected correlation between $L_{b}|L_{i}$ and $L_{d}|L_{i}$. (*B*) The causal graph corresponding to the model in which cues from both birth and replication control division is shown (*top*). For this model, there is a positive expected correlation between $L_{b}|L_{i}$ and $L_{d}|L_{i}$. Figure adapted from (38).

In (38), the authors observed the growth of *E. coli* in media, which promotes either slow or moderately fast growth, and collected data about the lengths of cells at birth $L_{b}$, initiation of DNA replication $L_{i}$, and division $L_{d}$, as well as during other cell-cycle events. They set out to compare several competing models of cell-cycle regulation that posit distinct causal interactions among these events, resulting in different DAGs. To discriminate among the models, the authors found pairs of variables that should be conditionally (in)dependent given a DAG (Fig. 6) (38). The authors assumed that the functional forms of the SCMs, which are implied by their DAGs are linear and can be interpreted as Taylor expansions around the average nonlinear interactions among cell-cycle events (38). They also assumed that the noise terms are independent and Gaussian. These assumptions allowed them to test for conditional independence simply using linear regression and Pearson correlations of the resulting residuals. For example, to discriminate among a model in which only replication cues control cell division and a model in which both cues at cell birth and replication control cell division, they calculated the conditional correlation $r\left(L_{b},L_{d}|L_{i}\right)$ by obtaining the residuals after linear regression of $L_{b}$ on $L_{i}$ and $L_{d}$ on $L_{i}$ and then obtaining the Pearson correlation between those residuals (Fig. 6). The presence or absence of a statistically significant correlation then serves as a test for conditional independence of $L_{b}$ and $L_{d}$ given $L_{i}$ and allowed the authors to infer causal relationships among cell-cycle events (38). They found that the model shown in (Fig. 6 *A*) is consistent with observations in slow growth media, while the model shown in (Fig. 6 *B*) is consistent with observations in fast growth media. The authors investigated several other relationships and proposed a few possible molecular mechanisms consistent with their inferred models for future study.

Causal inference aims to answer questions about directional interactions and can account for the challenges of common causes and spurious interactions. However, in addition to inferring the presence and direction of interactions, we would like to understand the dynamics of the interactions ideally as explicit functions. Although methods have been proposed to quantify the strength of causal links (53), we next focus on an alternative framework that relies on dynamical systems theory and models interaction networks as systems of ODEs.

## Cellular phase space: Dynamical systems approaches

**Figure 7:**
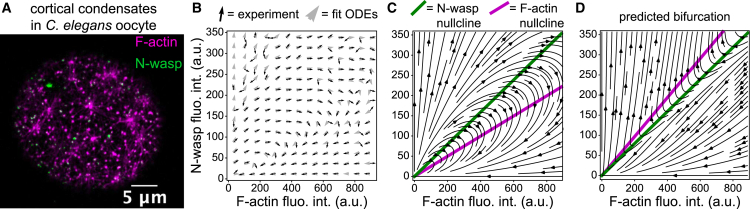
Cortical condensates and their phase portraits. (*A*) Cortical condensates are shown in a *C. elegans* oocyte. F-actin is labeled with LifeactmKate2 and N-wasp is labeled with eGFP. (*B*) The experimentally inferred phase portrait is shown by black arrows that represent normalized velocity vectors calculated from experimental time series of N-wasp and F-actin fluorescence intensities in thousands of cortical condensates. The gray arrows are vectors calculated from the fit system of ODEs (13). (*C*) The streamlines and N-wasp and F-actin nullclines are shown for the system of ODEs. The fixed point at (0,0) is a saddle point that is stable in the F-actin direction and unstable in the N-wasp direction. (*D*) The streamlines and N-wasp and F-actin nullclines are shown for the system of ODEs after a bifurcation due to increased $k_{d}$.

This final section reviews techniques that are based on dynamical systems. A dynamical system is a system of coupled ODEs that represents the interactions among a set of variables through time. The framework of dynamical systems allows us to regard the state of a system as a point in a phase space in which each coordinate represents a variable of interest. The ODEs define a velocity vector at each point in phase space, which we can follow from any given initial condition (initial point in phase space) to construct a trajectory (Fig. 7, *B* and *C*). Trajectories can correspond to arbitrary processes that evolve in time, including chemical reactions, patterns of neuron activation, populations of species, etc. There is a long history of regarding biological systems as dynamical systems (54), ranging from descriptions of molecules, to cells, to ecosystems. A benefit of a description that comprises ODEs is that interactions are captured in mathematical form, enabling quantitative predictions. There is also a vast literature related to long-term behaviors of dynamical systems which can either settle into one of a handful of known patterns or display chaotic behavior (54,55,56). Phase portraits are a particularly useful way of describing behaviors of dynamical systems (Fig. 7, *B* and *C*) and will be discussed in more detail as inference tools shortly (13,54,57). Finally, the evolution of steady states of a given variable with respect to a given parameter in the dynamical system is summarized by bifurcation diagrams (55). Some authors have argued that there is a qualitative correspondence between signal-response curves and bifurcation diagrams (55). Dynamical systems thus provide researchers with a wide array of established mathematical tools and offer the most precise descriptions of interactions discussed thus far. Ultimately, comprehensive descriptions require that the ODEs are placed in space and generalized to systems of partial differential equations (PDEs). Spatial inference techniques are outside the scope of this review but will be essential for complete understanding. We therefore restrict our focus to inference that relies on ODEs and provide some references for inference of PDEs and spatial causal inference. We begin with a discussion of recent updates to RNA velocity and end with a study that leveraged experimental inference of a phase portrait to learn the governing equations of F-actin and N-wasp dynamics during cortex activation in *C. elegans* oocytes.

### RNA velocity as a discrete Markov process

As introduced previously, RNA velocity depends on estimation of the levels of unspliced and spliced mRNA from scRNA-seq data and makes predictions about their future values by fitting to a simple dynamical system (Fig. 1 *C*). The original approach (31) and an updated approach called scVelo, which allows for dynamic transcription rates (58), both assume smooth, deterministic dynamics of mRNA counts in cells. However, it is well known that mRNAs often have low copy numbers in which discrete descriptions are superior (39). In addition, transcriptional dynamics are known to be bursty, in contrast to the smooth continuous functions posited in the previous examples (39). Therefore, Gorin et al. argue for a discrete, Markov process model which is described by a CME (39). The CME describes the flux of probabilities between the states of variables with integer counts and is an infinite series of coupled ODEs (39). The dynamics given in the original RNA velocity paper can be described as:

(10) $$\emptyset \overset{\alpha \left(t\right)}{\to }u\overset{\beta }{\to }s\overset{\gamma }{\to }\emptyset$$

which implies the following CME:

(11) $$\begin{array}{l} \frac{dP\left(u,s;t\right)}{dt}=\alpha \left(t\right)\left[P\left(u-1,s;t\right)-P\left(u,s;t\right)\right]+\\ \beta \left[\left(u+1\right)P\left(u+1,s-1;t\right)-uP\left(u,s;t\right)\right]+\\ \gamma \left[\left(s-1\right)P\left(u,s-1;t\right)-sP\left(u,s;t\right)\right] \end{array}$$

where α(t) is a piecewise constant transcription rate, β is the splicing rate, γ is the degradation rate, and P(u,s;t) is the probability of the state with *u* unspliced transcripts and *s* spliced transcripts at time *t* (39). This model more accurately represents low copy numbers and the intrinsic noise in transcription and splicing reactions (39). To incorporate bursty transcription dynamics, the authors have recently introduced a new model and inference method (40). Their model can be represented as:

(12) $$\emptyset \overset{\alpha }{\to }B\times u\overset{\beta }{\to }s\overset{\gamma }{\to }\emptyset$$

where transcriptional events occur as a Poisson process with rate α, *B* is the burst size during a transcriptional event and is a geometric distribution with mean *b*, and β and γ are defined identically as in the previous model (40).

Because no closed-form solution to the corresponding CME exists, the authors use neural networks to learn approximate solutions (40). With approximate solutions to the CME, they infer the biophysical parameters corresponding to burst size *b*, and relative splicing and degradation rates β/α and γ/α, respectively, using maximum likelihood estimates from scRNA-seq data (40). The authors show that their inference method recovers the same biophysical parameters from a mouse primary motor cortex scRNA-seq data set as accurately as previously developed CME-approximation methods, but orders of magnitude faster. They note that, because their approach can flexibly infer parameters from multivariate systems, it could be adapted to interpret results from ATAC-seq experiments in which both chromatin accessibility and RNA counts are available (40). These methods, which use CME representations of RNA dynamics, can more faithfully represent the underlying biophysical processes and will likely improve RNA velocity estimates, although the inference strategies are significantly more complex. However, the incorporation of RNA velocity estimates can provide valuable information to infer the direction of edges in cell-state lineage inference (33) and inference of GRNs (37), so their accuracy is a priority.

### Cortical condensates

Our final example demonstrates the use of phase portraits calculated from fluorescence microscopy time series data sets. In (13), the authors set out to understand growth dynamics of cortical condensates, transient assemblies of F-actin, N-wasp, and Arp2/3 that form in the actomyosin cortex of *C. elegans* oocytes and embryos during activation of the cortex. F-Actin and N-wasp were fluorescently labeled, which allowed the authors to track the amount of each molecule over time in thousands of individual condensates (Fig. 7 *A*). Using this large ensemble of coupled time series, they constructed a phase portrait by calculating the average time rate of change of F-actin and N-wasp as a function of the amount of F-actin and N-wasp in the condensate (13). In this framework, cortical condensates are represented as a 2D dynamical system whose phase space is defined by the numbers of F-actin and N-wasp molecules in the condensate at a given time. The average time rates of change computed from the time series then constitute vectors associated to each neighborhood in phase space (Fig. 7 *B*). Altogether, the phase portrait indicates that cortical condensate growth dynamics are well described by homoclinic orbits, in which N-wasp and F-actin first grow, N-wasp then falls while F-actin continues to grow, and finally both fall until the condensate disappears (13). Therefore, any system of equations that describes these dynamics must recapitulate such homoclinic orbits.

To find these equations, the authors used the observation from the phase portrait that cortical condensate dynamics appeared to depend on the volume fraction of F-actin and N-wasp within condensates. They plotted the relative growth rates of N-wasp, $\dot{W}/W$, as a function of the F-actin volume fraction, $\phi_{A}=v_{A}A/V$, where $\dot{W}$ is the time derivative of N-wasp, $v_{A}$ is a scalar that relates F-actin fluorescence intensity to molecular volume, and *V* is the time-dependent condensate volume. A similar comparison was made for relative F-actin growth rates versus N-wasp volume fractions. Plots of the data revealed linear relationships, i.e.,

(13) $$\frac{\dot{W}}{W}=k_{r}-\frac{k_{f}}{v_{A}}\phi_{A},\frac{\dot{A}}{A}=\frac{k_{l}}{v_{W}}\phi_{W}-k_{d}$$

where $\phi_{W}=v_{W}W/V$ is the volume fraction of N-wasp and $v_{W}$ relates N-wasp fluorescence intensity to molecular volume, and the $k_{i}$ are rates determined by linear fits. Rearranging these equations reveals a nonlinear dynamical system that describes the growth dynamics of cortical condensates and correctly produces the homoclinic orbits described by the inferred phase portrait (Fig. 7 *B*) (13):

(14) $$\dot{W}=k_{r}W-k_{l}\frac{AW}{V},\dot{A}=k_{b}\frac{AW}{V}-k_{d}A$$

These equations represent effective descriptions of the interactions between F-actin and N-wasp in cortical condensates, which coarse-grain away other molecules that are present, including Arp2/3, which is nucleated by N-wasp and promotes F-actin branching, and other actin regulators.

With this description, the authors observed oocytes under mild and moderate Arp2/3 knockdown by RNAi and refit the model parameters. They found that the dominant effect was the F-actin depolymerization rate $k_{d}$ increased as Arp2/3 levels were reduced (13). For larger increases of $k_{d}$ the system of ODEs exhibits a bifurcation in which the homoclinic orbit is replaced by unbounded growth due to the F-actin nullcline moving above the N-wasp nullcline (Fig. 7, *C* and *D*) (13). To test this prediction, the authors used very strong knockdowns of Arp2/3 and observed that cortical condensates disappeared and were replaced by large stable patches of F-actin and N-wasp, as predicted by the phase portrait after the bifurcation (Fig. 7 *D*). In this study, inference of the interaction dynamics before perturbation enabled a precise understanding of the system once perturbations were performed, which would not have been interpretable otherwise, since the perturbation destroyed the cortical condensate dynamics. Investigation of fluctuations in this system and their effects on phase portrait inference due to multiplicative noise (59,60) are the subjects of ongoing research. Also, we note similarities in the molecular components and the temporal ordering of their recruitment in cortical condensates to those observed in actin patches associated with sites of endocytosis (61) and podosomes (62). It is possible that cortical condensates may share features of broadly conserved mechanisms of branched actin growth and/or are implicated in other cellular processes.

Further discussion of dynamical systems inference algorithms and the connections between dynamical systems approaches and causal inference can be found in supporting material, section 4: dynamical systems.

## Conclusion and future outlook

All of the previously described techniques have advantages and drawbacks, which we briefly summarize. Causal inference provides more precise information than statistical dependencies and correlations, including directed interactions, systematic methods for removing spurious links, distinguishing between direct and indirect causes, and accounting for unobserved variables. However, the precise nature of the interactions are not provided by causal inference. On the other hand, dynamical systems techniques provide the most detailed descriptions of interactions as systems of ODEs. They require the most domain knowledge and their results are dependent upon the specific variables chosen to represent phase space. They do not explicitly account for causal interactions and thus interpretation of their results in the causal context is not always clear.

We therefore suggest that a possible fruitful path of research is to combine the benefits of causal inference with dynamical systems approaches, as was demonstrated with CellRank in which RNA velocity estimates, which leverage dynamical systems approaches, were used to infer directionality in cell-state lineages. On the other hand, causal inference could precede more detailed characterization. Once a DAG has been inferred, phase portraits or equation discovery techniques could be used to learn the governing equations of those direct interactions. In these ways, the benefits of both approaches are leveraged. The following study takes the latter approach. In (63), the authors take as input a directed network topology and time series data and use dynamic network theory to learn the equations governing the interaction dynamics and to refine the network topology estimation. Another example establishes a connection between a dynamical systems approach and DAGs to understand cell-state lineage and GRN inference (64). The authors use an abstract representation of cell states that can apply equally well to GRNs and, using geometric descriptions of systems of differential equations, they enumerate all ways in which three-way state transitions can be made with at most two tunable parameters (64). They show a correspondence of these state transitions with DAGs, in which transitions correspond to bifurcations of the dynamical system. Finally, they suggest methods for dimensionality reduction such that their methods can apply to the complex settings in developmental biology, and suggest techniques to fit to time-lapse data. Further studies in these directions are warranted.

Other important advances will include extending these techniques to incorporate spatial information and stochasticity. Recent work on spatial causal inference (65) and PDE learning (66,67,68) will be critical in the endeavor to incorporate spatial information and arrive at a complete description of cells. The CME approach described in RNA velocity inference (40) and techniques for inferring noise structures from data (69) will be essential in developing full pictures of the stochastic processes that ultimately underlie all of cell biology.

It is the authors’ view that inference of interaction networks using the described approaches will be essential to gaining a physical understanding of cellular and developmental biology. Inference from observational data using any or all of the techniques discussed in this review should ideally precede interpretation of perturbation experiments whenever possible. In this manner, researchers can build up models that have as much detail as possible and make precise predictions. Such inferences will minimize the risk of network rewiring entailed by perturbative experiments. Of course, perturbations will be necessary to confirm any inferences but, with a robust set of predictions from observational data, results of perturbation experiments can be framed within their context and interpreted to the greatest extent possible. Also, time will be saved since resulting experiments will address focused, precise questions. As progress in observational and analysis capabilities continues, an unprecedented understanding of developmental and cellular biology is sure to follow.

## Acknowledgments

S.W.G. and I.S. acknowledge support from the 10.13039/501100004189Max Planck Society, and I.S. acknowledges support from the 10.13039/100005156Alexander von Humboldt Foundation.

## Author contributions

I.S. and S.W.G. wrote the paper.

## Declaration of interests

The authors declare no competing interests.

## Supporting material

Document S1. Figures S1–S5

Document S2. Article plus supporting material
