# Supplementary material for: Empirical methods that provide physical descriptions of dynamic cellular processes
Source: Biophys J. 2024 Dec 4;124(6):861–75. doi: 10.1016/j.bpj.2024.12.003 (PMC11947468; doi:10.1016/j.bpj.2024.12.003)
Supplement: Document S1. Figures S1–S5 [file mmc1.pdf]

Biophysical Journal, Volume *124*

Supplemental information

Empirical methods that provide physical descriptions of dynamic cellular processes

Ian Seim and Stephan W. Grill

Supplemental Information for: Empirical methods that provide physical descriptions of dynamic cellular processes

Ian Seim[1,*] and Stephan Grill[1,2,3,*]

[1]Max Planck Institute of Molecular Cell Biology and Genetics, Dresden, Germany
[2]Center for Sytems Biology Dresden (CSBD), Dresden, Germany
[3]Cluster of Excellence Physics of Life, TU Dresden, Dresden, Germany
[*]Correspondence: seim@mpi-cbg.de, grill@mpi-cbg.de

Contents

1 Causal inference from observations **2**
1.1 Causal inference in the presence of unobserved variables 2
1.2 non-linear SCMs with non-Gaussian noise and do-Calculus 3
1.3 Causal inference algorithms for time series data 4

2 Correlations and Dependence among and within Time Series **5**

3 Undirected network inference **6**
3.1 Network inference for data collected at a single time point 6
3.2 Inference for undirected time-varying networks using time series data 7

4 Dynamical Systems **9**
4.1 Relationships between dynamical systems approaches and causal inference 9
4.2 Leveraging dynamical systems theory to learn DAGs 9
4.3 Automated discovery of differential equations 10

1 Causal inference from observations

A central goal of causal inference is to establish the smallest set of possible DAGs that are consistent with the conditional independence patterns in a dataset, called a *Markov equivalence class* [1]. As we have seen, major issues for determining causal relations are determining the direction of interactions and dealing with spurious links arising from common causes (**Fig 2**) and common effects (**Fig 3**). The fundamental advance of causal inference is to consider conditional dependence between pairs of variables given appropriate *subsets* of other variables in the network. We briefly discuss some strategies that use such statistical tests to address the challenges of inferring directional interactions and removing spurious links due to common effects and unobserved common causes.

The first approach, called the PC algorithm [1], addresses common effects and assumes *causal sufficiency*, which requires that all common causes are observed. It fundamentally relies on the concept of *d-separation* which defines conditional independence between variables dependent on subsets of other variables in the network. Specifically, X and Y are d-separated given the set of variables $\boldsymbol{W}$ which does not contain X or Y iff X and Y are conditionally independent given $\boldsymbol{W}$ [1]. The PC algorithm works by starting with an undirected graph in which all nodes are connected (**Fig S1A**). Each pair of nodes X and Y is evaluated with respect to sets of other nodes, $\boldsymbol{W}$, to find if they can be d-separated conditional on $\boldsymbol{W}$, where $\boldsymbol{W}$ iteratively contains $0, 1, 2, \ldots$ nodes. If there is any $\boldsymbol{W}$ including the empty set for which X and Y are d-separated, the edge between them is removed. This step removes links in which nodes are independent, when they are dependent due to common and indirect causes, is not subject to spurious links due to common effects, and leaves an undirected graph in which all edges correspond to direct causal interactions (**Fig S1B**). Next, as many edges as possible are oriented. For all triplets X, Y, and Z in which X, Z and Y, Z are adjacent, but X, Y are not, then $X \rightarrow Z \leftarrow Y$ if X and Y are not d-separated for every subset of nodes $\boldsymbol{V}$ which contains Z. This step correctly identifies common effect topologies (**Fig S1C**). The final step relies upon the remaining possible topologies given proper identification of all common effects in the previous step. Specifically, if $X \rightarrow Z$, Z is connected to U, and X is not connected to U, then $Z \rightarrow U$ (**Fig S1D**). This inference is possible because the conditional independence tests used in the previous step to identify $X \rightarrow Z \leftarrow Y$ would not have yielded such a topology if $U \rightarrow Z$ [1]. Common effects are therefore the basis upon which the PC algorithm assigns directionality to interactions. This procedure results in a graph with a combination of directed and undirected edges that represents a family of graphs which are all compatible with the data, since undirected edges can be oriented either way and still explain the data. Such a graph is called a Markov equivalence class. No links are spurious and each explains a direct causal interaction, so the PC algorithm is a major advance over the previous approaches discussed in this review.

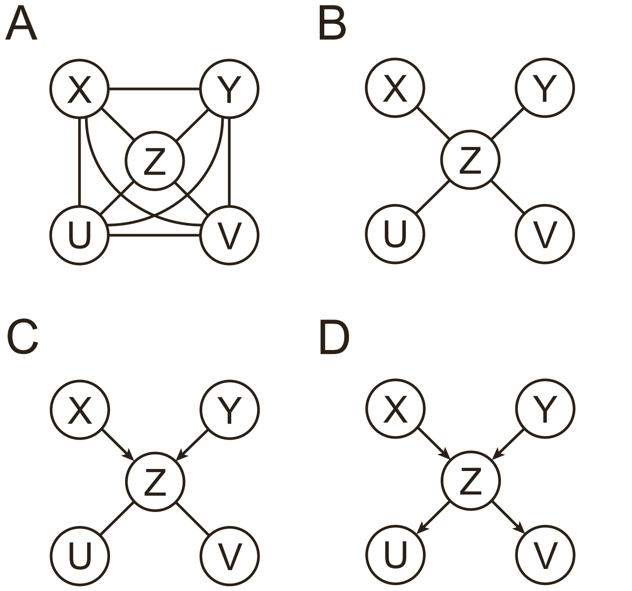

Figure S1: **The PC algorithm.** The DAG to be inferred is shown in panel D. A) The algorithm begins with all nodes connected by undirected edges. B) Conditional independence tests leave the causal skeleton. C) All colliders are oriented. D) Edges connecting colliders to their descendants can be oriented.

1.1 Causal inference in the presence of unobserved variables

The PC algorithm assumes causal sufficiency in which all common causes are observed. However, this condition is often not met in practice. How can we cope with unobserved common causes? In particular, if we detect that X and Y are dependent, but have not measured some common cause Z, how do we determine whether or not Z exists or if there is a causal relation among X and Y using only observations of X and Y? A possible solution is the fast causal inference (FCI) algorithm, introduced in [1]. The underlying concepts and algorithm implementation are too complicated to discuss in detail here and can be found in Chapter 6 of [1]. The output of the FCI algorithm is a graph with 4 edge types that denote the following: causal (direct) interactions, undirected interactions, an edge which indicates either a direct interaction or a common cause, or an edge which indicates the presence of an unobserved common cause for pairs of variables between which there is no direct interaction (**Fig S2**). Although unobserved variables reduce the certainty in the inferences, it is remarkable that such details about interactions can still be inferred, and that unobserved common causes can be definitively detected (and their measurement possibly prioritized for future experiments).

Further assumptions about the functional forms of the interactions among nodes can go further than con-

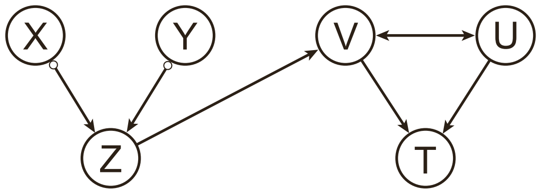

Figure S2: **Example output of the FCI algorithm.** The edge from X to Z indicates either a direct interaction or a common cause, the edge from Z to V indicates a direct interaction, and the edge between V and U indicates the presence of an unobserved common cause and *no* direct interaction between V and U.

ditional independence tests in determining network structures in the presence of unobserved variables. In Chapter 6 [1], the authors discuss some situations assuming linear relationships in which analysis of specific combinations of correlations among variables, called tetrad differences, allows for the inference of the presence and structure of unobserved variables. Finally, the concept of *instrumental variables* can provide avenues for making causal inferences in the presence of unobserved variables. If we wish to identify whether or not X causes Y in the presence of unobserved variables $\boldsymbol{U}$ which may cause both X and Y, the variable Z is an instrumental variable if it causes X but not Y and shares no common causes with Y (**Fig S3**). Then the effect of X on Y can be estimated by the ratio of the effect of Z on Y to the effect of Z on X, even in the presence of the unobserved variables $\boldsymbol{U}$ [2]. The use of instrumental variables is subject to certain assumptions and their identification is non-trivial [2].

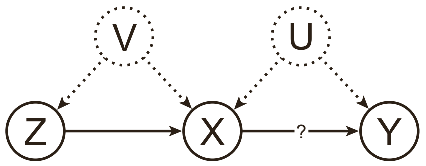

Figure S3: **Instrumental variables.** Z is an instrumental variable since it directly causes X and has no common causes with Y, and can help to answer whether X causes Y. V and U indicate unobserved common causes. Note that Z can share common causes with X.

All of the methods discussed above rely on conditional independence tests, which are subject to error with small amounts of data and noise. In practice, testing for conditional independence can be challenging, but partial correlation coefficients, conditional mutual information, or other techniques that best capture the dependence structures in the data are available [3, 4]. However, inference can be bolstered with previous knowledge about the presence/absence and direction of edges among nodes. Any information about time ordering of variables can constrain conditional independence tests (if Y occurs after X, then X should not be conditioned on Y) and help to determine directionality of edges (if X and Y are connected and Y occurs after X, then $X \to Y$) [1]. The PC and FCI algorithms are also feasible for large numbers of variables if the underlying graphs are sparse, which we have already established is the norm for biological networks [5, 6, 7].

1.2 non-linear SCMs with non-Gaussian noise and do-Calculus

Kar *et al* assumed linear relationships and additive Gaussian noise in building SCMs, but many processes are not well-described with linear functions or Gaussian noise. Therefore, there have been several efforts to extend techniques to non-linear [8] and non-Gaussian [9] SCMs. The authors of both of these studies find that non-linearity and non-Gaussianity can actually constrain the plausible set of causal models which fit the data and even lead to identification of unique models, as opposed to the Markov equivalence classes that are generally the output of the PC and FCI algorithms. The central idea is that both non-Gaussian noise and non-linearity can help to determine the direction of interactions, whereas the PC algorithm can only assign directions using common effects and their descendants [8, 9, 1].

Structural causal models are central to causal inference because they allow for prediction of the effects of perturbations and for reasoning about hypothetical situations. A primary set of relevant analysis tools is *do-Calculus*, invented by Judea Pearl [10]. do-Calculus allows one to compute distributions of Y given that X is set to a value x in a structural causal model, i.e. $P(Y|\text{do}\, X = x)$, which is in general not the same as $P(Y|x)$ [11]. However, according to Pearl's causal hierarchy [10], there is a level of causal reasoning that subsumes even prediction of the effects of perturbations, called *counterfactual* reasoning. Counterfactuals are statements like "If x had happened, then y would have been different." They are retrospective hypotheticals which use present observations to reason about alternative scenarios. Therefore, they can leverage more information since they include information given observations. One can create counterfactual SCMs by conditioning the noise terms on the given observations x and then performing the do-Calculus operation one is interested in [12, 11]. Since the conditioning only affects the noise variables, the underlying DAG is unaltered, but the effects of perturbations given our current knowledge from observations is in general different than when using only prior distributions for the noise [12]. A recent study used counterfactuals and do-Calculus to study Zebrafish development [13]. The authors identified molecular mechanisms for activation of two Zebrafish *nodal* genes that control mesoderm and endoderm induction using do-Calculus to analyze fluorescence in situ hybridization (FISH) images of the genes of interest [13]. SCMs and counterfactual SCMs are extremely important tools in causal inference, and recent efforts to incorporate them into machine learning algorithms are the foundation of a new field called

causal machine learning [11, 14]. The hope in causal machine learning is that incorporation of causal inference can allow for more precise interpretations of and predictions from data than the standard statistical associations produced by most machine learning models.

1.3 Causal inference algorithms for time series data

Causal inference using time series data becomes both easier and more difficult due to time ordering of variables. Since causality can only flow forward in time, the space of possible directional interactions is smaller in one sense. However, since variables can be represented as a series of nodes at each time point (**Fig 4**), inference of causal links between variables at specific time lags is desired which expands the space of possible connections. Additionally, many inference techniques rely on the assumption of independent noise terms, but many real-world time series are auto-correlated which violates that assumption. Stationarity is also a common assumption of causal inference techniques for time series that is often violated by real time series. Therefore, there are extra precautions that must be taken when working with time series.

There are many approaches to causal inference for time series including generalization of the DAGs and SCMs discussed in the previous section and generalizations of information theoretical concepts, among others. We will briefly discuss some of these approaches, beginning with DAGs and SCMs. More extensive reviews of causal inference for time series can be found in [15, 16].

A recent approach from Runge *et al* relies on SCMs generalized to stochastic processes and uses variants of the PC algorithm and conditional independence tests to infer causal links in auto-correlated, non-linear time series [17]. Specifically, the first step of their algorithm PCMCI uses a variant of the PC algorithm to create a subset of possible direct causes for a given node X_t in a way analogous to the procedure described above. The second step uses momentary conditional independence (MCI) tests to further trim the set of possible direct causes and accounts for auto-correlations [17]. The specific conditional independence test can be chosen by the user, and the authors reviewed the performance of partial correlations, Gaussian process regression, and conditional mutual information. The latter two can detect non-linear dependencies but have lower power to detect linear relationships for small sample sizes [17]. The authors show that PCMCI can correctly reconstruct the Walker circulation, a well-understood model of air flow in the tropics, from observational climate data. They also demonstrate the algorithm's efficacy on data of heart rate and blood pressure and on large synthetic datasets. The PCMCI algorithm assumes causal sufficiency and stationarity, although it was able to correctly infer causal links even in the presence of known non-stationarity. To relax the assumption of causal sufficiency, the LPCMCI was developed [18]. It is based on variants of the FCI algorithm introduced previously and can thus account for unobserved variables. However, it relies heavily on stationarity. Several alternative techniques have been proposed to account for and even exploit non-stationarity for inference [19, 20, 21].

An alternative approach to causal inference for time series generalizes the concept of conditional mutual information to variables separated in time, and is called the *transfer entropy* [22]. The transfer entropy measures the amount of information transfer from X_t to Y_t and can be defined as a conditional mutual information:

$$T_{X\to Y} = I(Y_t; X_{t-1:t-L} | Y_{t-1:t-L}) \tag{1}$$

where $X_{t-1:t-L}$ are the values of X_t at times $t-L, t-L+1, \ldots, t-1$ and similarly for Y_t. Therefore, the transfer entropy is the mutual information between Y_t and past values of X_t conditioned on past values of Y_t. It is an inherently asymmetric measure of information flow due to the inclusion of time lags, but as a measure of information flow includes information from not only direct causes but also indirect causes and common causes. Therefore, for accurate causal inference, additional steps must be taken in addition to computing the transfer entropy among pairs of variables. However, it provides a quantification of information transfer between variables which is an advantage over previous methods. The authors in [23] estimate transfer entropy to measure information flow between the cell signaling proteins SOS and RAF. They recorded time series of the intensities of fluorescently labeled SOS and RAF in HeLa cells upon activation of the upstream cell surface receptor protein ERBB with epidermal growth factor (EGF). They studied the wild-type system and a mutant system in which SOS has a point mutation associated with Noonan syndrome with the goal of identifying a malfunctioning molecular mechanism underlying the disease. The authors assume that pairs of time series can be regarded as multivariate Gaussian distributions at each time, which is a valid assumption in the case of Gaussian noise [23]. This assumption allows them to compute transfer entropy using covariance matrices and allows for non-stationarity if the noise is Gaussian. Using this approach in wild-type cells, they detect a flow of information first from SOS to RAF, and later from RAF to SOS, consistent with the known presence of a negative feedback mechanism. In the mutant cells, however, they detected information flow only from SOS to RAF, suggesting that the mutant SOS abrogates the negative feedback mechanism [23]. Although their method does not account for the possibility of unobserved common causes, it does generate predictions that can be directly tested. Other approaches have also successfully measured transfer entropy in non-stationary time series using ensembles of measurements [24, 25].

2 Correlations and Dependence among and within Time Series

In practice, we often have the advantage (complication) of working with datasets which comprise time series of multiple variables of interest. As an example, we can imagine that we measure the total cellular fluorescence intensities of two tagged proteins, x and y, at a series of times, $t_0, t_1, t_2, \ldots, t_n$. For cell i, we now have two time series corresponding to observations of the two proteins, $x_i^t = \{x_i(t=0), x_i(t=1), x_i(t=2), \ldots, x_i(t=n)\}$ and y_i^t defined analogously. If we record time series for N cells and manage to temporally align them among cells (a non-trivial task), we now have an ensemble of time series $\{x_i^t\}$ and $\{y_i^t\}$. These ensembles can be regarded as realizations of the stochastic processes, $\{X_t\}$ and $\{Y_t\}$. These objects are collections of the random variables X_t and Y_t, which are generalizations of our previous variables X and Y to the times $t_0, t_1, t_2, \ldots, t_n$. X_{t_i} can have a different probability distribution than X_{t_j}. In our example of protein expression, time t_i could be during the G_1 cell cycle phase, and the time t_j could be during S phase. It is likely that large-scale rearrangements of gene regulatory networks have occurred in this time interval leading to a different expression pattern for our protein, and thus distinct probability distributions for its expression at times t_i and t_j. It is not hard to imagine that such a feature complicates analysis of time series and compromises resulting predictions. Therefore, a common requirement for time series analysis is the assumption that the underlying stochastic processes are *stationary*, i.e. for the stochastic process $\{X_t\}$, all of the random variables X_t have the same probability distribution. In practice, it is often sufficient for a process to be *weakly stationary*, in which the means and variances of the X_t are constant, and the covariances among X_{t_i} and X_{t_j} only depend on the time shift, $s = t_i - t_j$, and not on t_i. However, real-world phenomena often comprise both deterministic and stochastic components. A deterministic component implies a mean value that changes with time which violates (weak) stationarity. Such processes can be (weakly) *trend-stationary*, though, if the deterministic process (trend) can be modeled and fit, leaving residuals which are a (weakly) stationary process. A process $\{Z_t\}$ is trend-stationary if $Z_t = f(t) + X_t$ for any function f and a stationary stochastic process $\{X_t\}$ [26]. De-trending procedures can yield time series that are more amenable to statistical analyses that interpret fluctuations [27, 28]. Experimentally, trend-stationarity in cells corresponds to collecting observations during a sufficiently short time period such that the interaction networks relevant to the dynamics of proteins x and y have not changed.

An obvious advantage of time series data comes from the notion that causality can only propagate forward in time since events in the future cannot affect events in the past. Time series data therefore constrain the possible causal relationships such that, for a given time t we need only consider the effect(s) of $\{X_t\}$ on $\{Y_s\}$ for $s > t$ and vice versa. However, detection of dependence among stochastic processes becomes more complex relative to the case of random variables due to the possibilities of non-stationarity, discussed above, and auto-correlations. To understand these complications, we first generalize the definitions of independence and Pearson's correlation coefficient to the case of stochastic processes. Two stochastic processes $\{X_t\}$ and $\{Y_t\}$ are independent if

$$P(X_{t_i}, \ldots, X_{t_j}, Y_{t_i}, \ldots, Y_{t_j}) = P(X_{t_i}, \ldots, X_{t_j})P(Y_{t_i}, \ldots, Y_{t_j}) \quad (2)$$

for all times $t_i, \ldots, t_j$. This condition is evidently a generalization of the previous definition of independence to all subsets of the two stochastic processes in time. Similarly, if this condition is violated, the stochastic processes are dependent. We also need to generalize the concept of correlation to apply to observations through time. We can calculate a time-dependent Pearson's correlation coefficient by computing the previously defined correlation coefficient as a function of time, i.e.

$$\rho_{\{X_t\},\{Y_t\}}(t_i, t_j) = \frac{\mathrm{Cov}(X_{t_i}, Y_{t_j})}{\sigma_{X_{t_i}}\sigma_{Y_{t_j}}} \quad (3)$$

This value is called the *cross-correlation* and quantifies correlations among the processes $\{X_t\}$ and $\{Y_t\}$ for all times t_i and t_j. For our protein expression example, the cross-correlation quantifies correlations among the sets of observations $\{x_i^{t_i}\}$ and $\{y_i^{t_j}\}$ for all cells $i = 1, \ldots, N$ and times t_i, t_j. If the two processes are not *jointly stationary*, then the cross-correlation depends on all times t_i and t_j, and there is not much predictive power gained from its calculation. However, if the processes are *jointly weakly stationary*, then the cross-correlation no longer depends on the time t_i but only on the time shift $s = t_i - t_j$. Its calculation reveals predictable correlations, or the lack of them, between two processes. We can also consider the correlations of a stochastic process with itself at different times, called the *auto-correlation.* The auto-correlation is defined as the cross-correlation with $\{Y_t\}$ replaced by $\{X_t\}$ and was used previously in the definition of weak stationarity. In many contexts, a time series will have non-zero auto-correlation due to deterministic components, for example. From our previous understanding of correlation and dependence, we know that if a stochastic process $\{X_t\}$ has non-zero auto-correlation at times t_i and t_j, then its constituent random variables X_{t_i} and X_{t_j} are dependent implying that the X_t are not IID. This can complicate quantification of dependence *among* two auto-correlated time series for the following reasons. If the two auto-correlations are either both positive or both negative over the same time range, there is a danger of detecting spurious cross-correlations when in fact there are none. The intuition for this phenomenon is that positive auto-correlation implies a persistence of values over a period of time. This persistence increases the likelihood that two independent but both positively auto-correlated processes

will co-vary during some range of time $t_i, \ldots, t_j$ by chance, and thus appear to be cross-correlated [29]. Surprisingly, a similar phenomenon occurs for two negatively auto-correlated time series [29]. A detailed examination of this phenomenon and modified significance limits on the sample cross-correlation for auto-correlated time series can be found in [29]. There are several techniques that have been developed to correct for the influence of auto-correlation and/or non-stationarity in the calculation of cross-correlation [15, 29] and generalized cross-correlations like power-law cross-correlation [30, 31].

Evidently, determining dependence among processes using time series data can be complex and subject to uncertainty. However, with proper caution, application of the above techniques may answer whether or not two processes belong to a common interaction network. Next, we discuss techniques which aspire to make deeper statements about the nature of interactions such as their strengths, directions, and quantitative descriptions and which require more or less strict assumptions about stationarity and auto-correlation.

3 Undirected network inference

A natural framework for understanding interaction networks comes from the field of network, or graph theory. In this paradigm, variables are represented as nodes and interactions among variables are encoded as edges which connect nodes. Commonly, for a graph with n nodes, the connectivity is represented by the $n \times n$ adjacency matrix A_{ij}, whose entries encode the edges. Edges can either be directed, which implies a causal relationship, or undirected which implies one of several possible causal relationships. This section is concerned with inference of undirected edges (**Fig 1SB**) which are represented by a symmetric A_{ij}. Although undirected networks make less precise statements about interactions, they are typically easier to compute and rely on fewer assumptions than causal inference methods.

3.1 Network inference for data collected at a single time point

We begin by considering inference of networks from data collected at single points in time. Example observations include RNA-seq and proteomics data, and the task is to reconstruct the underlying connections among genes, or gene regulatory networks (GRNs), which explain the observed levels of RNA or protein. The simplest approach is to calculate correlation coefficients among all pairs of variables. This technique is computationally inexpensive and may provide some rough outline of interactors, but it is subject to all of the drawbacks of correlation coefficients discussed in the previous section. Specifically, one can only infer *effective* interactions among variables that share linear dependence, which can lead to many false positives with respect to *direct* interactions in the case of common causes, and false negatives in the case of non-linear dependence. We will discuss examples of solutions to both of these issues.

To detect non-linear dependence, one can instead calculate the mutual information [32] among all pairs. Pairwise calculation of the mutual information among all variables is not subject to false negatives since it accounts for all statistical dependencies, but it still only gives effective interactions which need to be "trimmed" to reveal the true interaction network.

The ARACNE algorithm, designed to infer gene regulatory networks from microarray expression profiles, introduces a possible solution for isolating direct interactions [33]. First, the mutual information between all pairs is calculated, resulting in an over-connected network. Next, a significance threshold on the information I_0 and its corresponding p-value are calculated by randomly permuting the genes and re-calculating mutual information for pairs which should now be overwhelmingly statistically independent. Connections in the original network with a mutual information below I_0 are removed since they are likely statistically indistinguishable from 0, implying independence of the variables. However, the issue of common causes still persists. To address this, the authors use the data processing inequality (DPI) as applied to the possibility of effective interactions. If we assume that nodes X and Y are both directly connected to node Z but not to each other, than the DPI states [33]:

$$I(X, Y) \leq \min(I(X, Z), I(Y, Z)) \tag{4}$$

In words, the smallest mutual information will correspond to the spurious interaction between X and Y. ARACNE thus examines all connected gene triplets and removes the edge with the smallest value [33]. The danger of false positives is greatly reduced, and the authors showed that ARACNE can exactly reconstruct networks comprised of only pairwise interactions and without 3-node loops (although higher-order loops are possible) if mutual information can be estimated exactly [33]. The authors used their algorithm on a B lymphocyte expression profile dataset, successfully separating many direct and indirect interactions previously identified biochemically [33].

The PIDC algorithm [34] offers an alternative way to eliminate effective interactions among genes during GRN inference by using partial information decomposition (PID) [35] to measure multivariate information (MVI) among triplets of genes. The idea behind PID is that the information provided by two source variables X and Z about a third target variable Y can be partitioned into useful categories that help to distinguish dependence structures [34, 35]. The most relevant PID metric for GRN inference in [34] was found to be the *unique* information. The unique information, $\text{Unique}_Z(X, Y)$, quantifies how much information about the target variable Y is provided only by the source variable X given another source variable Z, and is thus not symmetric with respect to a swap of X and Y [34]. The authors found that the ratio of the unique information between X and Y relative to a third variable Z and the mutual information between X and Y

is highest when a direct link exists between X and Y [34]. Using this observation, they propose a strategy in which this ratio is computed for all pairs X and Y relative to all other possible third variables Z which they called the proportional unique contribution (PUC):

$$u_{X,Y} = \sum_{Z \in S \setminus \{X,Y\}} \frac{\text{Unique}_Z(X,Y)}{I(X,Y)} + \frac{\text{Unique}_Z(Y,X)}{I(X,Y)} \tag{5}$$

Last, they compute the confidence of an edge between X and Y using the sum of the cumulative distributions of all PUC scores involving X and Y, which accounts for variations in the distributions of PUC scores among genes due to expression variability, for example [34]. In a separate study [36], the authors applied PIDC to single-cell RNA seq data collected from mouse embryonic stem cells during their progression along the neuronal lineage. They inferred GRNs for two groups of cells that each belonged to two consecutive stages of differentiation in the attempt to identify important gene-gene interactions during such transitions [36]. Since they had three developmental stages, they inferred two such transition GRNs, identifying large rearrangements of the networks during development [36]. Although the motivation to study cells in pairs of states was stated to be because this provided sufficient heterogeneity to detect dependencies [36], it is not clear how to interpret the resulting networks as they likely represent mixtures of the GRNs before, during, and after the transition between the two developmental stages in each group. Methods which explicitly take into account time series are preferable and are discussed next.

Other studies have used information theory to predict the degree and efficiency of information transduction through developmental and signaling networks. Examples include understanding the positional information encoded by four gap genes during *Drosophila* development [37], the determination that individual TNF signaling pathways can make binary decisions [38], an approach based on conditional mutual information [39], and a theoretical study of which network topologies maximize information transmission [40]. Other techniques for undirected network inference have been developed that rely on Markov random fields [41] which will be discussed shortly. Extensive reviews of network inference and other algorithms in the context of gene regulatory networks can be found in [42, 43].

3.2 Inference for undirected time-varying networks using time series data

In the case of time series data, many other network inference techniques are available. The simplest approach is to regard the observations at each point in time as independent observations and calculate network properties for each individually, using techniques described in the previous section. This results in a collection of networks which can be further analyzed to uncover changes in properties with time. This approach essentially disregards time information during the inference step. However, other approaches incorporate the additional information of time ordering in the inference process. Since causes must precede effects, many techniques infer directional (causal) relationships from time series. A second class of dynamic network inference uses time series to infer undirected edges for networks whose topologies change over time [44] (**Fig S4**). Since this clearly violates the assumption of stationarity, these methods have been designed to specifically relax that assumption. This allows for a deeper understanding of molecular interaction networks in cells over long enough periods of time that the connections change, which often occurs during progression from one cell cycle state to the next [45]. Such approaches allow for a more rigorous understanding of questions about how interaction networks must change to enable changes in cell state. In this section we will discuss some of these approaches for time-varying undirected networks.

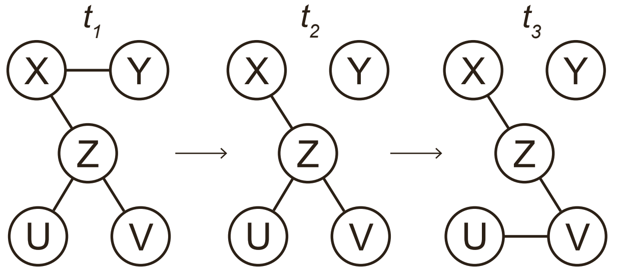

Figure S4: **Time-varying network topology.** Undirected edges between variables in a network can be added or removed as time progresses.

Sparsity. Before we review inference approaches, we first briefly discuss the phenomenon of *sparsity* upon which all of the following approaches rely. A characteristic feature of biological networks, ranging from biochemical to ecological, is that they are sparse [5, 6, 7]. Sparsity means that the real number of connections among nodes is far fewer than the maximum possible number of connections, which implies that many of the entries in the adjacency matrix, A_{ij}, are 0. An important related regression method for understanding sparse linear models is the lasso, first introduced in 1996 [46]. Given the vector of outcomes $\boldsymbol{y}$ and the covariate matrix $\mathbf{X}$, the objective of lasso is to solve

$$\min_{\alpha,\beta} \left\{ ||y - \alpha - \mathbf{X}\beta||_2^2 \right\}, \quad ||\beta||_1 < t \tag{6}$$

where $||\boldsymbol{z}||_p = \left(\sum_{i=1}^N |z_i|^p \right)^{1/p}$ is the L^p norm, and $t \geq 0$ is a tuning parameter [46]. The L^1 norm constraint ensures that many coefficients are equal to 0, satisfying the sparsity condition. A generalization of lasso to inference of network topology came with graphical lasso [47], which applies the L^1 constraint to the inverse covariance matrix and assumes that node values can be modeled as coming from a multivariate Gaussian distribution. The following techniques all depend on graphical lasso and extend it for certain applications.

Markov Random Fields. Another concept upon which these methods rely is the general class of undirected networks called Markov random fields [44, 48, 49]. In such networks, an undirected edge connects two nodes if they are statistically dependent after conditioning on the set of all other nodes in the network [41]. The presence of an undirected edge is then a more precise statement than the statistical dependence discussed in the previous section, but it is still degenerate with respect to the causal relationships it indicates. The degeneracy is actually greater than not knowing the direction of the underlying interaction due to collider bias (**Fig 3**). Conditioning therefore removes spurious links associated with common causes, but introduces new spurious links in the case of common effects. Since undirected edges in Markov random fields correspond to conditional dependence, the methods discussed in this section cannot account for common effects that are only addressed by causal inference methods. For a detailed review of the relationships between Markov random fields and causal models, see [41].

We now discuss several approaches which rely on the sparsity assumption and use Markov random fields to model networks whose topologies change over time. TESLA [50] is an algorithm which infers the topology of time-varying networks over a fixed set of nodes given a time series of node attributes. For networks at each time t, TESLA assumes a Markov random field model. The key component of the method is the estimation of the set of edges at all times at once using an estimator which extends graphical lasso to the case of many graphs. Importantly, their formulation assumes edges do not change much between adjacent time steps and is enforced by an additional L^1 norm constraint on the difference of the values of the same node at adjacent times. The authors applied TESLA to the voting records of senators over 12 periods of 2 months each, identifying connections among conservative Democratic senators and Republicans, for example. They also applied it to a microarray time course dataset of 4000 genes measured at 66 times during development of *D. melanogaster*, but concluded that there were too many missing genes in the dataset to draw major biological conclusions. A very similar algorithm called KELLER was introduced in the same year and also relies on a Markov random field formulation and simultaneous inference for all times to infer time-varying networks [48].

Since both TESLA and KELLER assume that networks change smoothly over time, they are not able to detect sudden or large-scale changes in network topology. A more recent technique, called Time-Varying Graphical Lasso (TVGL), addresses these issues [51]. This algorithm also uses Markov random fields to model networks at each time point. The authors consider several different possible dynamics of the network topology: only a few edges change at a time; large global changes occur; the topology changes smoothly; the topology undergoes blocky restructuring, in which a cluster of nodes changes its topology while the rest of the network is unchanged; or a single node changes all of its connections while the rest of the network remains mostly fixed. For each case, they define a unique penalty function that allows for efficient estimation [51]. For example, if the assumption is that only a few edges change at a time, the same L^1 norm constraint on node value differences from [50] is used. Other norm constraints correspond to the other dynamics, and any prior knowledge about the system can help to choose the most appropriate penalty function and thus lead to the best estimate of the dynamic topology. The authors applied their method to historical stock prices in 2010 for six large companies using the penalty which assumes that a single node can suddenly change its connections. They regarded the stock prices as an indirect readout of the interactions among the companies. They found a large deviation in network topology in the last week of January which was fully attributed to the company Apple. The authors note that on January 27th, Apple introduced the first iPad to the public, and infer connectivity changes among the companies as a result. Their approach is therefore able to detect multiple classes of network topological shifts over time. It is interesting to speculate about which penalties would be most appropriate for different biological settings, and how categories of network topological rearrangements correspond to cellular events. For example, activation of cyclin/CDK networks during cell cycle progression may correspond to blocky restructuring of GRNs [52], while cell differentiation may correspond to large, global rearrangements of GRNs [53]. We reiterate that the undirected links detected by all of these methods can correspond to either a causal interaction between the nodes, or a spurious connection which indicates that the nodes are common causes of a third node (**Fig 3**).

As a final comment, we note that all of the undirected network inference approaches reviewed so far assume that the functional forms of all interactions are pairwise [54] and are graphically represented by edges (**Fig S5A**). However, many real systems exhibit higher-order interactions that are not easily decomposed into pairwise interactions; an enzyme catalyzing an interaction between two other molecules is a simple example of a ternary interaction [55]. All of the approaches described above will necessarily be unable to detect such interactions. Work is currently being done to understand higher-order interactions [55, 56] which are relevant for molecular biology. In general, higher-order interactions among triplets, quadruplets, etc. of nodes are not represented by edges but instead by simplices. A triplet interaction can thus be represented as a triangle which is a 2-simplex, and functions describing interactions must also consider three nodes (**Fig S5B**). Inference techniques are just starting to be developed for higher-order networks and are discussed in [57]. Importantly, there are theoretical results concerning the cases in which higher-order networks reduce to pairwise-interacting networks. One study [58] shows that three-node interactions must be non-linear to cause changes to the network dynamics relative to an equivalent pairwise network.

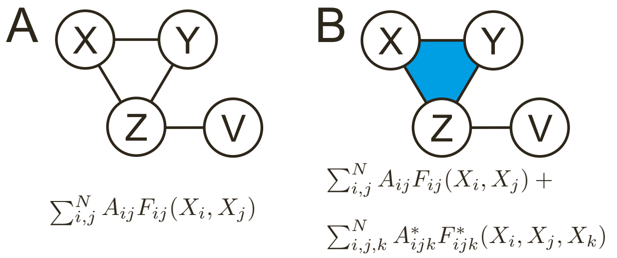

Figure S5: **Higher-order networks.** A) An undirected network whose nodes evolve according to pairwise interactions only is shown. In general, the interactions are described by the product of an adjacency matrix and functions which depend on pairs of variables. B) A higher-order network in which both triplet and pairwise interactions are relevant is shown. A corresponding additional term is required that encodes triplet interactions.

Undirected network inference can be used both for datasets at a single time point and for time series, is amenable to large numbers of variables, can model network topologies that change with time, and soon may be able to robustly infer higher-order interactions. The inference of undirected edges (simplices) between nodes offers more insight than statistical dependence, but still does not definitively establish causality for which inference of directional interactions is required.

4 Dynamical Systems

4.1 Relationships between dynamical systems approaches and causal inference

If all variables in a system are observed, every system of ODEs immediately implies a DAG and therefore a causal structure, if we assume that the variables appearing on the RHS of an ODE comprise all of the direct causes of the variable in question [15]. Of course, a single DAG can imply an infinite number of systems of ODEs, since the functional forms of interactions or even time-dependence of any sort are not specified in a DAG. Work has been done to relate systems of ODEs to the structural causal models (SCMs) introduced in the previous section [59, 60, 61]. Since SCMs do not explicitly account for time, the authors in [59] suggest an approach in which the equilibrium state of the ODEs generates the SCM. They establish a connection between do-Calculus for SCMs and resulting equilibrium states of ODEs after interventions [59]. However, this approach is limited to dynamical systems whose equilbrium states are *fixed point attractors*, to which all trajectories ultimately are drawn regardless of their initial condition. The resulting SCM then discards information about the transient behavior of trajectories on the way to equilibrium. This assumption is obviously very strong and does not encompass the behavior of many dynamical systems. Several more recent approaches have been suggested which construct SCMs from ODEs with time-varying steady states [60] and systems of stochastic differential equations (SDEs) [61]. In the case when there are unobserved variables, the terms appearing on the RHS of an ODE can only be said to be component causes, i.e. they may be either direct or indirect causes. Detection of direct versus indirect causes using dynamical systems approaches is an area of ongoing research that will be important for constructing complete, mechanistic models of cellular interaction networks that describe both causal relationships and dynamics.

4.2 Leveraging dynamical systems theory to learn DAGs

We now review some inference techniques which rely on the framework of dynamical systems as applied to networks, which we refer to as dynamical network inference. The first set of techniques assumes an underlying set of ODEs, but do not seek to explicitly find the functional forms of the interactions and instead leverage the causal assumption of RHS variables as direct causes to infer directed edges. These methods again rely on the assumption that underlying interaction networks are sparse. Interaction dynamics can be represented as a linear system $\boldsymbol{x_i} = \Phi_i \boldsymbol{a_i}$, where $\boldsymbol{x_i}$ is an m-dimensional vector of the state of node i at times $t_1, t_2, \ldots, t_m$, Φ_i is an $m \times n$ matrix encoding the functional form(s) of the interactions between node i and the other $n-1$ nodes at m times, and $\boldsymbol{a_i}$ is an n-dimensional vector which encodes the connectivity of node i with the other $n-1$ nodes. $\boldsymbol{a_i}$ can be understood as the i-th column of the adjacency matrix A_{ij}. The sparsity assumption implies that most of the elements of $\boldsymbol{a_i}$ are 0. According to the compressed sensing theory [62], such linear systems $\boldsymbol{x_i} = \Phi \boldsymbol{a_i}$ can be solved for $\boldsymbol{a_i}$ more effectively by minimizing the L^1 norm rather than the more familiar L^2 norm [62, 63]. The authors in [64] combine the L^1, which ensures sparsity, with an L^2 norm error control term to account for noise in the time series and the possibility of unobserved variables, which is related to the lasso algorithm introduced previously. Specifically, they solve:

$$\min_{\boldsymbol{a_i}} \left\{ \frac{1}{2M} ||\boldsymbol{x_i} - \Phi_i \boldsymbol{a_i}||_2^2 + \lambda ||\boldsymbol{a_i}||_1 \right\} \tag{7}$$

This method performs well on simulated data, but a major drawback is that some knowledge of the functional form of the interactions is required which is a common requirement for dynamic network inference techniques [65, 66].

As a response, "model-free" techniques have been developed which aim to infer network connectivity from time series without any prior knowledge about the underlying dynamics. In [67], the authors employ sets of basis functions to infer connectivity, without requiring an accurate reconstruction of the observed dynamics by the basis functions. The underlying framework considers a set of first order differential equations

$$\frac{dx_i}{dt} = f_i(x_i(t)) + \eta_i(t) \tag{8}$$

where x_i is the state of node i, $f_i : R^n \to R$ is a function which encodes the interactions between node i and the other $n-1$ nodes, and η_i is noise. To isolate whether node j influences node i, the authors introduce the dependency matrices, Λ^i, to the dynamical equations:

$$\frac{dx_i}{dt} = f_i(\Lambda^i x_i(t)) + \eta_i(t) \tag{9}$$

where Λ^i_j is 1 if $\frac{\partial f_i}{\partial x_j} \neq 0$ and 0 otherwise. By further expanding f_i into intrinsic, pairwise, third-order, etc sets of interactions, and by expanding each of those functions into sets of basis functions, the problem formulation is complete. Then, using measured time series and derivatives computed from them, links among nodes can be inferred by regression [67]. If any of the coefficients of the basis functions for a given interaction or set of interactions between nodes is non-zero, then a link is inferred. Of course, this procedure depends on the choice of basis functions, but the authors provide recommendations and evidence that a sufficient set of basis functions can correctly infer connectivity without exactly re-creating dynamics [67].

A final example approaches the problem of distinguishing direct versus indirect interactions using a dynamical systems framework. In [68], the authors infer directed networks under the assumption that interactions can be described as ODEs in which the functional relationships between variables are monotonic. They construct criteria which allow for detection of positive versus negative regulation among variables, and they apply these criteria to surrogate time series in which the time ordering is shuffled to disentangle direct versus indirect interactions [68]. This approach thus incorporates some of the benefits of causal inference, but at the cost of the assumption of monotonic functional forms that describe interactions.

4.3 Automated discovery of differential equations

Alternative approaches automate the discovery of governing equations from time series data. A set of methods called Sparse Identification of Nonlinear Dynamics (SINDy) leverages sparsity and symbolic regression to learn governing equations composed of a given set of basis functions [7, 69]. Similar to the phase portrait approach, derivatives of variables in the system are calculated from time series data and compared with the values of the variables themselves. However, SINDy automates detection of the terms in the resulting system of non-linear ODEs. The assumption is that the time-derivative of the state variables can be written as some combination of a set of basis functions:

$$\dot{X} = \Theta(X)\Xi \tag{10}$$

where $\dot{X}$ are the derivatives computed from the data, $\Theta(X)$ is a basis set of non-linear functions evaluated on the observations X, and Ξ is a vector of coefficients which specifies which basis functions contribute [69]. A reasonable assumption is that the number of terms in the governing equations is much less than the number of basis functions, so solution of the above system can be regarded as a sparse regression problem. Naturally, the authors utilize lasso, or an alternative algorithm tailored for very large datasets to solve this equation [69]. They demonstrate the efficacy of their technique by correctly inferring the equations for the Lorenz system and a mean-field model for vortex shedding using only simulated time series. They also show that SINDy can be extended to correctly infer dynamics and bifurcation diagrams for the 1D logistic map and the 2D Hopf normal form. However, the algorithm fails in the presence of too much noise in the time series measurements. As a response to this issue, SINDy has recently been extended to simultaneously discover governing equations and model noise distributions from time series [70]. The model formulation is altered to reflect that the time series measurements have contributions from both an underlying dynamics and noise, $y(t) = x(t) + n(t)$, where $y(t)$ is the measured time series, $x(t)$ is a theoretical noise-free measurement, and $n(t)$ is noise. Then, the regression problem becomes solution of the system:

$$\dot{Y} = \Theta(X+N)\Xi \tag{11}$$

The solution of such a system is more complex and relies on automatic differentiation [70]. The modified SINDy algorithm is thus able to learn governing equations and noise distributions when the noise is additive. The authors show that this modified SINDy is two times as robust to noise as the original formulation. However, the present formulation cannot account for multiplicative noise which is a common feature of many real-world processes, particularly in biology [71]. In general, optimal selection of basis functions for the SINDy algorithms is an unsolved problem, but inclusion of the proper terms is a pre-requisite for their success [70].

Inference of ODEs that describe relationships among variables allows for mathematically precise descriptions and predictions beyond what is offered by DAGs or SCMs. However, the relationships between the inferred ODEs and underlying causal structures are not clear and will require more research to fully understand. In general, the dynamical systems approach learns effective interactions between variables chosen by the researcher and does not explicitly account for unobserved variables or direct versus indirect causal interactions.

References

[1] Peter Spirtes, Clark Glymour, and Richard Scheines. *Causation, Prediction, and Search.* 2001. DOI: 10.7551/mitpress/1754.001.0001. URL: https://doi.org/10.7551/mitpress/1754.001.0001.

[2] Miguel A. Hernán and James M. Robins. “Instruments for Causal Inference: An Epidemiologist’s Dream?” In: *Epidemiology* 17.4 (2006). ISSN: 1044-3983. URL: https://journals.lww.com/epidem/fulltext/2006/07000/instruments_for_causal_inference__an.4.aspx.

[3] Chun Li and Xiaodan Fan. “On nonparametric conditional independence tests for continuous variables”. In: *WIREs Computational Statistics* 12.3 (2020), e1489. DOI: https://doi.org/10.1002/wics.1489. eprint: https://wires.onlinelibrary.wiley.com/doi/pdf/10.1002/wics.1489. URL: https://wires.onlinelibrary.wiley.com/doi/abs/10.1002/wics.1489.

[4] Kun Zhang et al. “Kernel-based conditional independence test and application in causal discovery”. In: *Proceedings of the Twenty-Seventh Conference on Uncertainty in Artificial Intelligence.* UAI’11. Barcelona, Spain: AUAI Press, 2011, 804–813. ISBN: 9780974903972.

[5] Daniel M. Busiello et al. “Explorability and the origin of network sparsity in living systems”. In: *Scientific Reports* 7.1 (2017), p. 12323. ISSN: 2045-2322. DOI: 10.1038/s41598-017-12521-1. URL: https://doi.org/10.1038/s41598-017-12521-1.

[6] Elias August and Antonis Papachristodoulou. “Efficient, sparse biological network determination”. In: *BMC Systems Biology* 3.1 (2009), p. 25. ISSN: 1752-0509. DOI: 10.1186/1752-0509-3-25. URL: https://doi.org/10.1186/1752-0509-3-25.

[7] Niall M. Mangan et al. “Inferring Biological Networks by Sparse Identification of Nonlinear Dynamics”. In: *IEEE Transactions on Molecular, Biological, and Multi-Scale Communications* 2.1 (2016), pp. 52–63. DOI: 10.1109/TMBMC.2016.2633265.

[8] Patrik Hoyer et al. “Nonlinear causal discovery with additive noise models”. In: *Advances in Neural Information Processing Systems.* Ed. by D. Koller et al. Vol. 21. Curran Associates, Inc., 2008. URL: https://proceedings.neurips.cc/paper_files/paper/2008/file/f7664060cc52bc6f3d620bcedc94a4b6-Paper.pdf.

[9] Shohei Shimizu et al. “A Linear Non-Gaussian Acyclic Model for Causal Discovery”. In: *Journal of Machine Learning Research* 7.72 (2006), pp. 2003–2030. URL: http://jmlr.org/papers/v7/shimizu06a.html.

[10] Judea Pearl. *Causality.* 2nd ed. Cambridge University Press, 2009.

[11] Jean Kaddour et al. *Causal Machine Learning: A Survey and Open Problems.* 2022. arXiv: 2206.15475 [cs.LG].

[12] Dominik Janzig Jonas Peters and Bernhard Schölkopf. *Elements of Causal Inference.* MIT Press, 2017.

[13] Cencan Xing et al. “Regulatory factor identification for nodal genes in zebrafish by causal inference”. In: *Frontiers in Cell and Developmental Biology* 10 (2022). ISSN: 2296-634X. DOI: 10.3389/fcell.2022.1047363. URL: https://www.frontiersin.org/articles/10.3389/fcell.2022.1047363.

[14] Stefan Feuerriegel et al. “Causal machine learning for predicting treatment outcomes”. In: *Nature Medicine* 30.4 (2024), pp. 958–968. ISSN: 1546-170X. DOI: 10.1038/s41591-024-02902-1. URL: https://doi.org/10.1038/s41591-024-02902-1.

[15] Alex Eric Yuan and Wenying Shou. “Data-driven causal analysis of observational biological time series”. In: *eLife* 11 (2022). Ed. by Meredith C Schuman, e72518. ISSN: 2050-084X. DOI: 10.7554/eLife.72518. URL: https://doi.org/10.7554/eLife.72518.

[16] Jakob Runge et al. “Causal inference for time series”. In: *Nature Reviews Earth & Environment* 4.7 (2023), pp. 487–505. ISSN: 2662-138X. DOI: 10.1038/s43017-023-00431-y. URL: https://doi.org/10.1038/s43017-023-00431-y.

[17] Jakob Runge et al. “Detecting and quantifying causal associations in large nonlinear time series datasets”. In: *Science Advances* 5.11 (2019), eaau4996. DOI: 10.1126/sciadv.aau4996. eprint: https://www.science.org/doi/pdf/10.1126/sciadv.aau4996. URL: https://www.science.org/doi/abs/10.1126/sciadv.aau4996.

[18] Andreas Gerhardus and Jakob Runge. “High-recall causal discovery for autocorrelated time series with latent confounders”. In: *Advances in Neural Information Processing Systems.* Ed. by H. Larochelle et al. Vol. 33. Curran Associates, Inc., 2020, pp. 12615–12625. URL: https://proceedings.neurips.cc/paper_files/paper/2020/file/94e70705efae423efda1088614128d0b-Paper.pdf.

[19] Kun Zhang et al. "Causal Discovery from Nonstationary/Heterogeneous Data: Skeleton Estimation and Orientation Determination". In: *Proceedings of the Twenty-Sixth International Joint Conference on Artificial Intelligence, IJCAI-17*. 2017, pp. 1347–1353. DOI: 10.24963/ijcai.2017/187. URL: https://doi.org/10.24963/ijcai.2017/187.

[20] Biwei Huang et al. "Causal Discovery and Forecasting in Nonstationary Environments with State-Space Models". In: *Proceedings of the 36th International Conference on Machine Learning*. Ed. by Kamalika Chaudhuri and Ruslan Salakhutdinov. Vol. 97. Proceedings of Machine Learning Research. PMLR, 2019, pp. 2901–2910. URL: https://proceedings.mlr.press/v97/huang19g.html.

[21] Elena Saggioro et al. "Reconstructing regime-dependent causal relationships from observational time series". In: *Chaos: An Interdisciplinary Journal of Nonlinear Science* 30.11 (2020), p. 113115. ISSN: 1054-1500. DOI: 10.1063/5.0020538. URL: https://doi.org/10.1063/5.0020538.

[22] Thomas Schreiber. "Measuring Information Transfer". In: *Phys. Rev. Lett.* 85 (2 2000), pp. 461–464. DOI: 10.1103/PhysRevLett.85.461. URL: https://link.aps.org/doi/10.1103/PhysRevLett.85.461.

[23] Takuya Imaizumi et al. "Assessing transfer entropy from biochemical data". In: *Physical Review E* 105.3 (2022), p. 034403. DOI: 10.1103/PhysRevE.105.034403. URL: https://link.aps.org/doi/10.1103/PhysRevE.105.034403.

[24] Germán Gómez-Herrero et al. "Assessing Coupling Dynamics from an Ensemble of Time Series". In: *Entropy* 17.4 (2015), pp. 1958–1970. ISSN: 1099-4300. DOI: 10.3390/e17041958. URL: https://www.mdpi.com/1099-4300/17/4/1958.

[25] Patricia Wollstadt et al. "Efficient Transfer Entropy Analysis of Non-Stationary Neural Time Series". In: *PLOS ONE* 9.7 (July 2014), pp. 1–21. DOI: 10.1371/journal.pone.0102833. URL: https://doi.org/10.1371/journal.pone.0102833.

[26] Charles R. Nelson and Charles R. Plosser. "Trends and random walks in macroeconmic time series: Some evidence and implications". In: *Journal of Monetary Economics* 10.2 (1982), pp. 139–162. ISSN: 0304-3932. DOI: https://doi.org/10.1016/0304-3932(82)90012-5. URL: https://www.sciencedirect.com/science/article/pii/0304393282900125.

[27] Mark W. Watson. "Univariate detrending methods with stochastic trends". In: *Journal of Monetary Economics* 18.1 (1986), pp. 49–75. ISSN: 0304-3932. DOI: https://doi.org/10.1016/0304-3932(86)90054-1. URL: https://www.sciencedirect.com/science/article/pii/0304393286900541.

[28] Kung-Sik Chan Jonathan D. Cryer. *Time Series Analysis*. Springer New York, NY, 2010. DOI: https://doi.org/10.1007/978-0-387-75959-3.

[29] Roger T. Dean and William T. M. Dunsmuir. "Dangers and uses of cross-correlation in analyzing time series in perception, performance, movement, and neuroscience: The importance of constructing transfer function autoregressive models". In: *Behavior Research Methods* 48.2 (2016), pp. 783–802. ISSN: 1554-3528. DOI: 10.3758/s13428-015-0611-2. URL: https://doi.org/10.3758/s13428-015-0611-2.

[30] Boris Podobnik and H. Eugene Stanley. "Detrended Cross-Correlation Analysis: A New Method for Analyzing Two Nonstationary Time Series". In: *Phys. Rev. Lett.* 100 (8 2008), p. 084102. DOI: 10.1103/PhysRevLett.100.084102. URL: https://link.aps.org/doi/10.1103/PhysRevLett.100.084102.

[31] Ladislav Kristoufek. "Measuring correlations between non-stationary series with DCCA coefficient". In: *Physica A: Statistical Mechanics and its Applications* 402 (2014), pp. 291–298. ISSN: 0378-4371. DOI: https://doi.org/10.1016/j.physa.2014.01.058. URL: https://www.sciencedirect.com/science/article/pii/S037843711400079X.

[32] Andre Levchenko and Ilya Nemenman. "Cellular noise and information transmission". In: *Current Opinion in Biotechnology* 28 (2014), pp. 156–164. ISSN: 0958-1669. DOI: 10.1016/j.copbio.2014.05.002. URL: https://www.sciencedirect.com/science/article/pii/S0958166914000925.

[33] Adam A. Margolin et al. "ARACNE: An Algorithm for the Reconstruction of Gene Regulatory Networks in a Mammalian Cellular Context". In: *BMC Bioinformatics* 7.1 (2006), S7. ISSN: 1471-2105. DOI: 10.1186/1471-2105-7-S1-S7. URL: https://doi.org/10.1186/1471-2105-7-S1-S7.

[34] Thalia E. Chan, Michael P.H. Stumpf, and Ann C. Babtie. "Gene Regulatory Network Inference from Single-Cell Data Using Multivariate Information Measures". In: *Cell Systems* 5.3 (2017), 251–267.e3. ISSN: 2405-4712. DOI: 10.1016/j.cels.2017.08.014. URL: https://doi.org/10.1016/j.cels.2017.08.014.

[35] P.L. Williams and R.D. Beer. "Nonnegative decomposition of multivariate information". In: *arXiv* (2010). DOI: 10.48550/arXiv.1004.2515.

[36] Patrick S. Stumpf et al. "Stem Cell Differentiation as a Non-Markov Stochastic Process". In: *Cell Systems* 5.3 (2017), 268–282.e7. ISSN: 2405-4712. DOI: 10.1016/j.cels.2017.08.009. URL: https://doi.org/10.1016/j.cels.2017.08.009.

[37] Julien O. Dubuis et al. "Positional information, in bits". In: *Proceedings of the National Academy of Sciences* 110.41 (2013), pp. 16301–16308. DOI: 10.1073/pnas.1315642110. eprint: https://www.pnas.org/doi/pdf/10.1073/pnas.1315642110. URL: https://www.pnas.org/doi/abs/10.1073/pnas.1315642110.

[38] Raymond Cheong et al. "Information Transduction Capacity of Noisy Biochemical Signaling Networks". In: *Science* 334.6054 (2011), pp. 354–358. DOI: 10.1126/science.1204553. URL: https://doi.org/10.1126/science.1204553.

[39] Rosa Aghdam et al. "CN: a consensus algorithm for inferring gene regulatory networks using the SORDER algorithm and conditional mutual information test". In: *Mol. BioSyst.* 11 (3 2015), pp. 942–949. DOI: 10.1039/C4MB00413B. URL: http://dx.doi.org/10.1039/C4MB00413B.

[40] Aleksandra M. Walczak, Gašper Tkačik, and William Bialek. "Optimizing information flow in small genetic networks. II. Feed-forward interactions". In: *Physical Review E* 81.4 (2010), p. 041905. DOI: 10.1103/PhysRevE.81.041905. URL: https://link.aps.org/doi/10.1103/PhysRevE.81.041905.

[41] Laura F. Bringmann Oisín Ryan and Noémi K. Schuurman. "The Challenge of Generating Causal Hypotheses Using Network Models". In: *Structural Equation Modeling: A Multidisciplinary Journal* 29.6 (2022), pp. 953–970. DOI: 10.1080/10705511.2022.2056039. eprint: https://doi.org/10.1080/10705511.2022.2056039. URL: https://doi.org/10.1080/10705511.2022.2056039.

[42] Vân Anh Huynh-Thu and Guido Sanguinetti. "Gene Regulatory Network Inference: An Introductory Survey". In: *Gene Regulatory Networks: Methods and Protocols*. Ed. by Guido Sanguinetti and Vân Anh Huynh-Thu. New York, NY: Springer New York, 2019, pp. 1–23. ISBN: 978-1-4939-8882-2. DOI: 10.1007/978-1-4939-8882-2_1. URL: https://doi.org/10.1007/978-1-4939-8882-2_1.

[43] Michael M Saint-Antoine and Abhyudai Singh. "Network inference in systems biology: recent developments, challenges, and applications". In: *Current Opinion in Biotechnology* 63 (2020). Nanobiotechnology Systems Biology, pp. 89–98. ISSN: 0958-1669. DOI: https://doi.org/10.1016/j.copbio.2019.12.002. URL: https://www.sciencedirect.com/science/article/pii/S0958166919301399.

[44] Yongsoo Kim et al. "Inference of dynamic networks using time-course data". In: *Briefings in Bioinformatics* 15.2 (2014), pp. 212–228. ISSN: 1467-5463. DOI: 10.1093/bib/bbt028. URL: https://doi.org/10.1093/bib/bbt028.

[45] Nicholas M. Luscombe et al. "Genomic analysis of regulatory network dynamics reveals large topological changes". In: *Nature* 431.7006 (2004), pp. 308–312. ISSN: 1476-4687. DOI: 10.1038/nature02782. URL: https://doi.org/10.1038/nature02782.

[46] Robert Tibshirani. "Regression Shrinkage and Selection via the Lasso". In: *Journal of the Royal Statistical Society. Series B (Methodological)* 58.1 (1996), pp. 267–288. ISSN: 00359246. URL: http://www.jstor.org/stable/2346178 (visited on 04/30/2024).

[47] Jerome Friedman, Trevor Hastie, and Robert Tibshirani. "Sparse inverse covariance estimation with the graphical lasso". In: *Biostatistics* 9.3 (2008), pp. 432–441. ISSN: 1465-4644. DOI: 10.1093/biostatistics/kxm045. URL: https://doi.org/10.1093/biostatistics/kxm045.

[48] Le Song, Mladen Kolar, and Eric P. Xing. "KELLER: estimating time-varying interactions between genes". In: *Bioinformatics* 25.12 (2009), pp. i128–i136. ISSN: 1367-4803. DOI: 10.1093/bioinformatics/btp192. URL: https://doi.org/10.1093/bioinformatics/btp192.

[49] Michael Banf and Seung Y. Rhee. "Enhancing gene regulatory network inference through data integration with markov random fields". In: *Scientific Reports* 7.1 (2017), p. 41174. ISSN: 2045-2322. DOI: 10.1038/srep41174. URL: https://doi.org/10.1038/srep41174.

[50] Amr Ahmed and Eric P. Xing. "Recovering time-varying networks of dependencies in social and biological studies". In: *Proceedings of the National Academy of Sciences* 106.29 (2009), pp. 11878–11883. DOI: 10.1073/pnas.0901910106. eprint: https://www.pnas.org/doi/pdf/10.1073/pnas.0901910106. URL: https://www.pnas.org/doi/abs/10.1073/pnas.0901910106.

[51] David Hallac et al. "Network Inference via the Time-Varying Graphical Lasso". In: *Proceedings of the 23rd ACM SIGKDD International Conference on Knowledge Discovery and Data Mining*. KDD '17. Halifax, NS, Canada: Association for Computing Machinery, 2017, 205–213. ISBN: 9781450348874. DOI: 10.1145/3097983.3098037. URL: https://doi.org/10.1145/3097983.3098037.

[52] Robert P. Fisher. "The CDK Network: Linking Cycles of Cell Division and Gene Expression". In: *Genes & Cancer* 3.11-12 (2012). PMID: 23634260, pp. 731–738. DOI: 10.1177/1947601912473308. eprint: https://doi.org/10.1177/1947601912473308. URL: https://doi.org/10.1177/1947601912473308.

[53] Shilu Zhang et al. "Inference of cell type-specific gene regulatory networks on cell lineages from single cell omic datasets". In: *Nature Communications* 14.1 (2023), p. 3064. ISSN: 2041-1723. DOI: 10.1038/s41467-023-38637-9. URL: https://doi.org/10.1038/s41467-023-38637-9.

[54] Marc Timme and Jose Casadiego. "Revealing networks from dynamics: an introduction". In: *Journal of Physics A: Mathematical and Theoretical* 47.34 (2014), p. 343001. DOI: 10.1088/1751-8113/47/34/343001. URL: https://dx.doi.org/10.1088/1751-8113/47/34/343001.

[55] Christian Bick et al. "What Are Higher-Order Networks?" In: *SIAM Review* 65.3 (2023), pp. 686–731. DOI: 10.1137/21M1414024. eprint: https://doi.org/10.1137/21M1414024. URL: https://doi.org/10.1137/21M1414024.

[56] Federico Battiston et al. "Networks beyond pairwise interactions: Structure and dynamics". In: *Physics Reports* 874 (2020). Networks beyond pairwise interactions: Structure and dynamics, pp. 1–92. ISSN: 0370-1573. DOI: https://doi.org/10.1016/j.physrep.2020.05.004. URL: https://www.sciencedirect.com/science/article/pii/S0370157320302489.

[57] Sergio Barbarossa and Stefania Sardellitti. "Topological Signal Processing: Making Sense of Data Building on Multiway Relations". In: *IEEE Signal Processing Magazine* 37.6 (2020), pp. 174–183. DOI: 10.1109/MSP.2020.3014067.

[58] Leonie Neuhäuser, Andrew Mellor, and Renaud Lambiotte. "Multibody interactions and nonlinear consensus dynamics on networked systems". In: *Phys. Rev. E* 101 (3 2020), p. 032310. DOI: 10.1103/PhysRevE.101.032310. URL: https://link.aps.org/doi/10.1103/PhysRevE.101.032310.

[59] Joris M. Mooij, Dominik Janzing, and Bernhard Schölkopf. "From ordinary differential equations to structural causal models: the deterministic case". In: *Proceedings of the Twenty-Ninth Conference on Uncertainty in Artificial Intelligence*. UAI'13. Bellevue, WA: AUAI Press, 2013, 440–448.

[60] Paul K. Rubenstein et al. "From Deterministic ODEs to Dynamic Structural Causal Models". In: *Proceedings of the 34th Annual Conference on Uncertainty in Artificial Intelligence (UAI-18)*. 2018, pp. 114–123.

[61] Stephan Bongers, Tineke Blom, and Joris M. Mooij. *Causal Modeling of Dynamical Systems*. 2022. arXiv: 1803.08784 [cs.AI].

[62] D.L. Donoho. "Compressed sensing". In: *IEEE Transactions on Information Theory* 52.4 (2006), pp. 1289–1306. DOI: 10.1109/TIT.2006.871582.

[63] Domenico Napoletani and Timothy D. Sauer. "Reconstructing the topology of sparsely connected dynamical networks". In: *Phys. Rev. E* 77 (2 2008), p. 026103. DOI: 10.1103/PhysRevE.77.026103. URL: https://link.aps.org/doi/10.1103/PhysRevE.77.026103.

[64] Xiao Han et al. "Robust Reconstruction of Complex Networks from Sparse Data". In: *Phys. Rev. Lett.* 114 (2 2015), p. 028701. DOI: 10.1103/PhysRevLett.114.028701. URL: https://link.aps.org/doi/10.1103/PhysRevLett.114.028701.

[65] Tiago P. Peixoto. "Network Reconstruction and Community Detection from Dynamics". In: *Phys. Rev. Lett.* 123 (12 2019), p. 128301. DOI: 10.1103/PhysRevLett.123.128301. URL: https://link.aps.org/doi/10.1103/PhysRevLett.123.128301.

[66] Srinivas Gorur Shandilya and Marc Timme. "Inferring network topology from complex dynamics". In: *New Journal of Physics* 13.1 (2011), p. 013004. DOI: 10.1088/1367-2630/13/1/013004. URL: https://dx.doi.org/10.1088/1367-2630/13/1/013004.

[67] Jose Casadiego et al. "Model-free inference of direct network interactions from nonlinear collective dynamics". In: *Nature Communications* 8.1 (2017), p. 2192. ISSN: 2041-1723. DOI: 10.1038/s41467-017-02288-4. URL: https://doi.org/10.1038/s41467-017-02288-4.

[68] Se Ho Park, Seokmin Ha, and Jae Kyoung Kim. "A general model-based causal inference method overcomes the curse of synchrony and indirect effect". In: *Nature Communications* 14.1 (2023), p. 4287. ISSN: 2041-1723. DOI: 10.1038/s41467-023-39983-4. URL: https://doi.org/10.1038/s41467-023-39983-4.

[69] Steven L. Brunton, Joshua L. Proctor, and J. Nathan Kutz. "Discovering governing equations from data by sparse identification of nonlinear dynamical systems". In: *Proceedings of the National Academy of Sciences* 113.15 (2016), pp. 3932–3937. DOI: 10.1073/pnas.1517384113. eprint: https://www.pnas.org/doi/pdf/10.1073/pnas.1517384113. URL: https://www.pnas.org/doi/abs/10.1073/pnas.1517384113.

[70] Kadierdan Kaheman, Steven L Brunton, and J Nathan Kutz. "Automatic differentiation to simultaneously identify nonlinear dynamics and extract noise probability distributions from data". In: *Machine Learning: Science and Technology* 3.1 (2022), p. 015031. DOI: 10.1088/2632-2153/ac567a. URL: https://dx.doi.org/10.1088/2632-2153/ac567a.

[71] Megan A. Coomer, Lucy Ham, and Michael P.H. Stumpf. "Noise distorts the epigenetic landscape and shapes cell-fate decisions". In: *Cell Systems* 13.1 (2022), 83–102.e6. ISSN: 2405-4712. DOI: https://doi.org/10.1016/j.cels.2021.09.002. URL: https://www.sciencedirect.com/science/article/pii/S2405471221003392.
