# Supplementary material for: Empirical methods that provide physical descriptions of dynamic cellular processes
Source: Biophys J. 2024 Dec 4;124(6):861–75. doi: 10.1016/j.bpj.2024.12.003 (PMC11947468; doi:10.1016/j.bpj.2024.12.003)
Supplement: Document S2. Article plus supporting material [file mmc2.pdf]

Biophysical Journal
Review

Biophysical Society

Empirical methods that provide physical descriptions of dynamic cellular processes

Ian Seim[1,*] and Stephan W. Grill[1,2,3,*]
[1]Max Planck Institute of Molecular Cell Biology and Genetics, Dresden, Germany; [2]Center for Systems Biology Dresden (CSBD), Dresden, Germany; and [3]Cluster of Excellence Physics of Life, TU Dresden, Dresden, Germany

ABSTRACT We review empirical methods that can be used to provide physical descriptions of dynamic cellular processes during development and disease. Our focus will be nonspatial descriptions and the inference of underlying interaction networks including cell-state lineages, gene regulatory networks, and molecular interactions in living cells. Our overarching questions are: How much can we learn from just observing? To what degree is it possible to infer causal and/or precise mathematical relationships from observations? We restrict ourselves to data sets arising from only observations, or experiments in which minimal perturbations have taken place to facilitate observation of the systems as they naturally occur. We discuss analysis perspectives in order from those offering the least descriptive power but requiring the least assumptions such as statistical associations. We end with those that are most descriptive, but require stricter assumptions and more previous knowledge of the systems such as causal inference and dynamical systems approaches. We hope to provide and encourage the use of a wide array of options for quantitative cell biologists to learn as much as possible from their observations at all stages of understanding of their system of interest. Finally, we provide our own recipe of how to empirically determine quantitative relationships and growth laws from live-cell microscopy data, the resultant predictions of which can then be verified with perturbation experiments. We also include an extended supplement that describes further inference algorithms and theory for the interested reader.

SIGNIFICANCE A major goal of cell and developmental biology is to provide quantitative, mechanistic descriptions of living cells and organisms. Perturbations such as gene knockouts have successfully been used to infer functions of molecules at the cell or organism scale. However, cells have evolved to be robust, and as a result, strong perturbations can give rise to compensation phenomena including restructuring of underlying molecular interaction networks. The interpretation of results is challenging in such cases. In this article, we review inference approaches that rely only on observational data of cells and organisms with interaction networks subject to minimal perturbations, ideally as they naturally occur. We present a range of perspectives and techniques, including statistical relationships, causal inference, and dynamical systems approaches.

INTRODUCTION

The success of biological perturbations and the need for observational studies

In the endeavor to understand molecular cell biology, scientists have leveraged perturbations of living cells to observe their effects and infer their underlying structures. Genetic perturbations are among the most widely used and successful examples of these approaches. The earliest studies used chemicals or radiation to randomly mutate DNA and screened resulting organisms based on phenotypes to infer gene functions. Perhaps the first example of such an approach came in 1927 with Hermann Muller who discovered that x rays mutate DNA in *Drosophila* (1,2). A major advance came with the advent of site-directed mutagenesis techniques in the 1970s, in which researchers could target specific genes (3–5). Since then there have been astounding advances in the ability to precisely and easily modify genomes of living organisms. Perhaps most notably, the understanding in 2012 and 2013 that the bacterial/archaeal viral defense system, clustered regularly interspaced short palindromic repeats (CRISPR), could be used broadly as a gene editing tool has transformed biological research (6,7). Another important genetic manipulation tool with historical ties to antiviral responses is RNA interference (RNAi) (8,9),

Submitted June 21, 2024, and accepted for publication December 2, 2024.
*Correspondence: seim@mpi-cbg.de or grill@mpi-cbg.de
Editor: Meyer Jackson.
https://doi.org/10.1016/j.bpj.2024.12.003
© 2024 The Author(s). Published by Elsevier Inc. on behalf of Biophysical Society.
This is an open access article under the CC BY license (http://creativecommons.org/licenses/by/4.0/).

first definitively demonstrated in *Caenorhabditis elegans* in 1998 (10). RNAi limits or eliminates gene expression via mRNA degradation and can therefore be used as a titration of the effects of a gene of interest (11–13). Altogether, gene knockouts, knockdowns, and specific mutations allow researchers to remove or alter genes and their expression to determine their effects on cell biology and development.

Although these tools and others have been important in uncovering the components of molecular pathways and associations with cellular physiology, we argue that observational approaches can be useful for obtaining a quantitative and complete understanding of cellular systems. In what follows, we distinguish between observational and perturbative studies. Observing a particular biological process often requires the use of transgenic reporters, and we here consider the addition of reporter molecules that only minimally impact the system's dynamics as essential components of a nonperturbative observational study. However, experiments in which molecules have been knocked down, removed, or mutated (other than tagged) to change the underlying interaction networks will be considered perturbations.

Our focus on observational studies is motivated by several concepts. First, observational studies are simpler and can often provide a wealth of data. Second, knockdown and knockout experiments help to make hypotheses about the components of interaction networks and correlations among them, but in general they are not sufficient to describe the quantitative nature of these relationships. Third, perturbations always entail the risk of altering the interaction networks since cells can compensate with rewired or redundant networks (14–16). However, predictions from observational studies must ideally be checked with targeted perturbations in which the results can be understood within their natural context. Therefore, both approaches are necessary for unraveling the complex spatiotemporal processes that underlie living systems. We first provide an example of successful observational inference in the history of the discovery of gravity.

The inference of gravity from observations

The perhaps most striking example of scientific inference using only observational data can be found in the history of the discovery of gravity. The story begins when, as a boy, Tycho Brahe saw a solar eclipse and decided to dedicate his life to astronomy (17). In 1571 he constructed his own observatory where he began a project that resulted in the most accurate measurements that had ever been collected of the positions of the Sun, the Moon, the Earth, and the five other known planets. Tycho also dabbled in theory and proposed a geocentric model of the universe due to his view that the Earth was too heavy to move much (18), which gave equivalent predictions about planetary positions as the heliocentric Copernican model upon a coordinate transformation (19). As neither theory provided a mechanistic explanation for the motions, neither could be ruled out (19).

In 1600, Tycho met Johannes Kepler in Prague, and Kepler became his assistant. During their one year working together before Tycho's death, Tycho refused to share his observations with Kepler, who stole them afterward (17). With this world-class data, Kepler sought to improve upon Tycho's model (20). As the geocentric view was popular at the time, Kepler made his money as a court astrologer and worked on his theories on his own time (17). Ultimately, Kepler used observations of the distance of planets from the Sun along with measurements of the heliocentric longitude to show that orbits were not circular (as stated in the models of both Tycho and Copernicus), but instead were ovals (21). He also noted that the speeds of the planets were inversely proportional to their distance from the Sun, a pivotal example of empirical data analysis. From this, he proposed the existence of an "invisible solar force" pushing planets along and dying off with distance (20). He also proposed a similar force emanating from the Earth, which affects the Moon (22). Combining these observations and inferences, he showed that the orbits in fact had to be elliptical, in addition to his other two laws.

Although Kepler's model was not immediately widely adopted, astronomers came to realize that his framework gave the most accurate predictions of the planetary positions of any existing model (20). Seventy-five years later, Isaac Newton published his theory of universal gravitation, in which he derived Kepler's laws as a consequence of the more fundamental gravitational force and unified celestial and earthly phenomena (21). In this step, Kepler's hypothesized invisible solar force was revealed quantitatively, and classical mechanics and the Industrial Revolution soon followed. Then, 240 years later, Einstein proposed a yet deeper understanding of gravity in his theory of general relativity, in which gravity is a consequence of the curvature of space time (23). The theory was supported to a definite degree by careful observations of gravitational lensing of starlight, predicted by general relativity and visible during a solar eclipse in 1919 (24). The theory of general relativity has led to predictions well beyond the original scope envisioned by Einstein, including black holes and gravitational waves, which have since been experimentally observed (25).

Together, this almost 300-year-long story of discovery provides a beautiful example of careful analysis of precise observations yielding the very deepest of insights. Importantly, these insights came about without the necessity to perform a perturbation experiment by, for example, removing the sun to investigate the impact on the motions of planets.

Towards a comprehensive understanding of molecular cell biology

Next, we will summarize how some of the perspectives and techniques from observational studies have been or

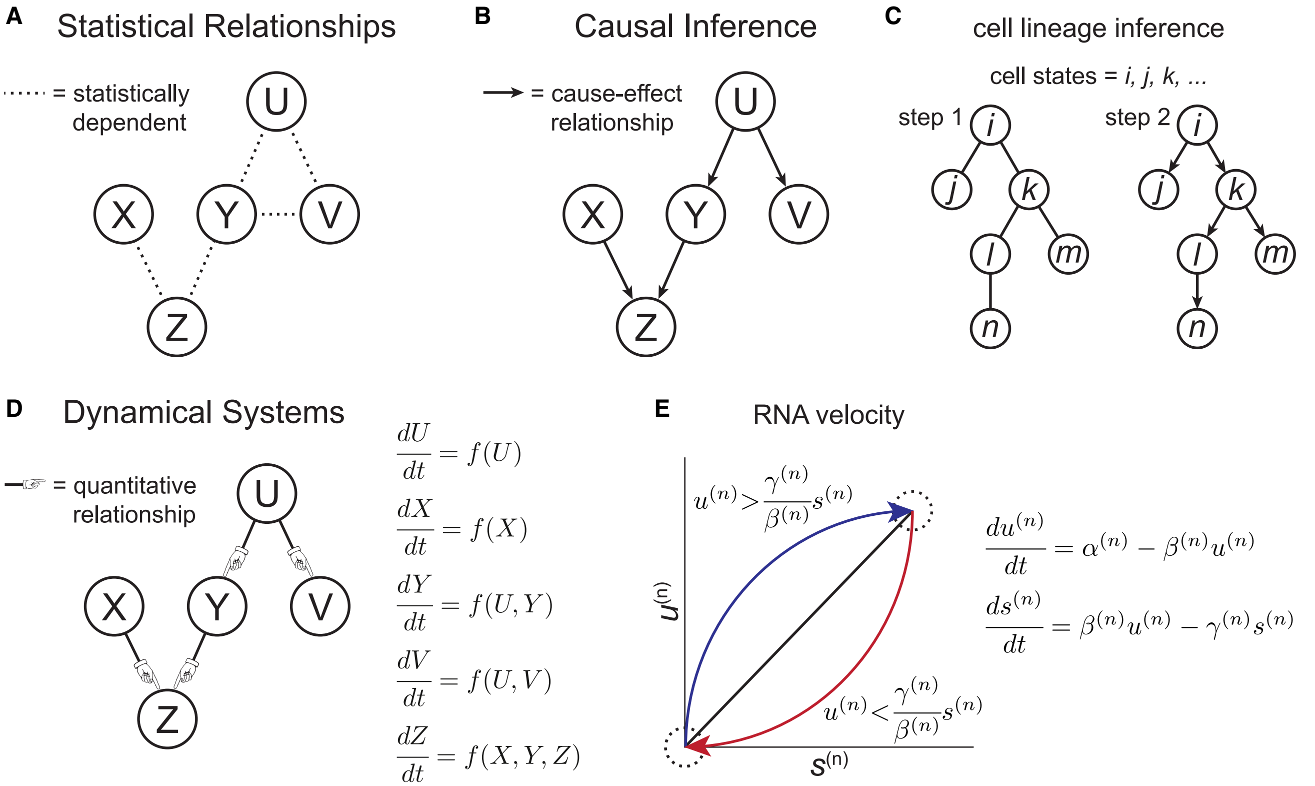

FIGURE 1 Hierarchy of descriptions of interaction networks. (*A*) Statistical dependence between variables is indicated with dashed lines. Variables with a common cause (*Y* and *V*) are statistically dependent. (*B*) Arrows indicate directional causal interactions among variables forming a directed acyclic graph (DAG). (*C*) An example of causal inference in cell lineage inference is shown. In step 1, an undirected network is constructed, and in step 2, the edges are directed to indicate ancestor-descendant relationships. (*D*) Variables and their dynamics are represented as a DAG with an accompanying system of ordinary differential equations in which the dynamics of each variable are determined by its own state and the state of its direct causes. (*E*) The example of RNA velocity is shown, with a phase portrait indicating typical trajectories according to the system of ODEs. The solid black line indicates the steady state corresponding to $u^{(n)} = (\gamma^{(n)}/\beta^{(n)})s^{(n)}$, the dashed circles indicate equilibria for either a constant or 0 transcription rate, and the colored arrows represent typical trajectories during induction (*blue*) and repression (*red*) of transcription. (*E*) Adapted from (31).

may be applied to cell biology. We will focus on the goal of constructing quantitative descriptions of cellular and molecular interaction networks that ultimately give rise to cellular physiology and organismal development. This goal is lofty due to the complexity of cells and developmental processes, which are influenced by the large size of molecular interaction networks (26) and the near certainty of unobserved variables; the presence of noise, including intrinsic noise due to chemical reactions and external noise (27); redundancy in networks in which several molecular interaction networks can achieve the same function (12,14); and nonergodic dynamics in which the topology of interaction networks changes over time (28–30). Despite these challenges, we focus on techniques to gain understanding using an observational approach. Therefore, in this review we focus our thoughts on the following two questions: How much can we learn from just observing? What level of detail about relationships can we obtain from observations? We end with a suggested recipe for scientific inquiry in molecular cell biology using an example in *C. elegans* oocytes: learn as much as possible from observations, make precise predictions, and do targeted perturbations to either confirm or refute those predictions.

INFERENCE FOR DYNAMIC BIOLOGICAL SYSTEMS

We summarize a set of inference techniques that define varying levels of mechanistic detail but are highly interrelated and mutually reinforcing (Fig. 1). First, we review statistical relationships, in which statistical dependence, correlations, and connections to causality are of interest (Fig. 1 *A*).

Next, we discuss causal inference, in which the goal is to go beyond establishing statistical dependencies and to uncover directed interactions that reveal cause-effect relationships (Fig. 1 *B*). An important topic in developmental biology is the inference of cell-state lineages, which are ideally represented as causal graphs that link precursor cells to their descendants and will be a major focus of this section (Fig. 1 *C*). In the case of cell differentiation

during development, an ideal experiment results in a time series of single-cell measurements, such as RNA sequencing profiles and/or morphological descriptors, and connections can then be made between ancestor cells at time t and their descendants at time $t+1$ using similarity measures and without ambiguity in assignment of the direction of connections (32). In cases of development, regeneration, reprogramming, or disease in which detailed time ordering or previous knowledge about lineages is unavailable (33,34), this task is more complex. In general, cell states are first connected into an undirected network using statistical similarity measures applied to expression profiles or morphological features (33) (Fig. 1 *C*). Next, directionality must be assigned to these edges to establish ancestor-descendent (cause-effect) relationships (Fig. 1 *C*). A number of techniques have been recently developed to accomplish this task, which include the use of classical causal inference techniques (35), pseudotime ordering, which relies on random-walk methods (36), and RNA velocity inference (33), although each technique has drawbacks and requires caution since time rates of change are not directly computed but rather inferred. We end this section with a review of causal inference of transcription factor (TF) and target gene interactions, which leverages an inferred cell lineage graph (37). Finally, we also discuss causal inference using light microscopy data, including a study of causal relationships among cell-cycle states and mechanisms of cell division in *E. coli* (38).

We conclude with a discussion of inference techniques that rely on dynamical systems approaches. These frameworks assume that the underlying dynamics can be expressed as a system of ordinary differential equations (ODEs), with states of the system evolving according to the ODEs along trajectories in a state space spanned by the variables of interest (Fig. 1 *D*). The ODEs can be represented by a vector field that is annotated by typical steady states, dynamics, and trajectories, altogether called a phase portrait (Fig. 1 *E*). We review several examples of inferred phase portraits in this section, but here we introduce the RNA velocity technique as a simple example that does not require time series data (Fig. 1 *E*). RNA velocity assumes simple relationships between the abundances of spliced and unspliced mRNA in cells to infer the time derivative of the gene expression state from single-cell RNA sequencing (scRNA-seq) data sets (31). Spliced and unspliced RNAs are distinguished by genomic alignment, with reads aligning to intronic references assigned to unspliced RNAs, and reads aligning to exon-exon splice junctions assigned to spliced RNAs (39). The fundamental idea behind RNA velocity is that the dynamics of spliced and unspliced mRNA abundances for a given gene can be represented by a simple set of ODEs:

$$\frac{du^{(n)}}{dt} = \alpha^{(n)} - \beta^{(n)}u^{(n)}, \frac{ds^{(n)}}{dt} = \beta^{(n)}u^{(n)} - \gamma^{(n)}s^{(n)} \quad (1)$$

where $\alpha^{(n)}$, $\beta^{(n)}$, and $\gamma^{(n)}$ are the transcription, splicing, and degradation rates of gene n, respectively, and $u^{(n)}$ and $s^{(n)}$ are the abundances of unspliced and spliced mRNA corresponding to gene n, respectively. This system defines a dynamical system that is represented by a simple phase portrait (Fig. 1 *E*). Under a constant transcription rate, unspliced mRNA levels first grow, followed by spliced mRNA levels until a steady state is reached at the line $u^{(n)} = (\gamma^{(n)}/\beta^{(n)})s^{(n)}$ (Fig. 1 *E*). If the transcription rate is returned to 0, unspliced mRNA levels first fall, followed by spliced mRNA levels until they both return to 0 (Fig. 1 *E*) (31). The authors of the RNA velocity technique claimed that a large majority of genes obey these simple dynamics (31). Therefore, using measurements of the ratio of unspliced to spliced mRNA levels for gene n across cells, the ratio $\gamma^{(n)}/\beta^{(n)}$ can be estimated by choosing an ensemble of cells where gene expression levels appear to be at steady state, corresponding to cells that populate the upper-right and lower-left corners of the phase portrait (Fig. 1 *E*). Then, for each cell i, the RNA velocity corresponding to gene n can be estimated as the deviation of the observed amounts of unspliced and spliced mRNA from the inferred steady-state amounts, $v_i^{(n)} = u_i^{(n)} - (\gamma^{(n)}/\beta^{(n)})s_i^{(n)}$ (39). The final result is a high-dimensional vector associated to each cell. We review a recent update to the method, which instead uses a chemical master equation (CME) formalism to model bursty transcription and discrete counts of RNA molecules, which is more biophysically realistic (39,40). However, we stress that RNA velocity infers the time rate of change of gene expression in single cells without directly measuring quantities over time. The difficulty of obtaining these direct measurements experimentally has led to ambiguity about the quality of the inferences made by RNA velocity (41), as well as other pseudotime approaches. By comparing RNA velocity estimates with simulated data, Zheng et al. have shown that data preprocessing steps using k-nearest neighbor clustering can strongly distort estimates of both the direction and magnitude of RNA velocity vector estimates (41). Therefore, caution is warranted when interpreting results from RNA velocity inferences. Finally, we conclude with a study that utilized experimental calculation of phase portraits to characterize molecular interactions during cortex activation in the *C. elegans* oocyte (13).

Can you claim any dependents? Statistical preliminaries

The first step in understanding interactions between molecules, cells, or their associated states is to determine whether or not an interaction exists at all. The notions of statistical dependence and correlation are central, although the common phrase *correlation does not imply causation* cautions against overinterpretation. Many familiar statistical concepts are more subtle than is commonly appreciated,

and we begin with a brief summary of some important points.

We focus on uncovering relationships between variables using observational data. Consider two random variables, X and Y. As an example, let X and Y be the concentrations of two proteins in a cell, and let x_i and y_i be simultaneous measurements of the total cellular intensities of the two fluorescently tagged proteins at a single point in time in cell i, normalized by the cell volume. We would like to know which details of the relationship between X and Y are discoverable from the observations. Arguably, the most important property of their relationship is whether or not X and Y are statistically dependent, since this determines if X and Y belong to a common interaction network. Our hypothetical data set consists of N measurements of these two proteins in N cells, each at the same point in time of the cell cycle. To make progress, we assume that these sets of observations are independent and identically distributed, meaning that observations in different cells originate from the same underlying processes but are subject to random variations that arise from intrinsic fluctuations in the cell due to the stochastic nature of chemical reactions and thermal noise, in additional to variations due to experimental error. Then, the collections $\{x_i\}$ and $\{y_i\}$ are *ensembles* that reflect the underlying random variables X and Y, the concentrations of the two proteins. Histograms of $\{x_i\}$ and $\{y_i\}$ will approach the probability distributions of X and Y as $N \rightarrow \infty$.

It is important to note that, despite even the most ambitious experimentalist's efforts, N will always be finite and therefore any inferred information about X and Y will always be approximate. For example, we can estimate the mean of X by computing the sample mean $\overline{X}$ of the observations $\{x_i\}$. The quality of the estimate $\overline{X}$ is given by another estimate, the sample standard error of the mean:

$$\sigma_{\overline{X}} = \frac{\sigma_X}{\sqrt{N}} \tag{2}$$

where σ_X is the sample standard deviation. $\overline{X} \pm 1.96 \times \sigma_{\overline{X}}$ is then an approximation of the 95% confidence interval for the sample mean, which can be interpreted to indicate that out of every 100 realizations of the set of observations $\{x_i\}$, approximately 95 of them will produce confidence intervals around $\overline{X}$ that contain the true mean of X. It is important to note that the error decreases as $\sqrt{N}$, which implies that a decrease by a factor of 10 of the confidence interval requires a factor of 100 more observations. Further, biological systems often exhibit large degrees of variability, which is estimated by σ_X and upon which the confidence interval depends linearly. It is essential to remember that all of the approaches presented in this review must contend with finite samples and are subject to interpretation in light of approximation and error.

Regardless, we continue with the task of determining whether X and Y are dependent. There are several statistics that can be computed from the observations to determine dependence structures. The most familiar is likely the correlation between X and Y. Often, what is meant by the correlation is Pearson's correlation coefficient, $\rho_{X,Y}$, which is defined as their covariance divided by the product of their standard deviations:

$$\rho_{X,Y} \equiv \frac{\mathrm{Cov}(X,Y)}{\sigma_X \sigma_Y} \tag{3}$$

Pearson's correlation coefficient indicates the degree of linear dependence between variables, which is a weaker statement than statistical dependence. Specifically, independence of two variables always implies Pearson's correlation coefficient is 0, but the converse is not true, since the correlation coefficient only detects linear dependence. For example, consider a random variable X that is symmetrically distributed around 0 and let $Y = X^2$. Their correlation is 0 despite a clear dependence of Y on X. Correlation is therefore a poor metric for detecting general dependence structures.

Other quantities have a closer correspondence with statistical dependence. For example, Spearman's rank correlation coefficient is Pearson's correlation coefficient applied to the ranks of the variables instead of their values. It quantifies the extent to which one variable is a monotonic function of the other and is not restricted to linear dependence (42). The mutual information quantifies all dependencies among two variables such that zero mutual information implies independence, and it therefore has greater descriptive power for dependence structures than correlation coefficients (27). Fundamental to the calculation of mutual information is the Shannon entropy, defined by Claude Shannon in 1948 as

$$H(X) \equiv -\sum_{x \in X} p(x)\log(p(x)) \tag{4}$$

where $p(x)$ is the probability distribution of X (43). If we use $\log_2$ in the definition, then the units of H are bits, and the entropy quantifies the expected amount of information gained (in bits) from a single observation of X. The mutual information between X and Y quantifies the amount of information we gain about X by coobserving Y. The mutual information is defined in terms of the Shannon entropy as

$$I(X,Y) \equiv H(X) - H(X|Y) \equiv H(Y) - H(Y|X) \tag{5}$$

where $H(X|Y)$ is the conditional Shannon entropy of X given Y. In practice, since we only have finite data sets $\{x_i\}$ and $\{y_i\}$, we can only compute approximations of and confidence intervals around correlation coefficients and mutual information to infer dependence between X and Y.

If there is evidence of dependence between X and Y, an immediate implication is the existence of some causal structure as stated by Reichenbach's common cause principle: if two random variables X and Y are dependent, then either X causes Y, Y causes X, or some other variable Z causes both (Fig. 2) (44). The terminology "common cause" can be understood if we regard the common cause Z as reducing to either X or

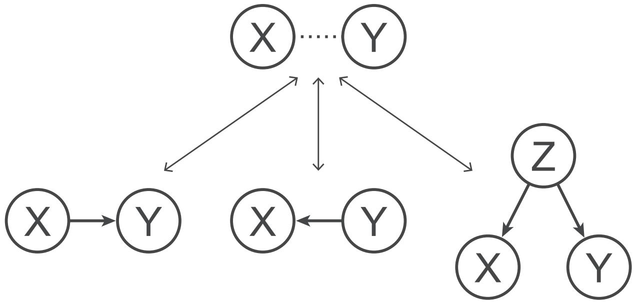

FIGURE 2 The common cause principle. If *X* and *Y* are statistically dependent, as indicated by the dashed line, then either *X* causes *Y*, *Y* causes *X*, or an unobserved variable *Z* causes both.

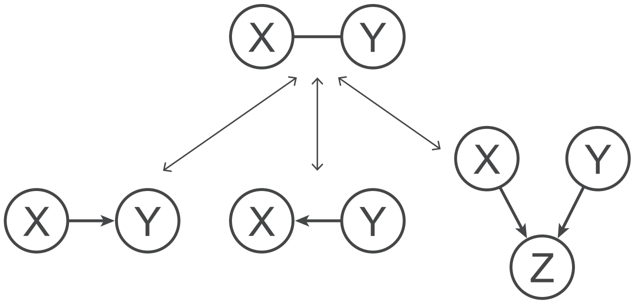

FIGURE 3 Collider bias. If *X* and *Y* are dependent after conditioning on all other variables, indicated by the solid line, then either *X* causes *Y*, *Y* causes *X*, or *X* and *Y* both cause a common effect *Z*.

Y in the case of a direct causal relationship between *X* and *Y*. If *X*, *Y*, and *Z* are all observed, conditional independence tests can be used to narrow down the possible causal structures, and will be discussed in the context of a recent study (38). However, conditional independence tests can introduce a new kind of spurious link, called collider bias. Collider bias states that if *X* and *Y* both cause *Z*, then *X* and *Y* are dependent after conditioning on *Z*. We are thus left with a common effect principle: two variables *X* and *Y* are dependent after conditioning on all other variables if *X* causes *Y*, *Y* causes *X*, or *X* and *Y* both cause a common effect *Z* (Fig. 3). Again, *Z* reduces to *X* or *Y* in the case of a direct causal relationship between *X* and *Y*. Conditioning therefore removes spurious links associated with common causes, but introduces new spurious links in the case of common effects. These spurious links have been cleverly leveraged to constrain causal relationships in causal inference algorithms (45), two of which are discussed in supporting material, section 1: causal inference from observations. How are the common cause and common effect principles related to the phrase correlation does not imply causation? If we take correlation to mean Pearson's correlation coefficient, then the familiar phrase could, perhaps, be more accurately (but less memorably) restated as: correlation implies a common cause, conditional correlation implies a common effect, but the absence of correlation does not imply independence.

For a discussion of auto- and crosscorrelation and stationarity in time series, see supporting material, section 2: correlations and dependence among and within time series. We now review several recent studies that go beyond approximations of pairwise dependencies to infer causal interactions.

BEYOND DEPENDENCE: CAUSAL INFERENCE

A primary goal of scientific research is to identify cause and effect relationships. We represent such relationships using directed, acyclic graphs (DAGs), in which an arrow pointing from *X* to *Z* indicates that *X* causes *Z* (Fig. 1 *B*) (46,47). A causal relationship between *X* and *Z* contains more information than a dependence between *X* and *Z*. While dependence is a symmetric relation, the statement that *X* causes *Z* indicates that a perturbation of *X* will change the probability distribution of *Z*, but not vice versa. Causal inference therefore enables prediction of the effects of perturbations, while knowledge of statistical dependence alone does not.

The ability to establish causality is a contentious topic and has been debated by philosophers and scientists dating back at least to Aristotle (48). Our goal is to discuss the extent to which it is possible to infer causal relationships from observational data in the context of developmental and cellular biology. Due to all of the issues discussed above stemming from statistical dependencies and their obfuscation of causal relationships due to common causes and/or effects, it may seem like an impossible task. Also, since the goal of causal inference is to make predictions about perturbations, the most straightforward approach appears to be perturbation and experiment. It turns out that there are cases in which causal inference is possible from observational data and provides an assessment of cellular systems that does not have the risk of network rewiring entailed by perturbations. We begin by first reviewing some concepts in causality as they relate to cell biology, and proceed with examples of work in the fields of cell-state lineage inference and causal inference from microscopy data.

For a discussion of causal inference theory and algorithms, see supporting material, section 1: Causal inference from observations.

Causal inference in the cell

The cell presents unique challenges with respect to the establishment of causality. We introduce some important concepts that help to clarify the meaning of causes and effects in the context of the cell. The following ideas are discussed in great clarity and more depth in (49,50); we briefly discuss them in the case of molecular biology.

Direct and indirect causes

We distinguish types of causes in terms of their proximity to an effect in a DAG. For the effect *Z*, we say that *Y* is a direct cause of *Z* and *U* is an indirect cause of *Z* (Fig. 1 *B*). In practice, these distinctions depend upon the extent to which we

can actually observe U, Y, and Z (49). For example, if we only observe U and Z, we would infer that U is a direct cause of Z. This is related to the issue of unobserved causes discussed below.

Feedback

There are countless examples of feedback structures in molecular interaction networks. Graphically, these are represented by loops or cycles. Since causal interactions are represented by DAGs, there appears to be a problem with representation of feedback structures. However, introducing time resolves the issue by assigning a node for each variable at each point in time, which requires a time discretization (49). We can then understand feedback interactions as propagating forward through time and create DAGs for arbitrary feedback structures (Fig. 4 *A*).

Unobserved causes

The complexity of molecular interaction networks in cells all but guarantees the presence of unobserved variables that are relevant to any observational study. For example, a phosphorylation event early in a signaling cascade could set off a chain of interactions that ultimately lead to activation of a TF. How then do we understand causality in this context if we only observe the phosphorylation event and the TF activation, inferring a directed edge between the two? It is helpful to understand the concepts of component causes and sufficient causes (50). In general, a sufficient cause of an effect is made up of several component causes which are all necessary to cause the effect. In our example, the set of interactions in the signaling cascade comprise a sufficient cause of the TF activation, and each of the interactions are component causes. Perturbation of any of them would alter the activation of the TF. Therefore, whenever we infer a causal link, there is always the possibility that the link is indirect, and that there are hidden component causes which belong to the same sufficient cause.

Multicausality

Redundancy of molecular interaction networks is a widely appreciated phenomenon that ensures robustness in cell physiology (12,14). Basically, redundancy arises from multiple sets of interaction networks that can give rise to the same outcome. This reality corresponds to the concept of multicausality (50), in which one event can have multiple sufficient causes. Each of the event's sufficient causes in general comprises several component causes, which can be shared among different sufficient causes (50). Therefore, identification of one cause does not in general rule out the possible existence of another cause. Multicausality also poses problems for the interpretation of perturbation experiments, since alteration of a redundant component may lead to the false conclusion of no causal significance when it is in reality compensated for by redundant processes.

The discussion of the above points is not intended to discourage the pursuit of understanding causality in molecular cell biology. Rather, it is to outline the extent to which we can interpret causal inferences and to encourage a broader understanding in which multiple causes and explanations can actually reflect the underlying truth of highly complex and robust systems such as living cells.

Cell-state lineage inference

The goal of cell-state lineage inference is to identify distinct cell states or types and to connect them in a DAG that

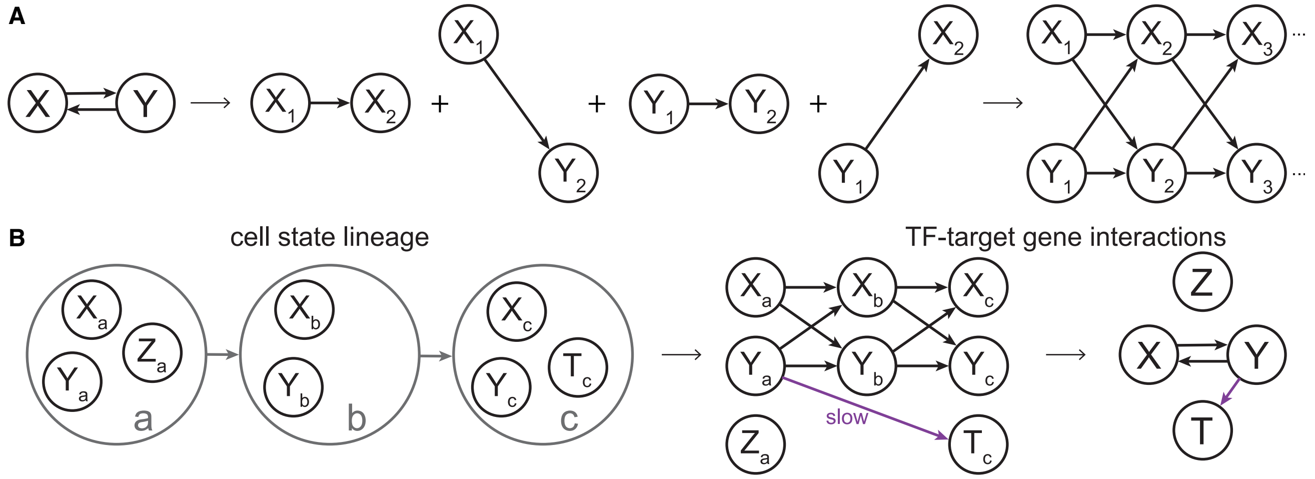

FIGURE 4 DAGs with time indices. (*A*) Without considering time, feedback structures result in cycles (*left*). However, feedback structures can be represented as DAGs if the value of each variable at discrete points in time is represented as a node and causal interactions flow forward in time. In this example, X and Y both cause each other over a time lag of 1. (*B*) TF-target gene interactions can be inferred according to a cell-state lineage (*left*). Cell states are shown as gray circles and indexed *a–c*, with expression of genes in each cell state. An example set of interactions are shown on the right, with genes indexed by cell state, fast interactions shown in black, and slow interactions shown in magenta. (*B*) Adapted from (37).

represents ancestor-descendant relationships (Fig. 1 *C*). Cell states can be defined by genomic data, such as scRNA-seq profiles, and/or by other characteristics such as cell morphology, mechanical responses, morphogen patterns, etc. Here, we discuss several cases which use genomic data, and we briefly discuss a study that generalizes the notion of cell states in the conclusion of the review.

The first study of interest utilized time series of scRNA-seq data that were collected for the well-studied model organism *Xenopus tropicalis* during the first day of life after fertilization (32). The authors obtained RNA expression profiles for all of the cells in *Xenopus* embryos across 10 developmental stages ranging from before the onset of zygotic transcription through the early tail bud stage when dozens of cell types have differentiated (32). After annotating 87 cell types and 259 cell states, their goal was to connect these cell states with directed edges, forming a cell-state lineage. Since their data comprised time series of observations, they limited the possible connections between cell states to point from cells at time t to cells at time $t+1$, avoiding the directionality inference problem by using direct observation (Fig. 5). Therefore, the only remaining task was to determine connections between ancestor and descendant cells. Since a descendant cell state can only arise from a single ancestor cell state, they used similarity measures among expression profiles to assign the most similar cell in time t to cell states in time $t+1$ (Fig. 5). Specifically, they first embedded all cells from adjacent time points t and $t+1$ into the principal component (PC) space arising from the $t+1$ cells. They then computed the Euclidean distance between cell clusters in adjacent time points and inferred a connection for the closest distances (32).

This scheme allowed for a branching architecture of the lineage tree in which branches can form going forward in time and mostly matched known lineage relationships. However, the authors found that several cell types emerged earlier in development than thought previously, including an endothelial/hemangioblast progenitor, tail bud, and several epidermal cell types (32). They conclude that this finding indicates previously unknown early transcriptional dynamics. In a companion piece (51), some of the authors applied the same technique to zebrafish embryos during development,

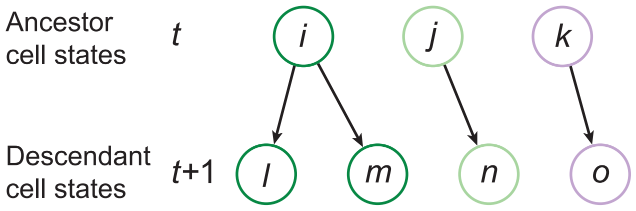

FIGURE 5 Connecting ancestor cell states with descendant cell states. Ancestor cell states at time t are connected to descendant cell states at time $t+1$ according to gene expression similarity, denoted by the colors of the circles. Figure adapted from (32).

which allowed for a comparison of the cell fate lineages between the two species. In both species, they observed a gradual increase in the complexity and combinatorial nature of TF expression that coincided with embryonic cell-state differentiation (32). However, they found large differences in tissue-specific expression of individual genes between the two species and instead found that orthologous cell states shared expression of only a subset of genes that were mostly TFs (32). With such a rich set of cell states connected by ancestor-descendant (causal) links, the authors were able to make wide conclusions about the developmental programs of two species.

Acquiring time series of organism-level scRNA-seq data during multiple developmental stages is challenging and expensive. In addition, we are also interested in systems in which lineages and cell types have not been extensively studied previously, as in regeneration, reprogramming, or disease models. Is it possible to infer such rich graphs from a single snapshot scRNA-seq data set in which time ordering and substantial previous information about the system is not known a priori? Several techniques have been recently developed to facilitate such inference. The first step is always to construct an undirected network that connects cell states based on gene expression similarity. A discussion of undirected network inference can be found in supporting material, section 3: Undirected network inference. Several approaches have been taken to assign directionality to edges. CellRank is a recently developed method that uses RNA velocity information to assign directionality (33). The rationale is that RNA velocity gives information about the likely future state of the gene expression levels in a cell and thus can be used to assign directionality to cell-state transitions. CellRank models cell-state changes as a Markov chain, in which each state is defined by a scRNA-seq profile, and transitions to descendant states only depend on the most recent ancestor state and are probabilistic. CellRank uses the inferred, single-cell gene expression vectors from RNA velocity to orient the edges detected in the previous step. The assignment is probabilistic, with neighbors who are closest to the direction indicated by the velocity vector given the higher transition probability. This is implemented by calculating the Pearson correlation, c_{ik}, between each velocity vector v_i for cell i, and each connection s_{ik} to neighboring cell k (33). Finally, the set of transition probabilities is defined as:

$$p_{ik} = \frac{e^{\sigma c_{ik}}}{\sum_j e^{\sigma c_{ij}}} \quad (6)$$

where $\sigma = 1/\text{median}(|c_{ik}|)$. The authors note, however, that the RNA velocity vectors are inherently noisy (33), which propagates into their estimates of transition probabilities. To cope with this uncertainty, they compute a weighted average of their transition probability matrix and a similarity-based transition probability matrix (33).

The end result is then a weighted, directed graph connecting cell states, where edge weights correspond to transition probabilities. The authors used CellRank to identify trajectories through delta cell precursor states in murine pancreatic cells, and to predict and confirm that goblet cells dedifferentiate toward basal cells during murine lung regeneration (33).

Gene regulatory network inference on DAGs

Establishment of cell-state lineages is valuable, but we would also like to know the extent of the interactions between certain genes in the context of gene regulatory networks (GRNs). A recently developed method, Velorama, leverages cell lineages to better infer GRNs (37). The primary goal of Velorama is to infer causal TF-target gene interactions. Velorama first requires an inferred cell-state lineage, and the authors report that using the output of CellRank leads to the best results. The algorithm then uses the lineage, a DAG, to temporally order genes with respect to one another and uses Granger causal inference to detect cause-effect relationships between genes (Fig. 4 *B*) (37). Granger causality was originally developed in the context of economics and determines causality among variables X and Y by asking whether the past history of all variables including X improves predictions of the current values of Y, relative to the past histories of all other variables without X (49). If the predictions of Y are better when including the history of X, then X Granger causes Y. Granger causality thus is able to distinguish direct and indirect causes when they are all observed (49). For a system with G variables, variable j is modeled as a function of the previous L observations of the other variables as:

$$\begin{aligned} z_j(t) \; &= f_j\big(z_1(t-L;t-1), z_2(t-L;t-1), \ldots, \\ &\quad z_G(t-L;t-1)\big) + e_j(t) \end{aligned} \tag{7}$$

where $z_j(t)$ is the value of variable j at time t, $z_k(t-L;t-1)$ is the sequence of values of variable k from times $t-L$ to $t-1$, and $e_j(t)$ is an error term (37). The authors of Velorama extend Granger causality from the typical case in which the f_j are linear functions and all variables have a global ordering $t, t+1, \ldots$, to the case of complex TF-target gene interactions on an inferred DAG. They instead model the f_j as multilayer neural networks, and replace a global time index in the observations z_k with ancestor-descendant lineages according to the inferred DAG (37). They are thus able to infer causal relationships between TFs and target genes, in which the set of relevant TFs for a given target gene belong to cell-state ancestors of the given target gene's cell state. Because Velorama considers whole sets of TFs across different lag times (parent, grandparent, etc., cells in the lineage), it is able to account for multicausality (TF cooperativity) and different speeds of TF influence on target genes (37) (Fig. 4 *B*).

The authors used Velorama to study TF-target gene interactions in a data set comprising paired scRNA-seq and chromatin accessibility data of human fetal cortical samples during midgestation (37). They first demonstrated that their inferred speeds of TF action on target genes were verified by the chromatin accessibility data (37). They next found that the fast TFs were mostly specifically expressed in brain cells, while the slow TFs were generally expressed in a broader set of cell types. They also found that fast TFs are more implicated in neuropsychiatric disorders, while the slow TFs are more implicated in the formation of gliomas. They hypothesize that the specific expression patterns of the fast TFs are consistent with neural diseases, while the more general expression of the slow TFs are consistent with more systemic diseases such as cancer (37). Finally, they analyzed the degree of cooperativity among TFs, finding that TFs of different speeds often cooperate. They identified a specific system, which comprises the slow TFs SATB2 and HMGB2, which are chromatin remodelers that cooperate with the fast TFs BCL11B and EOMES, which drive cell fate changes during differentiation (37). Velorama therefore leverages two levels of causal inference, first creating cell-state lineages that restrict the space of possible causal interactions among the second layer of TF-target gene interactions. The identification of causal, cooperative TF-target gene interactions promises to reveal deep insights into gene regulation during development and disease.

Causal inference using microscopy data

We now review a recent study that leveraged causal inference using microscopy data (38). Kar et al. used simple conditional independence tests to infer causal relations among stages of the cell cycle in *Escherichia coli* (38). The research leverages structural causal models (SCMs) (44,52), which naturally follow from DAGs and which we define now. For the example DAG:

$$X \rightarrow Y \rightarrow Z, A \rightarrow Y \tag{8}$$

the corresponding SCM is:

$$\begin{aligned} X \; &= f_X(\eta_X), Y \; = f_Y(X, A, \eta_Y), \\ A \; &= f_A(\eta_A), Z \; = f_X(Y, \eta_Z) \end{aligned} \tag{9}$$

where the η_i are independent noise terms. In an SCM, each variable is a function of its own noise term and its direct causes (44). A new feature of SCMs relative to DAGs are the explicit noise terms, which reflect the reality that each node in the DAG is a random variable. The assumption of independent noise terms implies that all common causes are observed, because if the noises were dependent, their associated variables would have some unobserved common cause. Using specific functional forms of the f_i allows one to make predictions about perturbations of any variable and to choose the most appropriate conditional independence tests for inference.

In (38), the authors observed the growth of *E. coli* in media, which promotes either slow or moderately fast growth, and collected data about the lengths of cells at birth L_b, initiation of DNA replication L_i, and division L_d, as well as during other cell-cycle events. They set out to compare several competing models of cell-cycle regulation that posit distinct causal interactions among these events, resulting in different DAGs. To discriminate among the models, the authors found pairs of variables that should be conditionally (in)dependent given a DAG (Fig. 6) (38). The authors assumed that the functional forms of the SCMs, which are implied by their DAGs are linear and can be interpreted as Taylor expansions around the average nonlinear interactions among cell-cycle events (38). They also assumed that the noise terms are independent and Gaussian. These assumptions allowed them to test for conditional independence simply using linear regression and Pearson correlations of the resulting residuals. For example, to discriminate among a model in which only replication cues control cell division and a model in which both cues at cell birth and replication control cell division, they calculated the conditional correlation $r(L_b, L_d|L_i)$ by obtaining the residuals after linear regression of L_b on L_i and L_d on L_i and then obtaining the Pearson correlation between those residuals (Fig. 6). The presence or absence of a statistically significant correlation then serves as a test for conditional independence of L_b and L_d given L_i and allowed the authors to infer causal relationships among cell-cycle events (38). They found that the model shown in (Fig. 6 *A*) is consistent with observations in slow growth media, while the model shown in (Fig. 6 *B*) is consistent with observations in fast growth media. The authors investigated several other relationships and proposed a few possible molecular mechanisms consistent with their inferred models for future study.

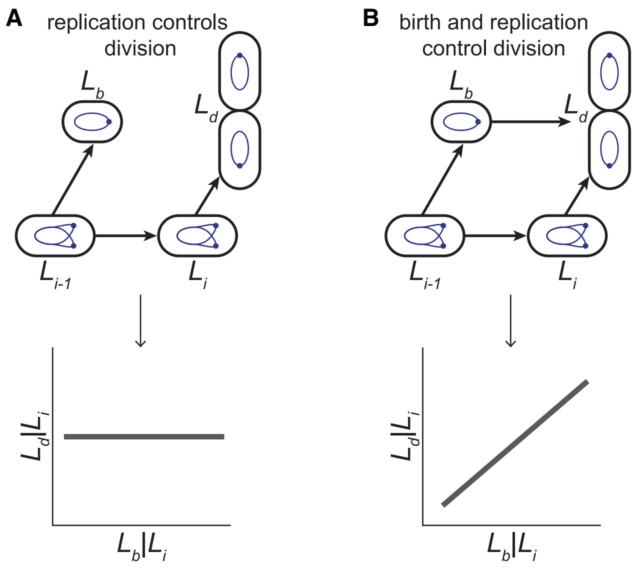

FIGURE 6 Conditional independence tests to distinguish models of *E. coli* cell-cycle control. (*A*) Causal links are shown in the model in which only replication cues control division (*top*). L_{i-1} is the length of cells during the previous replication initiation event, L_i is the length during the current initiation event, L_b is the length at birth, L_d is the length at division. Based on the causal graph, there is no expected correlation between $L_b|L_i$ and $L_d|L_i$. (*B*) The causal graph corresponding to the model in which cues from both birth and replication control division is shown (*top*). For this model, there is a positive expected correlation between $L_b|L_i$ and $L_d|L_i$. Figure adapted from (38).

Causal inference aims to answer questions about directional interactions and can account for the challenges of common causes and spurious interactions. However, in addition to inferring the presence and direction of interactions, we would like to understand the dynamics of the interactions ideally as explicit functions. Although methods have been proposed to quantify the strength of causal links (53), we next focus on an alternative framework that relies on dynamical systems theory and models interaction networks as systems of ODEs.

CELLULAR PHASE SPACE: DYNAMICAL SYSTEMS APPROACHES

This final section reviews techniques that are based on dynamical systems. A dynamical system is a system of coupled ODEs that represents the interactions among a set of variables through time. The framework of dynamical systems allows us to regard the state of a system as a point in a phase space in which each coordinate represents a variable of interest. The ODEs define a velocity vector at each point in phase space, which we can follow from any given initial condition (initial point in phase space) to construct a trajectory (Fig. 7, *B* and *C*). Trajectories can correspond to arbitrary processes that evolve in time, including chemical reactions, patterns of neuron activation, populations of species, etc. There is a long history of regarding biological systems as dynamical systems (54), ranging from descriptions of molecules, to cells, to ecosystems. A benefit of a description that comprises ODEs is that interactions are captured in mathematical form, enabling quantitative predictions. There is also a vast literature related to long-term behaviors of dynamical systems which can either settle into one of a handful of known patterns or display chaotic behavior (54–56). Phase portraits are a particularly useful way of describing behaviors of dynamical systems (Fig. 7, *B* and *C*) and will be discussed in more detail as inference tools shortly (13,54,57). Finally, the evolution of steady states of a given variable with respect to a given parameter in the dynamical system is summarized by bifurcation diagrams (55). Some authors have argued that there is a qualitative correspondence between signal-response curves and bifurcation diagrams (55). Dynamical systems thus provide researchers with a wide array of established mathematical tools and offer the most precise descriptions of interactions discussed thus far. Ultimately, comprehensive descriptions require that the ODEs are placed in space and generalized to systems of partial differential equations

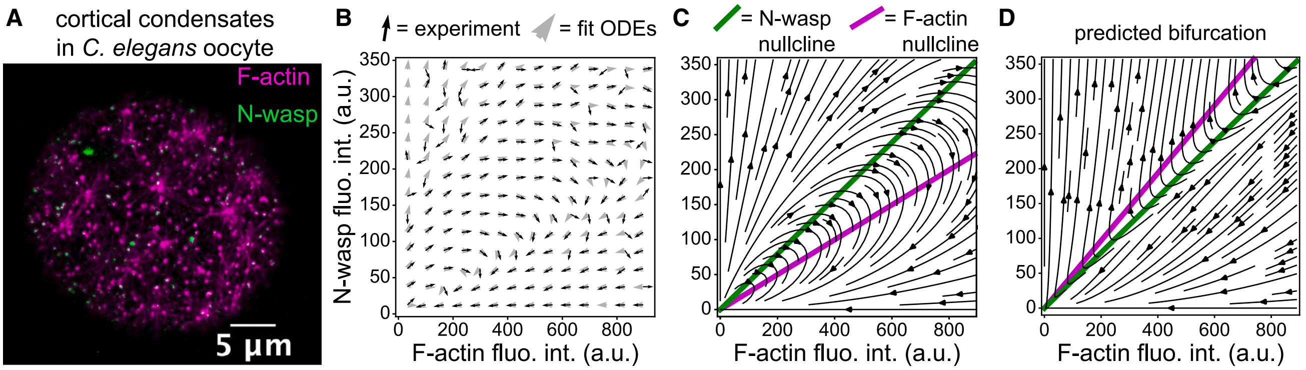

FIGURE 7 Cortical condensates and their phase portraits. (*A*) Cortical condensates are shown in a *C. elegans* oocyte. F-actin is labeled with LifeactmKate2 and N-wasp is labeled with eGFP. (*B*) The experimentally inferred phase portrait is shown by black arrows that represent normalized velocity vectors calculated from experimental time series of N-wasp and F-actin fluorescence intensities in thousands of cortical condensates. The gray arrows are vectors calculated from the fit system of ODEs (13). (*C*) The streamlines and N-wasp and F-actin nullclines are shown for the system of ODEs. The fixed point at (0,0) is a saddle point that is stable in the F-actin direction and unstable in the N-wasp direction. (*D*) The streamlines and N-wasp and F-actin nullclines are shown for the system of ODEs after a bifurcation due to increased k_d.

(PDEs). Spatial inference techniques are outside the scope of this review but will be essential for complete understanding. We therefore restrict our focus to inference that relies on ODEs and provide some references for inference of PDEs and spatial causal inference. We begin with a discussion of recent updates to RNA velocity and end with a study that leveraged experimental inference of a phase portrait to learn the governing equations of F-actin and N-wasp dynamics during cortex activation in *C. elegans* oocytes.

RNA velocity as a discrete Markov process

As introduced previously, RNA velocity depends on estimation of the levels of unspliced and spliced mRNA from scRNA-seq data and makes predictions about their future values by fitting to a simple dynamical system (Fig. 1 *C*). The original approach (31) and an updated approach called scVelo, which allows for dynamic transcription rates (58), both assume smooth, deterministic dynamics of mRNA counts in cells. However, it is well known that mRNAs often have low copy numbers in which discrete descriptions are superior (39). In addition, transcriptional dynamics are known to be bursty, in contrast to the smooth continuous functions posited in the previous examples (39). Therefore, Gorin et al. argue for a discrete, Markov process model which is described by a CME (39). The CME describes the flux of probabilities between the states of variables with integer counts and is an infinite series of coupled ODEs (39). The dynamics given in the original RNA velocity paper can be described as:

$$\varnothing \xrightarrow{\alpha(t)} u \xrightarrow{\beta} s \xrightarrow{\gamma} \varnothing \tag{10}$$

which implies the following CME:

$$\begin{aligned}\frac{dP(u,s;t)}{dt} &= \alpha(t)[P(u-1,s;t) - P(u,s;t)] + \\ &\beta[(u+1)P(u+1,s-1;t) - uP(u,s;t)] + \\ &\gamma[(s-1)P(u,s-1;t) - sP(u,s;t)]\end{aligned} \tag{11}$$

where $\alpha(t)$ is a piecewise constant transcription rate, β is the splicing rate, γ is the degradation rate, and $P(u,s;t)$ is the probability of the state with u unspliced transcripts and s spliced transcripts at time t (39). This model more accurately represents low copy numbers and the intrinsic noise in transcription and splicing reactions (39). To incorporate bursty transcription dynamics, the authors have recently introduced a new model and inference method (40). Their model can be represented as:

$$\varnothing \xrightarrow{\alpha} B \times u \xrightarrow{\beta} s \xrightarrow{\gamma} \varnothing \tag{12}$$

where transcriptional events occur as a Poisson process with rate α, B is the burst size during a transcriptional event and is a geometric distribution with mean b, and β and γ are defined identically as in the previous model (40).

Because no closed-form solution to the corresponding CME exists, the authors use neural networks to learn approximate solutions (40). With approximate solutions to the CME, they infer the biophysical parameters corresponding to burst size b, and relative splicing and degradation rates β/α and γ/α, respectively, using maximum likelihood estimates from scRNA-seq data (40). The authors show that their inference method recovers the same biophysical parameters from a mouse primary motor cortex scRNA-seq data set as accurately as previously developed CME-approximation methods, but orders of magnitude faster. They note that, because their approach can flexibly infer parameters from multivariate systems, it could be adapted to interpret results from ATAC-seq experiments

in which both chromatin accessibility and RNA counts are available (40). These methods, which use CME representations of RNA dynamics, can more faithfully represent the underlying biophysical processes and will likely improve RNA velocity estimates, although the inference strategies are significantly more complex. However, the incorporation of RNA velocity estimates can provide valuable information to infer the direction of edges in cell-state lineage inference (33) and inference of GRNs (37), so their accuracy is a priority.

Cortical condensates

Our final example demonstrates the use of phase portraits calculated from fluorescence microscopy time series data sets. In (13), the authors set out to understand growth dynamics of cortical condensates, transient assemblies of F-actin, N-wasp, and Arp2/3 that form in the actomyosin cortex of *C. elegans* oocytes and embryos during activation of the cortex. F-Actin and N-wasp were fluorescently labeled, which allowed the authors to track the amount of each molecule over time in thousands of individual condensates (Fig. 7 *A*). Using this large ensemble of coupled time series, they constructed a phase portrait by calculating the average time rate of change of F-actin and N-wasp as a function of the amount of F-actin and N-wasp in the condensate (13). In this framework, cortical condensates are represented as a 2D dynamical system whose phase space is defined by the numbers of F-actin and N-wasp molecules in the condensate at a given time. The average time rates of change computed from the time series then constitute vectors associated to each neighborhood in phase space (Fig. 7 *B*). Altogether, the phase portrait indicates that cortical condensate growth dynamics are well described by homoclinic orbits, in which N-wasp and F-actin first grow, N-wasp then falls while F-actin continues to grow, and finally both fall until the condensate disappears (13). Therefore, any system of equations that describes these dynamics must recapitulate such homoclinic orbits.

To find these equations, the authors used the observation from the phase portrait that cortical condensate dynamics appeared to depend on the volume fraction of F-actin and N-wasp within condensates. They plotted the relative growth rates of N-wasp, $\dot{W}/W$, as a function of the F-actin volume fraction, $\phi_A = v_A A/V$, where $\dot{W}$ is the time derivative of N-wasp, v_A is a scalar that relates F-actin fluorescence intensity to molecular volume, and V is the time-dependent condensate volume. A similar comparison was made for relative F-actin growth rates versus N-wasp volume fractions. Plots of the data revealed linear relationships, i.e.,

$$\frac{\dot{W}}{W} = k_r - \frac{k_f}{v_A}\phi_A, \frac{\dot{A}}{A} = \frac{k_l}{v_W}\phi_W - k_d \quad (13)$$

where $\phi_W = v_W W/V$ is the volume fraction of N-wasp and v_W relates N-wasp fluorescence intensity to molecular volume, and the k_i are rates determined by linear fits. Rearranging these equations reveals a nonlinear dynamical system that describes the growth dynamics of cortical condensates and correctly produces the homoclinic orbits described by the inferred phase portrait (Fig. 7 *B*) (13):

$$\dot{W} = k_r W - k_l \frac{AW}{V}, \dot{A} = k_b \frac{AW}{V} - k_d A \quad (14)$$

These equations represent effective descriptions of the interactions between F-actin and N-wasp in cortical condensates, which coarse-grain away other molecules that are present, including Arp2/3, which is nucleated by N-wasp and promotes F-actin branching, and other actin regulators.

With this description, the authors observed oocytes under mild and moderate Arp2/3 knockdown by RNAi and refit the model parameters. They found that the dominant effect was the F-actin depolymerization rate k_d increased as Arp2/3 levels were reduced (13). For larger increases of k_d the system of ODEs exhibits a bifurcation in which the homoclinic orbit is replaced by unbounded growth due to the F-actin nullcline moving above the N-wasp nullcline (Fig. 7, *C* and *D*) (13). To test this prediction, the authors used very strong knockdowns of Arp2/3 and observed that cortical condensates disappeared and were replaced by large stable patches of F-actin and N-wasp, as predicted by the phase portrait after the bifurcation (Fig. 7 *D*). In this study, inference of the interaction dynamics before perturbation enabled a precise understanding of the system once perturbations were performed, which would not have been interpretable otherwise, since the perturbation destroyed the cortical condensate dynamics. Investigation of fluctuations in this system and their effects on phase portrait inference due to multiplicative noise (59,60) are the subjects of ongoing research. Also, we note similarities in the molecular components and the temporal ordering of their recruitment in cortical condensates to those observed in actin patches associated with sites of endocytosis (61) and podosomes (62). It is possible that cortical condensates may share features of broadly conserved mechanisms of branched actin growth and/or are implicated in other cellular processes.

Further discussion of dynamical systems inference algorithms and the connections between dynamical systems approaches and causal inference can be found in supporting material, section 4: dynamical systems.

CONCLUSION AND FUTURE OUTLOOK

All of the previously described techniques have advantages and drawbacks, which we briefly summarize. Causal inference provides more precise information than statistical dependencies and correlations, including directed interactions,

systematic methods for removing spurious links, distinguishing between direct and indirect causes, and accounting for unobserved variables. However, the precise nature of the interactions are not provided by causal inference. On the other hand, dynamical systems techniques provide the most detailed descriptions of interactions as systems of ODEs. They require the most domain knowledge and their results are dependent upon the specific variables chosen to represent phase space. They do not explicitly account for causal interactions and thus interpretation of their results in the causal context is not always clear.

We therefore suggest that a possible fruitful path of research is to combine the benefits of causal inference with dynamical systems approaches, as was demonstrated with CellRank in which RNA velocity estimates, which leverage dynamical systems approaches, were used to infer directionality in cell-state lineages. On the other hand, causal inference could precede more detailed characterization. Once a DAG has been inferred, phase portraits or equation discovery techniques could be used to learn the governing equations of those direct interactions. In these ways, the benefits of both approaches are leveraged. The following study takes the latter approach. In (63), the authors take as input a directed network topology and time series data and use dynamic network theory to learn the equations governing the interaction dynamics and to refine the network topology estimation. Another example establishes a connection between a dynamical systems approach and DAGs to understand cell-state lineage and GRN inference (64). The authors use an abstract representation of cell states that can apply equally well to GRNs and, using geometric descriptions of systems of differential equations, they enumerate all ways in which three-way state transitions can be made with at most two tunable parameters (64). They show a correspondence of these state transitions with DAGs, in which transitions correspond to bifurcations of the dynamical system. Finally, they suggest methods for dimensionality reduction such that their methods can apply to the complex settings in developmental biology, and suggest techniques to fit to time-lapse data. Further studies in these directions are warranted.

Other important advances will include extending these techniques to incorporate spatial information and stochasticity. Recent work on spatial causal inference (65) and PDE learning (66–68) will be critical in the endeavor to incorporate spatial information and arrive at a complete description of cells. The CME approach described in RNA velocity inference (40) and techniques for inferring noise structures from data (69) will be essential in developing full pictures of the stochastic processes that ultimately underlie all of cell biology.

It is the authors' view that inference of interaction networks using the described approaches will be essential to gaining a physical understanding of cellular and developmental biology. Inference from observational data using any or all of the techniques discussed in this review should ideally precede interpretation of perturbation experiments whenever possible. In this manner, researchers can build up models that have as much detail as possible and make precise predictions. Such inferences will minimize the risk of network rewiring entailed by perturbative experiments. Of course, perturbations will be necessary to confirm any inferences but, with a robust set of predictions from observational data, results of perturbation experiments can be framed within their context and interpreted to the greatest extent possible. Also, time will be saved since resulting experiments will address focused, precise questions. As progress in observational and analysis capabilities continues, an unprecedented understanding of developmental and cellular biology is sure to follow.

ACKNOWLEDGMENTS

S.W.G. and I.S. acknowledge support from the Max Planck Society, and I.S. acknowledges support from the Alexander von Humboldt Foundation.

AUTHOR CONTRIBUTIONS

I.S. and S.W.G. wrote the paper.

DECLARATION OF INTERESTS

The authors declare no competing interests.

SUPPORTING MATERIAL

Supporting material can be found online at https://doi.org/10.1016/j.bpj.2024.12.003.

REFERENCES

1. Muller, H. J. 1927. Artificial Transmutation of the Gene. *Science*. 66:84–87. https://doi.org/10.1126/science.66.1699.84.
2. Crow, J. F., and S. Abrahamson. 1997. Seventy Years Ago: Mutation Becomes Experimental. *Genetics*. 147:1491–1496. https://doi.org/10.1093/genetics/147.4.1491.
3. Flavell, R. A., D. L. Sabo, …, C. Weissmann. 1975. Site-directed mutagenesis: effect of an extracistronic mutation on the in vitro propagation of bacteriophage Qbeta RNA. *Proc. Natl. Acad. Sci. USA*. 72:367–371. https://doi.org/10.1073/pnas.72.1.367.
4. Hutchison, C. A., 3rd, S. Phillips, …, M. Smith. 1978. Mutagenesis at a specific position in a DNA sequence. *J. Biol. Chem.* 253:6551–6560. https://www.sciencedirect.com/science/article/pii/S0021925819469676.
5. Adli, M. 2018. The CRISPR tool kit for genome editing and beyond. *Nat. Commun.* 9:1911. https://doi.org/10.1038/s41467-018-04252-2.
6. Jinek, M., K. Chylinski, …, E. Charpentier. 2012. A Programmable Dual-RNA–Guided DNA Endonuclease in Adaptive Bacterial Immunity. *Science*. 337:816–821. https://doi.org/10.1126/science.1225829.
7. Jinek, M., A. East, …, J. Doudna. 2013. RNA-programmed genome editing in human cells. *Elife*. 2:e00471. https://doi.org/10.7554/eLife.00471.
8. Agrawal, N., P. V. N. Dasaradhi, …, S. K. Mukherjee. 2003. RNA Interference: Biology, Mechanism, and Applications. *Microbiol. Mol. Biol. Rev.* 67:657–685. https://doi.org/10.1128/mmbr.67.4.657-685.2003.

9. Sen, G. L., and H. M. Blau. 2006. A brief history of RNAi: the silence of the genes. *Faseb. J.* 20:1293–1299. https://doi.org/10.1096/fj.06-6014rev.

10. Fire, A., S. Xu, …, C. C. Mello. 1998. Potent and specific genetic interference by double-stranded RNA in Caenorhabditis elegans. *Nature.* 391:806–811. https://doi.org/10.1038/35888.

11. Kirkham, M., T. Müller-Reichert, …, A. A. Hyman. 2003. SAS-4 Is a C. elegans Centriolar Protein that Controls Centrosome Size. *Cell.* 112:575–587. https://www.sciencedirect.com/science/article/pii/S009286740300117X.

12. Naganathan, S. R., S. Fürthauer, …, S. W. Grill. 2018. Morphogenetic degeneracies in the actomyosin cortex. *Elife.* 7:e37677. https://doi.org/10.7554/eLife.37677.

13. Yan, V. T., A. Narayanan, …, S. W. Grill. 2022. A condensate dynamic instability orchestrates actomyosin cortex activation. *Nature.* 609:597–604. https://doi.org/10.1038/s41586-022-05084-3.

14. Brauns, F., L. Iñigo de la Cruz, …, E. Frey. 2023. Redundancy and the role of protein copy numbers in the cell polarization machinery of budding yeast. *Nat. Commun.* 14:6504. https://doi.org/10.1038/s41467-023-42100-0.

15. Liu, Z., D. Miller, …, S. F. Levy. 2020. A large accessory protein interactome is rewired across environments. *Elife.* 9:e62365. https://doi.org/10.7554/eLife.62365.

16. Davis, M. J., C. J. Shin, …, M. A. Ragan. 2012. Rewiring the dynamic interactome. *Mol. Biosyst.* 8:2054–2066. https://doi.org/10.1039/C2MB25050K.

17. The Astronomers Tycho Brahe and Johannes Kepler. https://chandra.harvard.edu/edu/formal/icecore/The_Astronomers_Tycho_Brahe_and_Johannes_Kepler.pdf.

18. Blair, A. 1990. Tycho Brahe's Critique of Copernicus and the Copernican System. *J. Hist. Ideas.* 51:355–377. http://www.jstor.org/stable/2709620.

19. Kuhn, T. S. 1957. The Copernican Revolution. Harvard University Press.

20. Wilson, C. 1968. Kepler's Derivation of the Elliptical Path. *Isis.* 59:4–25. http://www.jstor.org/stable/227848.

21. Wilson, C. 1974. Newton and Some Philosophers on Kepler's "Laws". *J. Hist. Ideas.* 35:231–258. http://www.jstor.org/stable/2708760.

22. Caspar, M. 1994. Kepler. Dover Publications Inc.

23. Einstein, A. 1914. Covariance Properties of the Field Equations of the Theory of Gravitation Based on the Generalized Theory of Relativity. *Z. Math. Phys.* 63:215–225.

24. Earman, J., and C. Glymour. 1980. Relativity and Eclipses: The British Eclipse Expeditions of 1919 and Their Predecessors. *Hist. Stud. Phys. Sci.* 11:49–85. https://doi.org/10.2307/27757471.

25. Abbott, B. P., R. Abbott; …, LIGO Scientific Collaboration and Virgo Collaboration. 2016. Observation of Gravitational Waves from a Binary Black Hole Merger. *Phys. Rev. Lett.* 116:061102. https://doi.org/10.1103/PhysRevLett.116.061102.

26. Stumpf, M. P. H., T. Thorne, …, C. Wiuf. 2008. Estimating the size of the human interactome. *Proc. Natl. Acad. Sci. USA.* 105:6959–6964. https://doi.org/10.1073/pnas.0708078105.

27. Levchenko, A., and I. Nemenman. 2014. Cellular noise and information transmission. *Curr. Opin. Biotechnol.* 28:156–164. https://www.sciencedirect.com/science/article/pii/S0958166914000925.

28. Stumpf, P. S., R. C. G. Smith, …, B. D. MacArthur. 2017. Stem Cell Differentiation as a Non-Markov Stochastic Process. *Cell Syst.* 5:268–282.e7. https://doi.org/10.1016/j.cels.2017.08.009.

29. Mangalam, M., and D. G. Kelty-Stephen. 2022. Ergodic descriptors of non-ergodic stochastic processes. *J. R. Soc. Interface.* 19:20220095. https://doi.org/10.1098/rsif.2022.0095.

30. Weigel, A. V., B. Simon, …, D. Krapf. 2011. Ergodic and nonergodic processes coexist in the plasma membrane as observed by single-molecule tracking. *Proc. Natl. Acad. Sci. USA.* 108:6438–6443. https://doi.org/10.1073/pnas.1016325108.

31. La Manno, G., R. Soldatov, …, P. V. Kharchenko. 2018. RNA velocity of single cells. *Nature.* 560:494–498. https://doi.org/10.1038/s41586-018-0414-6.

32. Briggs, J. A., C. Weinreb, …, A. M. Klein. 2018. The dynamics of gene expression in vertebrate embryogenesis at single-cell resolution. *Science.* 360:eaar5780. https://doi.org/10.1126/science.aar5780.

33. Lange, M., V. Bergen, …, F. J. Theis. 2022. CellRank for directed single-cell fate mapping. *Nat. Methods.* 19:159–170. https://doi.org/10.1038/s41592-021-01346-6.

34. Weinreb, C., S. Wolock, …, A. M. Klein. 2018. Fundamental limits on dynamic inference from single-cell snapshots. *Proc. Natl. Acad. Sci. USA.* 115:E2467–E2476. https://doi.org/10.1073/pnas.1714723115.

35. Xu, L., T. Cong, …, X. Lan. 2023. Dissecting cell state transitions by causal inference. Preprint at bioRxiv. https://www.biorxiv.org/content/early/2023/03/25/2023.02.08.527606.

36. Haghverdi, L., M. Büttner, …, F. J. Theis. 2016. Diffusion pseudotime robustly reconstructs lineage branching. *Nat. Methods.* 13:845–848. https://doi.org/10.1038/nmeth.3971.

37. Singh, R., A. P. Wu, …, B. Berger. 2024. Causal gene regulatory analysis with RNA velocity reveals an interplay between slow and fast transcription factors. *Cell Syst.* 15:462–474.e5. https://doi.org/10.1016/j.cels.2024.04.005.

38. Kar, P., S. Tiruvadi-Krishnan, …, A. Amir. 2023. Using conditional independence tests to elucidate causal links in cell cycle regulation in *Escherichia coli*. *Proc. Natl. Acad. Sci. USA.* 120:e2214796120. https://doi.org/10.1073/pnas.2214796120.

39. Gorin, G., M. Fang, …, L. Pachter. 2022. RNA velocity unraveled. *PLoS Comput. Biol.* 18:e1010492. https://doi.org/10.1371/journal.pcbi.1010492.

40. Gorin, G., M. Carilli, …, L. Pachter. 2024. Spectral neural approximations for models of transcriptional dynamics. *Biophys. J.* 123:2892–2901. https://doi.org/10.1016/j.bpj.2024.04.034.

41. Zheng, S. C., G. Stein-O'Brien, …, K. D. Hansen. 2023. Pumping the brakes on RNA velocity by understanding and interpreting RNA velocity estimates. *Genome Biol.* 24:246. https://doi.org/10.1186/s13059-023-03065-x.

42. Spearman, C. 1904. The Proof and Measurement of Association between Two Things. *Am. J. Psychol.* 15:72–101. http://www.jstor.org/stable/1412159.

43. Shannon, C. E. 1948. A Mathematical Theory of Communication. *Bell Syst. Tech. J.* 27:379–423. https://doi.org/10.1002/j.1538-7305.1948.tb01338.x.

44. Jonas Peters, D. J., and B. Schölkopf. 2017. Elements of Causal Inference. MIT Press.

45. Spirtes, P., C. Glymour, and R. Scheines. 2001. Causation, Prediction, and Search. The MIT Press. https://doi.org/10.7551/mitpress/1754.001.0001.

46. Rohrer, J. M. 2018. Thinking Clearly About Correlations and Causation: Graphical Causal Models for Observational Data. *Adv. Methods Practices Psychol. Sci.* 1:27–42. https://doi.org/10.1177/2515245917745629.

47. Ryan, O., L. F. Bringmann, and N. K. Schuurman. 2022. The Challenge of Generating Causal Hypotheses Using Network Models. *Struct. Equ. Model.: A Multidiscip. J.* 29:953–970. https://doi.org/10.1080/10705511.2022.2056039.

48. 2012. A Brief History of Causality. Cambridge University Press, Cambridge, pp. 11–42, Causality, Probability, and Time. https://www.cambridge.org/core/product/C87F30B5A6F4F63F0C28C3156B809B9E.

49. Yuan, A. E., and W. Shou. 2022. Data-driven causal analysis of observational biological time series. *Elife.* 11:e72518. https://doi.org/10.7554/eLife.72518.

50. Rothman, K. J., and S. Greenland. 2005. Causation and Causal Inference in Epidemiology. *Am. J. Publ. Health.* 95:S144–S150. https://doi.org/10.2105/AJPH.2004.059204.

51. Wagner, D. E., C. Weinreb, …, A. M. Klein. 2018. Single-cell mapping of gene expression landscapes and lineage in the zebrafish embryo. *Science.* 360:981–987. https://doi.org/10.1126/science.aar4362.

52. Pearl, J. 2009. Causality, 2 edition. Cambridge University Press.

53. Janzing, D., D. Balduzzi, …, B. Schölkopf. 2013. Quantifying causal influences. *Ann. Stat.* 41:2324–2358. https://doi.org/10.1214/13-AOS1145.

54. Janson, N. B. 2012. Non-linear dynamics of biological systems. *Contemp. Phys.* 53:137–168. https://doi.org/10.1080/00107514.2011.644441.

55. Tyson, J. J., and B. Novak. 2020. A Dynamical Paradigm for Molecular Cell Biology. *Trends Cell Biol.* 30:504–515. https://www.sciencedirect.com/science/article/pii/S0962892420300738.

56. Tyson, J. J., K. C. Chen, and B. Novak. 2003. Sniffers, buzzers, toggles and blinkers: dynamics of regulatory and signaling pathways in the cell. *Curr. Opin. Cell Biol.* 15:221–231. https://www.sciencedirect.com/science/article/pii/S0955067403000176.

57. Izhikevich, E. M. 2006. Dynamical Systems in Neuroscience: The Geometry of Excitability and Bursting. The MIT Press. https://doi.org/10.7551/mitpress/2526.001.0001.

58. Bergen, V., M. Lange, …, F. J. Theis. 2020. Generalizing RNA velocity to transient cell states through dynamical modeling. *Nat. Biotechnol.* 38:1408–1414. https://doi.org/10.1038/s41587-020-0591-3.

59. Volpe, G., and J. Wehr. 2016. Effective drifts in dynamical systems with multiplicative noise: a review of recent progress. *Rep. Prog. Phys.* 79:053901. https://doi.org/10.1088/0034-4885/79/5/053901.

60. Volpe, G., L. Helden, …, C. Bechinger. 2010. Influence of Noise on Force Measurements. *Phys. Rev. Lett.* 104:170602. https://doi.org/10.1103/PhysRevLett.104.170602.

61. Kaksonen, M., Y. Sun, and D. G. Drubin. 2003. A Pathway for Association of Receptors, Adaptors, and Actin during Endocytic Internalization. *Cell.* 115:475–487. https://doi.org/10.1016/S0092-8674(03)00883-3.

62. Linder, S., P. Cervero, …, J. Condeelis. 2023. Mechanisms and roles of podosomes and invadopodia. *Nat. Rev. Mol. Cell Biol.* 24:86–106. https://doi.org/10.1038/s41580-022-00530-6.

63. Gao, T.-T., and G. Yan. 2022. Autonomous inference of complex network dynamics from incomplete and noisy data. *Nat. Comput. Sci.* 2:160–168. https://doi.org/10.1038/s43588-022-00217-0.

64. Rand, D. A., A. Raju, …, E. D. Siggia. 2021. Geometry of gene regulatory dynamics. *Proc. Natl. Acad. Sci. USA.* 118:e2109729118. https://doi.org/10.1073/pnas.2109729118.

65. Reich, B. J., S. Yang, …, A. Rappold. 2021. A Review of Spatial Causal Inference Methods for Environmental and Epidemiological Applications. *Int. Stat. Rev.* 89:605–634. https://doi.org/10.1111/insr.12452.

66. Rudy, S. H., S. L. Brunton, …, J. N. Kutz. 2017. Data-driven discovery of partial differential equations. *Sci. Adv.* 3:e1602614. https://doi.org/10.1126/sciadv.1602614.

67. Raissi, M., P. Perdikaris, and G. Karniadakis. 2019. Physics-informed neural networks: A deep learning framework for solving forward and inverse problems involving nonlinear partial differential equations. *J. Comput. Phys.* 378:686–707. https://www.sciencedirect.com/science/article/pii/S0021999118307125.

68. Chen, Z., Y. Liu, and H. Sun. 2021. Physics-informed learning of governing equations from scarce data. *Nat. Commun.* 12:6136. https://doi.org/10.1038/s41467-021-26434-1.

69. Craigmile, P., R. Herbei, …, G. Schneider. 2023. Statistical inference for stochastic differential equations. *WIREs Comput. Stats.* 15:e1585. https://doi.org/10.1002/wics.1585.

Biophysical Journal, Volume *124*

Supplemental information

Empirical methods that provide physical descriptions of dynamic cellular processes

Ian Seim and Stephan W. Grill

Supplemental Information for: Empirical methods that provide physical descriptions of dynamic cellular processes

Ian Seim[1,*] and Stephan Grill[1,2,3,*]

[1]Max Planck Institute of Molecular Cell Biology and Genetics, Dresden, Germany
[2]Center for Sytems Biology Dresden (CSBD), Dresden, Germany
[3]Cluster of Excellence Physics of Life, TU Dresden, Dresden, Germany
[*]Correspondence: seim@mpi-cbg.de, grill@mpi-cbg.de

Contents

1 Causal inference from observations **2**
1.1 Causal inference in the presence of unobserved variables 2
1.2 non-linear SCMs with non-Gaussian noise and do-Calculus 3
1.3 Causal inference algorithms for time series data 4

2 Correlations and Dependence among and within Time Series **5**

3 Undirected network inference **6**
3.1 Network inference for data collected at a single time point 6
3.2 Inference for undirected time-varying networks using time series data 7

4 Dynamical Systems **9**
4.1 Relationships between dynamical systems approaches and causal inference 9
4.2 Leveraging dynamical systems theory to learn DAGs 9
4.3 Automated discovery of differential equations 10

1 Causal inference from observations

A central goal of causal inference is to establish the smallest set of possible DAGs that are consistent with the conditional independence patterns in a dataset, called a *Markov equivalence class* [1]. As we have seen, major issues for determining causal relations are determining the direction of interactions and dealing with spurious links arising from common causes (**Fig 2**) and common effects (**Fig 3**). The fundamental advance of causal inference is to consider conditional dependence between pairs of variables given appropriate *subsets* of other variables in the network. We briefly discuss some strategies that use such statistical tests to address the challenges of inferring directional interactions and removing spurious links due to common effects and unobserved common causes.

The first approach, called the PC algorithm [1], addresses common effects and assumes *causal sufficiency*, which requires that all common causes are observed. It fundamentally relies on the concept of *d-separation* which defines conditional independence between variables dependent on subsets of other variables in the network. Specifically, X and Y are d-separated given the set of variables $\boldsymbol{W}$ which does not contain X or Y iff X and Y are conditionally independent given $\boldsymbol{W}$ [1]. The PC algorithm works by starting with an undirected graph in which all nodes are connected (**Fig S1A**). Each pair of nodes X and Y is evaluated with respect to sets of other nodes, $\boldsymbol{W}$, to find if they can be d-separated conditional on $\boldsymbol{W}$, where $\boldsymbol{W}$ iteratively contains $0, 1, 2, \ldots$ nodes. If there is any $\boldsymbol{W}$ including the empty set for which X and Y are d-separated, the edge between them is removed. This step removes links in which nodes are independent, when they are dependent due to common and indirect causes, is not subject to spurious links due to common effects, and leaves an undirected graph in which all edges correspond to direct causal interactions (**Fig S1B**). Next, as many edges as possible are oriented. For all triplets X, Y, and Z in which X, Z and Y, Z are adjacent, but X, Y are not, then $X \rightarrow Z \leftarrow Y$ if X and Y are not d-separated for every subset of nodes $\boldsymbol{V}$ which contains Z. This step correctly identifies common effect topologies (**Fig S1C**). The final step relies upon the remaining possible topologies given proper identification of all common effects in the previous step. Specifically, if $X \rightarrow Z$, Z is connected to U, and X is not connected to U, then $Z \rightarrow U$ (**Fig S1D**). This inference is possible because the conditional independence tests used in the previous step to identify $X \rightarrow Z \leftarrow Y$ would not have yielded such a topology if $U \rightarrow Z$ [1]. Common effects are therefore the basis upon which the PC algorithm assigns directionality to interactions. This procedure results in a graph with a combination of directed and undirected edges that represents a family of graphs which are all compatible with the data, since undirected edges can be oriented either way and still explain the data. Such a graph is called a Markov equivalence class. No links are spurious and each explains a direct causal interaction, so the PC algorithm is a major advance over the previous approaches discussed in this review.

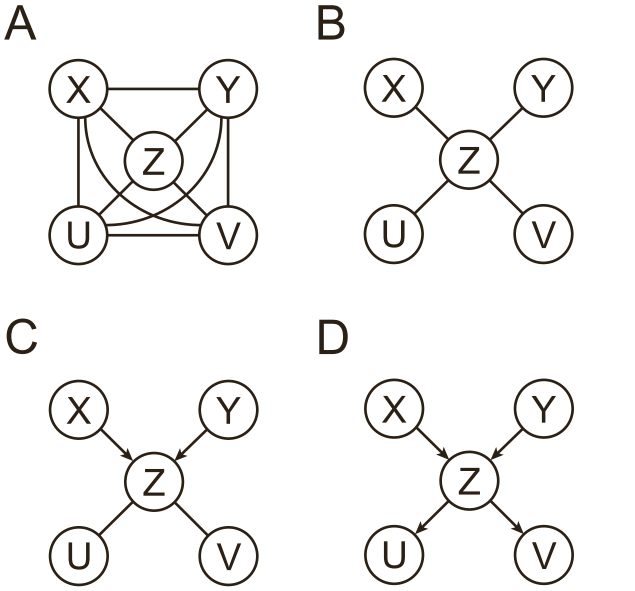

Figure S1: **The PC algorithm.** The DAG to be inferred is shown in panel D. A) The algorithm begins with all nodes connected by undirected edges. B) Conditional independence tests leave the causal skeleton. C) All colliders are oriented. D) Edges connecting colliders to their descendants can be oriented.

1.1 Causal inference in the presence of unobserved variables

The PC algorithm assumes causal sufficiency in which all common causes are observed. However, this condition is often not met in practice. How can we cope with unobserved common causes? In particular, if we detect that X and Y are dependent, but have not measured some common cause Z, how do we determine whether or not Z exists or if there is a causal relation among X and Y using only observations of X and Y? A possible solution is the fast causal inference (FCI) algorithm, introduced in [1]. The underlying concepts and algorithm implementation are too complicated to discuss in detail here and can be found in Chapter 6 of [1]. The output of the FCI algorithm is a graph with 4 edge types that denote the following: causal (direct) interactions, undirected interactions, an edge which indicates either a direct interaction or a common cause, or an edge which indicates the presence of an unobserved common cause for pairs of variables between which there is no direct interaction (**Fig S2**). Although unobserved variables reduce the certainty in the inferences, it is remarkable that such details about interactions can still be inferred, and that unobserved common causes can be definitively detected (and their measurement possibly prioritized for future experiments).

Further assumptions about the functional forms of the interactions among nodes can go further than con-

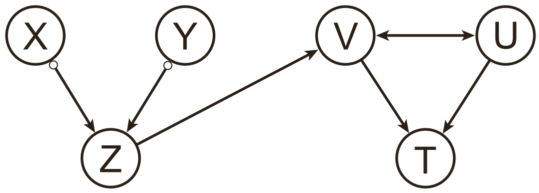

Figure S2: **Example output of the FCI algorithm.** The edge from X to Z indicates either a direct interaction or a common cause, the edge from Z to V indicates a direct interaction, and the edge between V and U indicates the presence of an unobserved common cause and *no* direct interaction between V and U.

ditional independence tests in determining network structures in the presence of unobserved variables. In Chapter 6 [1], the authors discuss some situations assuming linear relationships in which analysis of specific combinations of correlations among variables, called tetrad differences, allows for the inference of the presence and structure of unobserved variables. Finally, the concept of *instrumental variables* can provide avenues for making causal inferences in the presence of unobserved variables. If we wish to identify whether or not X causes Y in the presence of unobserved variables $\boldsymbol{U}$ which may cause both X and Y, the variable Z is an instrumental variable if it causes X but not Y and shares no common causes with Y (**Fig S3**). Then the effect of X on Y can be estimated by the ratio of the effect of Z on Y to the effect of Z on X, even in the presence of the unobserved variables $\boldsymbol{U}$ [2]. The use of instrumental variables is subject to certain assumptions and their identification is non-trivial [2].

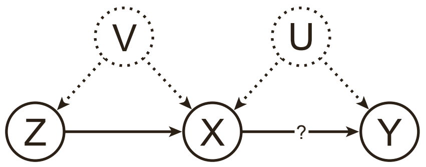

Figure S3: **Instrumental variables.** Z is an instrumental variable since it directly causes X and has no common causes with Y, and can help to answer whether X causes Y. V and U indicate unobserved common causes. Note that Z can share common causes with X.

All of the methods discussed above rely on conditional independence tests, which are subject to error with small amounts of data and noise. In practice, testing for conditional independence can be challenging, but partial correlation coefficients, conditional mutual information, or other techniques that best capture the dependence structures in the data are available [3, 4]. However, inference can be bolstered with previous knowledge about the presence/absence and direction of edges among nodes. Any information about time ordering of variables can constrain conditional independence tests (if Y occurs after X, then X should not be conditioned on Y) and help to determine directionality of edges (if X and Y are connected and Y occurs after X, then $X \to Y$) [1]. The PC and FCI algorithms are also feasible for large numbers of variables if the underlying graphs are sparse, which we have already established is the norm for biological networks [5, 6, 7].

1.2 non-linear SCMs with non-Gaussian noise and do-Calculus

Kar *et al* assumed linear relationships and additive Gaussian noise in building SCMs, but many processes are not well-described with linear functions or Gaussian noise. Therefore, there have been several efforts to extend techniques to non-linear [8] and non-Gaussian [9] SCMs. The authors of both of these studies find that non-linearity and non-Gaussianity can actually constrain the plausible set of causal models which fit the data and even lead to identification of unique models, as opposed to the Markov equivalence classes that are generally the output of the PC and FCI algorithms. The central idea is that both non-Gaussian noise and non-linearity can help to determine the direction of interactions, whereas the PC algorithm can only assign directions using common effects and their descendants [8, 9, 1].

Structural causal models are central to causal inference because they allow for prediction of the effects of perturbations and for reasoning about hypothetical situations. A primary set of relevant analysis tools is *do-Calculus*, invented by Judea Pearl [10]. do-Calculus allows one to compute distributions of Y given that X is set to a value x in a structural causal model, i.e. $P(Y|\mathrm{do}\, X = x)$, which is in general not the same as $P(Y|x)$ [11]. However, according to Pearl's causal hierarchy [10], there is a level of causal reasoning that subsumes even prediction of the effects of perturbations, called *counterfactual* reasoning. Counterfactuals are statements like "If x had happened, then y would have been different." They are retrospective hypotheticals which use present observations to reason about alternative scenarios. Therefore, they can leverage more information since they include information given observations. One can create counterfactual SCMs by conditioning the noise terms on the given observations x and then performing the do-Calculus operation one is interested in [12, 11]. Since the conditioning only affects the noise variables, the underlying DAG is unaltered, but the effects of perturbations given our current knowledge from observations is in general different than when using only prior distributions for the noise [12]. A recent study used counterfactuals and do-Calculus to study Zebrafish development [13]. The authors identified molecular mechanisms for activation of two Zebrafish *nodal* genes that control mesoderm and endoderm induction using do-Calculus to analyze fluorescence in situ hybridization (FISH) images of the genes of interest [13]. SCMs and counterfactual SCMs are extremely important tools in causal inference, and recent efforts to incorporate them into machine learning algorithms are the foundation of a new field called

causal machine learning [11, 14]. The hope in causal machine learning is that incorporation of causal inference can allow for more precise interpretations of and predictions from data than the standard statistical associations produced by most machine learning models.

1.3 Causal inference algorithms for time series data

Causal inference using time series data becomes both easier and more difficult due to time ordering of variables. Since causality can only flow forward in time, the space of possible directional interactions is smaller in one sense. However, since variables can be represented as a series of nodes at each time point (**Fig 4**), inference of causal links between variables at specific time lags is desired which expands the space of possible connections. Additionally, many inference techniques rely on the assumption of independent noise terms, but many real-world time series are auto-correlated which violates that assumption. Stationarity is also a common assumption of causal inference techniques for time series that is often violated by real time series. Therefore, there are extra precautions that must be taken when working with time series.

There are many approaches to causal inference for time series including generalization of the DAGs and SCMs discussed in the previous section and generalizations of information theoretical concepts, among others. We will briefly discuss some of these approaches, beginning with DAGs and SCMs. More extensive reviews of causal inference for time series can be found in [15, 16].

A recent approach from Runge *et al* relies on SCMs generalized to stochastic processes and uses variants of the PC algorithm and conditional independence tests to infer causal links in auto-correlated, non-linear time series [17]. Specifically, the first step of their algorithm PCMCI uses a variant of the PC algorithm to create a subset of possible direct causes for a given node X_t in a way analogous to the procedure described above. The second step uses momentary conditional independence (MCI) tests to further trim the set of possible direct causes and accounts for auto-correlations [17]. The specific conditional independence test can be chosen by the user, and the authors reviewed the performance of partial correlations, Gaussian process regression, and conditional mutual information. The latter two can detect non-linear dependencies but have lower power to detect linear relationships for small sample sizes [17]. The authors show that PCMCI can correctly reconstruct the Walker circulation, a well-understood model of air flow in the tropics, from observational climate data. They also demonstrate the algorithm's efficacy on data of heart rate and blood pressure and on large synthetic datasets. The PCMCI algorithm assumes causal sufficiency and stationarity, although it was able to correctly infer causal links even in the presence of known non-stationarity. To relax the assumption of causal sufficiency, the LPCMCI was developed [18]. It is based on variants of the FCI algorithm introduced previously and can thus account for unobserved variables. However, it relies heavily on stationarity. Several alternative techniques have been proposed to account for and even exploit non-stationarity for inference [19, 20, 21].

An alternative approach to causal inference for time series generalizes the concept of conditional mutual information to variables separated in time, and is called the *transfer entropy* [22]. The transfer entropy measures the amount of information transfer from X_t to Y_t and can be defined as a conditional mutual information:

$$T_{X\to Y} = I(Y_t; X_{t-1:t-L}|Y_{t-1:t-L}) \qquad (1)$$

where $X_{t-1:t-L}$ are the values of X_t at times $t-L, t-L+1, \ldots, t-1$ and similarly for Y_t. Therefore, the transfer entropy is the mutual information between Y_t and past values of X_t conditioned on past values of Y_t. It is an inherently asymmetric measure of information flow due to the inclusion of time lags, but as a measure of information flow includes information from not only direct causes but also indirect causes and common causes. Therefore, for accurate causal inference, additional steps must be taken in addition to computing the transfer entropy among pairs of variables. However, it provides a quantification of information transfer between variables which is an advantage over previous methods. The authors in [23] estimate transfer entropy to measure information flow between the cell signaling proteins SOS and RAF. They recorded time series of the intensities of fluorescently labeled SOS and RAF in HeLa cells upon activation of the upstream cell surface receptor protein ERBB with epidermal growth factor (EGF). They studied the wild-type system and a mutant system in which SOS has a point mutation associated with Noonan syndrome with the goal of identifying a malfunctioning molecular mechanism underlying the disease. The authors assume that pairs of time series can be regarded as multivariate Gaussian distributions at each time, which is a valid assumption in the case of Gaussian noise [23]. This assumption allows them to compute transfer entropy using covariance matrices and allows for non-stationarity if the noise is Gaussian. Using this approach in wild-type cells, they detect a flow of information first from SOS to RAF, and later from RAF to SOS, consistent with the known presence of a negative feedback mechanism. In the mutant cells, however, they detected information flow only from SOS to RAF, suggesting that the mutant SOS abrogates the negative feedback mechanism [23]. Although their method does not account for the possibility of unobserved common causes, it does generate predictions that can be directly tested. Other approaches have also successfully measured transfer entropy in non-stationary time series using ensembles of measurements [24, 25].

2 Correlations and Dependence among and within Time Series

In practice, we often have the advantage (complication) of working with datasets which comprise time series of multiple variables of interest. As an example, we can imagine that we measure the total cellular fluorescence intensities of two tagged proteins, x and y, at a series of times, $t_0, t_1, t_2, \ldots, t_n$. For cell i, we now have two time series corresponding to observations of the two proteins, $x_i^t = \{x_i(t = 0), x_i(t = 1), x_i(t = 2), \ldots, x_i(t = n)\}$ and y_i^t defined analogously. If we record time series for N cells and manage to temporally align them among cells (a non-trivial task), we now have an ensemble of time series $\{x_i^t\}$ and $\{y_i^t\}$. These ensembles can be regarded as realizations of the stochastic processes, $\{X_t\}$ and $\{Y_t\}$. These objects are collections of the random variables X_t and Y_t, which are generalizations of our previous variables X and Y to the times $t_0, t_1, t_2, \ldots, t_n$. X_{t_i} can have a different probability distribution than X_{t_j}. In our example of protein expression, time t_i could be during the G_1 cell cycle phase, and the time t_j could be during S phase. It is likely that large-scale rearrangements of gene regulatory networks have occurred in this time interval leading to a different expression pattern for our protein, and thus distinct probability distributions for its expression at times t_i and t_j. It is not hard to imagine that such a feature complicates analysis of time series and compromises resulting predictions. Therefore, a common requirement for time series analysis is the assumption that the underlying stochastic processes are *stationary*, i.e. for the stochastic process $\{X_t\}$, all of the random variables X_t have the same probability distribution. In practice, it is often sufficient for a process to be *weakly stationary*, in which the means and variances of the X_t are constant, and the covariances among X_{t_i} and X_{t_j} only depend on the time shift, $s = t_i - t_j$, and not on t_i. However, real-world phenomena often comprise both deterministic and stochastic components. A deterministic component implies a mean value that changes with time which violates (weak) stationarity. Such processes can be (weakly) *trend-stationary*, though, if the deterministic process (trend) can be modeled and fit, leaving residuals which are a (weakly) stationary process. A process $\{Z_t\}$ is trend-stationary if $Z_t = f(t) + X_t$ for any function f and a stationary stochastic process $\{X_t\}$ [26]. De-trending procedures can yield time series that are more amenable to statistical analyses that interpret fluctuations [27, 28]. Experimentally, trend-stationarity in cells corresponds to collecting observations during a sufficiently short time period such that the interaction networks relevant to the dynamics of proteins x and y have not changed.

An obvious advantage of time series data comes from the notion that causality can only propagate forward in time since events in the future cannot affect events in the past. Time series data therefore constrain the possible causal relationships such that, for a given time t we need only consider the effect(s) of $\{X_t\}$ on $\{Y_s\}$ for $s > t$ and vice versa. However, detection of dependence among stochastic processes becomes more complex relative to the case of random variables due to the possibilities of non-stationarity, discussed above, and auto-correlations. To understand these complications, we first generalize the definitions of independence and Pearson's correlation coefficient to the case of stochastic processes. Two stochastic processes $\{X_t\}$ and $\{Y_t\}$ are independent if

$$P(X_{t_i}, \ldots, X_{t_j}, Y_{t_i}, \ldots, Y_{t_j}) = P(X_{t_i}, \ldots, X_{t_j}) P(Y_{t_i}, \ldots, Y_{t_j}) \quad (2)$$

for all times $t_i, \ldots, t_j$. This condition is evidently a generalization of the previous definition of independence to all subsets of the two stochastic processes in time. Similarly, if this condition is violated, the stochastic processes are dependent. We also need to generalize the concept of correlation to apply to observations through time. We can calculate a time-dependent Pearson's correlation coefficient by computing the previously defined correlation coefficient as a function of time, i.e.

$$\rho_{\{X_t\},\{Y_t\}}(t_i, t_j) = \frac{\mathrm{Cov}(X_{t_i}, Y_{t_j})}{\sigma_{X_{t_i}} \sigma_{Y_{t_j}}} \quad (3)$$

This value is called the *cross-correlation* and quantifies correlations among the processes $\{X_t\}$ and $\{Y_t\}$ for all times t_i and t_j. For our protein expression example, the cross-correlation quantifies correlations among the sets of observations $\{x_i^{t_i}\}$ and $\{y_i^{t_j}\}$ for all cells $i = 1, \ldots, N$ and times t_i, t_j. If the two processes are not *jointly stationary*, then the cross-correlation depends on all times t_i and t_j, and there is not much predictive power gained from its calculation. However, if the processes are *jointly weakly stationary*, then the cross-correlation no longer depends on the time t_i but only on the time shift $s = t_i - t_j$. Its calculation reveals predictable correlations, or the lack of them, between two processes. We can also consider the correlations of a stochastic process with itself at different times, called the *auto-correlation.* The auto-correlation is defined as the cross-correlation with $\{Y_t\}$ replaced by $\{X_t\}$ and was used previously in the definition of weak stationarity. In many contexts, a time series will have non-zero auto-correlation due to deterministic components, for example. From our previous understanding of correlation and dependence, we know that if a stochastic process $\{X_t\}$ has non-zero auto-correlation at times t_i and t_j, then its constituent random variables X_{t_i} and X_{t_j} are dependent implying that the X_t are not IID. This can complicate quantification of dependence *among* two auto-correlated time series for the following reasons. If the two auto-correlations are either both positive or both negative over the same time range, there is a danger of detecting spurious cross-correlations when in fact there are none. The intuition for this phenomenon is that positive auto-correlation implies a persistence of values over a period of time. This persistence increases the likelihood that two independent but both positively auto-correlated processes

will co-vary during some range of time $t_i, \ldots, t_j$ by chance, and thus appear to be cross-correlated [29]. Surprisingly, a similar phenomenon occurs for two negatively auto-correlated time series [29]. A detailed examination of this phenomenon and modified significance limits on the sample cross-correlation for auto-correlated time series can be found in [29]. There are several techniques that have been developed to correct for the influence of auto-correlation and/or non-stationarity in the calculation of cross-correlation [15, 29] and generalized cross-correlations like power-law cross-correlation [30, 31].

Evidently, determining dependence among processes using time series data can be complex and subject to uncertainty. However, with proper caution, application of the above techniques may answer whether or not two processes belong to a common interaction network. Next, we discuss techniques which aspire to make deeper statements about the nature of interactions such as their strengths, directions, and quantitative descriptions and which require more or less strict assumptions about stationarity and auto-correlation.

3 Undirected network inference

A natural framework for understanding interaction networks comes from the field of network, or graph theory. In this paradigm, variables are represented as nodes and interactions among variables are encoded as edges which connect nodes. Commonly, for a graph with n nodes, the connectivity is represented by the $n \times n$ adjacency matrix A_{ij}, whose entries encode the edges. Edges can either be directed, which implies a causal relationship, or undirected which implies one of several possible causal relationships. This section is concerned with inference of undirected edges (**Fig 1SB**) which are represented by a symmetric A_{ij}. Although undirected networks make less precise statements about interactions, they are typically easier to compute and rely on fewer assumptions than causal inference methods.

3.1 Network inference for data collected at a single time point

We begin by considering inference of networks from data collected at single points in time. Example observations include RNA-seq and proteomics data, and the task is to reconstruct the underlying connections among genes, or gene regulatory networks (GRNs), which explain the observed levels of RNA or protein. The simplest approach is to calculate correlation coefficients among all pairs of variables. This technique is computationally inexpensive and may provide some rough outline of interactors, but it is subject to all of the drawbacks of correlation coefficients discussed in the previous section. Specifically, one can only infer *effective* interactions among variables that share linear dependence, which can lead to many false positives with respect to *direct* interactions in the case of common causes, and false negatives in the case of non-linear dependence. We will discuss examples of solutions to both of these issues.

To detect non-linear dependence, one can instead calculate the mutual information [32] among all pairs. Pairwise calculation of the mutual information among all variables is not subject to false negatives since it accounts for all statistical dependencies, but it still only gives effective interactions which need to be "trimmed" to reveal the true interaction network.

The ARACNE algorithm, designed to infer gene regulatory networks from microarray expression profiles, introduces a possible solution for isolating direct interactions [33]. First, the mutual information between all pairs is calculated, resulting in an over-connected network. Next, a significance threshold on the information I_0 and its corresponding p-value are calculated by randomly permuting the genes and re-calculating mutual information for pairs which should now be overwhelmingly statistically independent. Connections in the original network with a mutual information below I_0 are removed since they are likely statistically indistinguishable from 0, implying independence of the variables. However, the issue of common causes still persists. To address this, the authors use the data processing inequality (DPI) as applied to the possibility of effective interactions. If we assume that nodes X and Y are both directly connected to node Z but not to each other, than the DPI states [33]:

$$I(X,Y) \leq \min(I(X,Z), I(Y,Z)) \qquad (4)$$

In words, the smallest mutual information will correspond to the spurious interaction between X and Y. ARACNE thus examines all connected gene triplets and removes the edge with the smallest value [33]. The danger of false positives is greatly reduced, and the authors showed that ARACNE can exactly reconstruct networks comprised of only pairwise interactions and without 3-node loops (although higher-order loops are possible) if mutual information can be estimated exactly [33]. The authors used their algorithm on a B lymphocyte expression profile dataset, successfully separating many direct and indirect interactions previously identified biochemically [33].

The PIDC algorithm [34] offers an alternative way to eliminate effective interactions among genes during GRN inference by using partial information decomposition (PID) [35] to measure multivariate information (MVI) among triplets of genes. The idea behind PID is that the information provided by two source variables X and Z about a third target variable Y can be partitioned into useful categories that help to distinguish dependence structures [34, 35]. The most relevant PID metric for GRN inference in [34] was found to be the *unique* information. The unique information, $\text{Unique}_Z(X,Y)$, quantifies how much information about the target variable Y is provided only by the source variable X given another source variable Z, and is thus not symmetric with respect to a swap of X and Y [34]. The authors found that the ratio of the unique information between X and Y relative to a third variable Z and the mutual information between X and Y

is highest when a direct link exists between X and Y [34]. Using this observation, they propose a strategy in which this ratio is computed for all pairs X and Y relative to all other possible third variables Z which they called the proportional unique contribution (PUC):

$$u_{X,Y} = \sum_{Z \in S \setminus \{X,Y\}} \frac{\text{Unique}_Z(X,Y)}{I(X,Y)} + \frac{\text{Unique}_Z(Y,X)}{I(X,Y)} \quad (5)$$

Last, they compute the confidence of an edge between X and Y using the sum of the cumulative distributions of all PUC scores involving X and Y, which accounts for variations in the distributions of PUC scores among genes due to expression variability, for example [34]. In a separate study [36], the authors applied PIDC to single-cell RNA seq data collected from mouse embryonic stem cells during their progression along the neuronal lineage. They inferred GRNs for two groups of cells that each belonged to two consecutive stages of differentiation in the attempt to identify important gene-gene interactions during such transitions [36]. Since they had three developmental stages, they inferred two such transition GRNs, identifying large rearrangements of the networks during development [36]. Although the motivation to study cells in pairs of states was stated to be because this provided sufficient heterogeneity to detect dependencies [36], it is not clear how to interpret the resulting networks as they likely represent mixtures of the GRNs before, during, and after the transition between the two developmental stages in each group. Methods which explicitly take into account time series are preferable and are discussed next.

Other studies have used information theory to predict the degree and efficiency of information transduction through developmental and signaling networks. Examples include understanding the positional information encoded by four gap genes during *Drosophila* development [37], the determination that individual TNF signaling pathways can make binary decisions [38], an approach based on conditional mutual information [39], and a theoretical study of which network topologies maximize information transmission [40]. Other techniques for undirected network inference have been developed that rely on Markov random fields [41] which will be discussed shortly. Extensive reviews of network inference and other algorithms in the context of gene regulatory networks can be found in [42, 43].

3.2 Inference for undirected time-varying networks using time series data

In the case of time series data, many other network inference techniques are available. The simplest approach is to regard the observations at each point in time as independent observations and calculate network properties for each individually, using techniques described in the previous section. This results in a collection of networks which can be further analyzed to uncover changes in properties with time. This approach essentially disregards time information during the inference step. However, other approaches incorporate the additional information of time ordering in the inference process. Since causes must precede effects, many techniques infer directional (causal) relationships from time series. A second class of dynamic network inference uses time series to infer undirected edges for networks whose topologies change over time [44] (**Fig S4**). Since this clearly violates the assumption of stationarity, these methods have been designed to specifically relax that assumption. This allows for a deeper understanding of molecular interaction networks in cells over long enough periods of time that the connections change, which often occurs during progression from one cell cycle state to the next [45]. Such approaches allow for a more rigorous understanding of questions about how interaction networks must change to enable changes in cell state. In this section we will discuss some of these approaches for time-varying undirected networks.

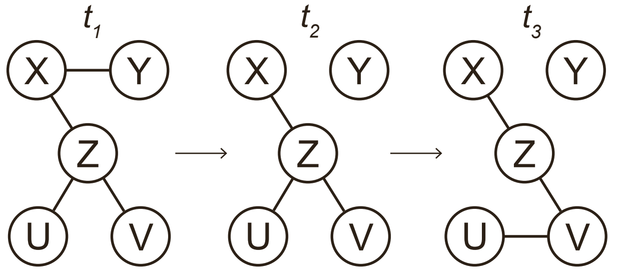

Figure S4: **Time-varying network topology.** Undirected edges between variables in a network can be added or removed as time progresses.

Sparsity. Before we review inference approaches, we first briefly discuss the phenomenon of *sparsity* upon which all of the following approaches rely. A characteristic feature of biological networks, ranging from biochemical to ecological, is that they are sparse [5, 6, 7]. Sparsity means that the real number of connections among nodes is far fewer than the maximum possible number of connections, which implies that many of the entries in the adjacency matrix, A_{ij}, are 0. An important related regression method for understanding sparse linear models is the lasso, first introduced in 1996 [46]. Given the vector of outcomes $\boldsymbol{y}$ and the covariate matrix $\mathbf{X}$, the objective of lasso is to solve

$$\min_{\alpha,\beta} \left\{ ||y - \alpha - \mathbf{X}\beta||_2^2 \right\}, \quad ||\beta||_1 < t \quad (6)$$

where $||\boldsymbol{z}||_p = \left(\sum_{i=1}^{N} |z_i|^p \right)^{1/p}$ is the L^p norm, and $t \geq 0$ is a tuning parameter [46]. The L^1 norm constraint ensures that many coefficients are equal to 0, satisfying the sparsity condition. A generalization of lasso to inference of network topology came with graphical lasso [47], which applies the L^1 constraint to the inverse covariance matrix and assumes that node values can be modeled as coming from a multivariate Gaussian distribution. The following techniques all depend on graphical lasso and extend it for certain applications.

Markov Random Fields. Another concept upon which these methods rely is the general class of undirected networks called Markov random fields [44, 48, 49]. In such networks, an undirected edge connects two nodes if they are statistically dependent after conditioning on the set of all other nodes in the network [41]. The presence of an undirected edge is then a more precise statement than the statistical dependence discussed in the previous section, but it is still degenerate with respect to the causal relationships it indicates. The degeneracy is actually greater than not knowing the direction of the underlying interaction due to collider bias (**Fig 3**). Conditioning therefore removes spurious links associated with common causes, but introduces new spurious links in the case of common effects. Since undirected edges in Markov random fields correspond to conditional dependence, the methods discussed in this section cannot account for common effects that are only addressed by causal inference methods. For a detailed review of the relationships between Markov random fields and causal models, see [41].

We now discuss several approaches which rely on the sparsity assumption and use Markov random fields to model networks whose topologies change over time. TESLA [50] is an algorithm which infers the topology of time-varying networks over a fixed set of nodes given a time series of node attributes. For networks at each time t, TESLA assumes a Markov random field model. The key component of the method is the estimation of the set of edges at all times at once using an estimator which extends graphical lasso to the case of many graphs. Importantly, their formulation assumes edges do not change much between adjacent time steps and is enforced by an additional L^1 norm constraint on the difference of the values of the same node at adjacent times. The authors applied TESLA to the voting records of senators over 12 periods of 2 months each, identifying connections among conservative Democratic senators and Republicans, for example. They also applied it to a microarray time course dataset of 4000 genes measured at 66 times during development of *D. melanogaster*, but concluded that there were too many missing genes in the dataset to draw major biological conclusions. A very similar algorithm called KELLER was introduced in the same year and also relies on a Markov random field formulation and simultaneous inference for all times to infer time-varying networks [48].

Since both TESLA and KELLER assume that networks change smoothly over time, they are not able to detect sudden or large-scale changes in network topology. A more recent technique, called Time-Varying Graphical Lasso (TVGL), addresses these issues [51]. This algorithm also uses Markov random fields to model networks at each time point. The authors consider several different possible dynamics of the network topology: only a few edges change at a time; large global changes occur; the topology changes smoothly; the topology undergoes blocky restructuring, in which a cluster of nodes changes its topology while the rest of the network is unchanged; or a single node changes all of its connections while the rest of the network remains mostly fixed. For each case, they define a unique penalty function that allows for efficient estimation [51]. For example, if the assumption is that only a few edges change at a time, the same L^1 norm constraint on node value differences from [50] is used. Other norm constraints correspond to the other dynamics, and any prior knowledge about the system can help to choose the most appropriate penalty function and thus lead to the best estimate of the dynamic topology. The authors applied their method to historical stock prices in 2010 for six large companies using the penalty which assumes that a single node can suddenly change its connections. They regarded the stock prices as an indirect readout of the interactions among the companies. They found a large deviation in network topology in the last week of January which was fully attributed to the company Apple. The authors note that on January 27th, Apple introduced the first iPad to the public, and infer connectivity changes among the companies as a result. Their approach is therefore able to detect multiple classes of network topological shifts over time. It is interesting to speculate about which penalties would be most appropriate for different biological settings, and how categories of network topological rearrangements correspond to cellular events. For example, activation of cyclin/CDK networks during cell cycle progression may correspond to blocky restructuring of GRNs [52], while cell differentiation may correspond to large, global rearrangements of GRNs [53]. We reiterate that the undirected links detected by all of these methods can correspond to either a causal interaction between the nodes, or a spurious connection which indicates that the nodes are common causes of a third node (**Fig 3**).

As a final comment, we note that all of the undirected network inference approaches reviewed so far assume that the functional forms of all interactions are pairwise [54] and are graphically represented by edges (**Fig S5A**). However, many real systems exhibit higher-order interactions that are not easily decomposed into pairwise interactions; an enzyme catalyzing an interaction between two other molecules is a simple example of a ternary interaction [55]. All of the approaches described above will necessarily be unable to detect such interactions. Work is currently being done to understand higher-order interactions [55, 56] which are relevant for molecular biology. In general, higher-order interactions among triplets, quadruplets, etc. of nodes are not represented by edges but instead by simplices. A triplet interaction can thus be represented as a triangle which is a 2-simplex, and functions describing interactions must also consider three nodes (**Fig S5B**). Inference techniques are just starting to be developed for higher-order networks and are discussed in [57]. Importantly, there are theoretical results concerning the cases in which higher-order networks reduce to pairwise-interacting networks. One study [58] shows that three-node interactions must be non-linear to cause changes to the network dynamics relative to an equivalent pairwise network.

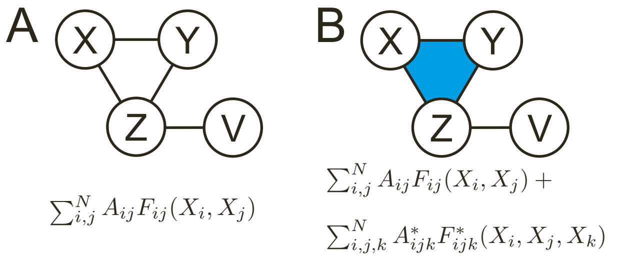

Figure S5: **Higher-order networks.** A) An undirected network whose nodes evolve according to pairwise interactions only is shown. In general, the interactions are described by the product of an adjacency matrix and functions which depend on pairs of variables. B) A higher-order network in which both triplet and pairwise interactions are relevant is shown. A corresponding additional term is required that encodes triplet interactions.

Undirected network inference can be used both for datasets at a single time point and for time series, is amenable to large numbers of variables, can model network topologies that change with time, and soon may be able to robustly infer higher-order interactions. The inference of undirected edges (simplices) between nodes offers more insight than statistical dependence, but still does not definitively establish causality for which inference of directional interactions is required.

4 Dynamical Systems

4.1 Relationships between dynamical systems approaches and causal inference

If all variables in a system are observed, every system of ODEs immediately implies a DAG and therefore a causal structure, if we assume that the variables appearing on the RHS of an ODE comprise all of the direct causes of the variable in question [15]. Of course, a single DAG can imply an infinite number of systems of ODEs, since the functional forms of interactions or even time-dependence of any sort are not specified in a DAG. Work has been done to relate systems of ODEs to the structural causal models (SCMs) introduced in the previous section [59, 60, 61]. Since SCMs do not explicitly account for time, the authors in [59] suggest an approach in which the equilibrium state of the ODEs generates the SCM. They establish a connection between do-Calculus for SCMs and resulting equilibrium states of ODEs after interventions [59]. However, this approach is limited to dynamical systems whose equilbrium states are *fixed point attractors*, to which all trajectories ultimately are drawn regardless of their initial condition. The resulting SCM then discards information about the transient behavior of trajectories on the way to equilibrium. This assumption is obviously very strong and does not encompass the behavior of many dynamical systems. Several more recent approaches have been suggested which construct SCMs from ODEs with time-varying steady states [60] and systems of stochastic differential equations (SDEs) [61]. In the case when there are unobserved variables, the terms appearing on the RHS of an ODE can only be said to be component causes, i.e. they may be either direct or indirect causes. Detection of direct versus indirect causes using dynamical systems approaches is an area of ongoing research that will be important for constructing complete, mechanistic models of cellular interaction networks that describe both causal relationships and dynamics.

4.2 Leveraging dynamical systems theory to learn DAGs

We now review some inference techniques which rely on the framework of dynamical systems as applied to networks, which we refer to as dynamical network inference. The first set of techniques assumes an underlying set of ODEs, but do not seek to explicitly find the functional forms of the interactions and instead leverage the causal assumption of RHS variables as direct causes to infer directed edges. These methods again rely on the assumption that underlying interaction networks are sparse. Interaction dynamics can be represented as a linear system $\boldsymbol{x_i} = \Phi_i \boldsymbol{a_i}$, where $\boldsymbol{x_i}$ is an m-dimensional vector of the state of node i at times $t_1, t_2, \ldots, t_m$, Φ_i is an $m \times n$ matrix encoding the functional form(s) of the interactions between node i and the other $n-1$ nodes at m times, and $\boldsymbol{a_i}$ is an n-dimensional vector which encodes the connectivity of node i with the other $n-1$ nodes. $\boldsymbol{a_i}$ can be understood as the i-th column of the adjacency matrix A_{ij}. The sparsity assumption implies that most of the elements of $\boldsymbol{a_i}$ are 0. According to the compressed sensing theory [62], such linear systems $\boldsymbol{x_i} = \Phi \boldsymbol{a_i}$ can be solved for $\boldsymbol{a_i}$ more effectively by minimizing the L^1 norm rather than the more familiar L^2 norm [62, 63]. The authors in [64] combine the L^1, which ensures sparsity, with an L^2 norm error control term to account for noise in the time series and the possibility of unobserved variables, which is related to the lasso algorithm introduced previously. Specifically, they solve:

$$\min_{\boldsymbol{a_i}} \left\{ \frac{1}{2M} ||\boldsymbol{x_i} - \Phi_i \boldsymbol{a_i}||_2^2 + \lambda ||\boldsymbol{a_i}||_1 \right\} \tag{7}$$

This method performs well on simulated data, but a major drawback is that some knowledge of the functional form of the interactions is required which is a common requirement for dynamic network inference techniques [65, 66].

As a response, "model-free" techniques have been developed which aim to infer network connectivity from time series without any prior knowledge about the underlying dynamics. In [67], the authors employ sets of basis functions to infer connectivity, without requiring an accurate reconstruction of the observed dynamics by the basis functions. The underlying framework considers a set of first order differential equations

$$\frac{dx_i}{dt} = f_i(x_i(t)) + \eta_i(t) \tag{8}$$

where x_i is the state of node i, $f_i : R^n \to R$ is a function which encodes the interactions between node i and the other $n-1$ nodes, and η_i is noise. To isolate whether node j influences node i, the authors introduce the dependency matrices, Λ^i, to the dynamical equations:

$$\frac{dx_i}{dt} = f_i(\Lambda^i x_i(t)) + \eta_i(t) \tag{9}$$

where Λ^i_j is 1 if $\dfrac{\partial f_i}{\partial x_j} \neq 0$ and 0 otherwise. By further expanding f_i into intrinsic, pairwise, third-order, etc sets of interactions, and by expanding each of those functions into sets of basis functions, the problem formulation is complete. Then, using measured time series and derivatives computed from them, links among nodes can be inferred by regression [67]. If any of the coefficients of the basis functions for a given interaction or set of interactions between nodes is non-zero, then a link is inferred. Of course, this procedure depends on the choice of basis functions, but the authors provide recommendations and evidence that a sufficient set of basis functions can correctly infer connectivity without exactly re-creating dynamics [67].

A final example approaches the problem of distinguishing direct versus indirect interactions using a dynamical systems framework. In [68], the authors infer directed networks under the assumption that interactions can be described as ODEs in which the functional relationships between variables are monotonic. They construct criteria which allow for detection of positive versus negative regulation among variables, and they apply these criteria to surrogate time series in which the time ordering is shuffled to disentangle direct versus indirect interactions [68]. This approach thus incorporates some of the benefits of causal inference, but at the cost of the assumption of monotonic functional forms that describe interactions.

4.3 Automated discovery of differential equations

Alternative approaches automate the discovery of governing equations from time series data. A set of methods called Sparse Identification of Nonlinear Dynamics (SINDy) leverages sparsity and symbolic regression to learn governing equations composed of a given set of basis functions [7, 69]. Similar to the phase portrait approach, derivatives of variables in the system are calculated from time series data and compared with the values of the variables themselves. However, SINDy automates detection of the terms in the resulting system of non-linear ODEs. The assumption is that the time-derivative of the state variables can be written as some combination of a set of basis functions:

$$\dot{X} = \Theta(X)\Xi \tag{10}$$

where $\dot{X}$ are the derivatives computed from the data, $\Theta(X)$ is a basis set of non-linear functions evaluated on the observations X, and Ξ is a vector of coefficients which specifies which basis functions contribute [69]. A reasonable assumption is that the number of terms in the governing equations is much less than the number of basis functions, so solution of the above system can be regarded as a sparse regression problem. Naturally, the authors utilize lasso, or an alternative algorithm tailored for very large datasets to solve this equation [69]. They demonstrate the efficacy of their technique by correctly inferring the equations for the Lorenz system and a mean-field model for vortex shedding using only simulated time series. They also show that SINDy can be extended to correctly infer dynamics and bifurcation diagrams for the 1D logistic map and the 2D Hopf normal form. However, the algorithm fails in the presence of too much noise in the time series measurements. As a response to this issue, SINDy has recently been extended to simultaneously discover governing equations and model noise distributions from time series [70]. The model formulation is altered to reflect that the time series measurements have contributions from both an underlying dynamics and noise, $y(t) = x(t) + n(t)$, where $y(t)$ is the measured time series, $x(t)$ is a theoretical noise-free measurement, and $n(t)$ is noise. Then, the regression problem becomes solution of the system:

$$\dot{Y} = \Theta(X + N)\Xi \tag{11}$$

The solution of such a system is more complex and relies on automatic differentiation [70]. The modified SINDy algorithm is thus able to learn governing equations and noise distributions when the noise is additive. The authors show that this modified SINDy is two times as robust to noise as the original formulation. However, the present formulation cannot account for multiplicative noise which is a common feature of many real-world processes, particularly in biology [71]. In general, optimal selection of basis functions for the SINDy algorithms is an unsolved problem, but inclusion of the proper terms is a pre-requisite for their success [70].

Inference of ODEs that describe relationships among variables allows for mathematically precise descriptions and predictions beyond what is offered by DAGs or SCMs. However, the relationships between the inferred ODEs and underlying causal structures are not clear and will require more research to fully understand. In general, the dynamical systems approach learns effective interactions between variables chosen by the researcher and does not explicitly account for unobserved variables or direct versus indirect causal interactions.

References

[1] Peter Spirtes, Clark Glymour, and Richard Scheines. *Causation, Prediction, and Search.* 2001. DOI: 10.7551/mitpress/1754.001.0001. URL: https://doi.org/10.7551/mitpress/1754.001.0001.

[2] Miguel A. Hernán and James M. Robins. "Instruments for Causal Inference: An Epidemiologist's Dream?" In: *Epidemiology* 17.4 (2006). ISSN: 1044-3983. URL: https://journals.lww.com/epidem/fulltext/2006/07000/instruments_for_causal_inference__an.4.aspx.

[3] Chun Li and Xiaodan Fan. "On nonparametric conditional independence tests for continuous variables". In: *WIREs Computational Statistics* 12.3 (2020), e1489. DOI: https://doi.org/10.1002/wics.1489. eprint: https://wires.onlinelibrary.wiley.com/doi/pdf/10.1002/wics.1489. URL: https://wires.onlinelibrary.wiley.com/doi/abs/10.1002/wics.1489.

[4] Kun Zhang et al. "Kernel-based conditional independence test and application in causal discovery". In: *Proceedings of the Twenty-Seventh Conference on Uncertainty in Artificial Intelligence.* UAI'11. Barcelona, Spain: AUAI Press, 2011, 804–813. ISBN: 9780974903972.

[5] Daniel M. Busiello et al. "Explorability and the origin of network sparsity in living systems". In: *Scientific Reports* 7.1 (2017), p. 12323. ISSN: 2045-2322. DOI: 10.1038/s41598-017-12521-1. URL: https://doi.org/10.1038/s41598-017-12521-1.

[6] Elias August and Antonis Papachristodoulou. "Efficient, sparse biological network determination". In: *BMC Systems Biology* 3.1 (2009), p. 25. ISSN: 1752-0509. DOI: 10.1186/1752-0509-3-25. URL: https://doi.org/10.1186/1752-0509-3-25.

[7] Niall M. Mangan et al. "Inferring Biological Networks by Sparse Identification of Nonlinear Dynamics". In: *IEEE Transactions on Molecular, Biological, and Multi-Scale Communications* 2.1 (2016), pp. 52–63. DOI: 10.1109/TMBMC.2016.2633265.

[8] Patrik Hoyer et al. "Nonlinear causal discovery with additive noise models". In: *Advances in Neural Information Processing Systems.* Ed. by D. Koller et al. Vol. 21. Curran Associates, Inc., 2008. URL: https://proceedings.neurips.cc/paper_files/paper/2008/file/f7664060cc52bc6f3d620bcedc94a4b6-Paper.pdf.

[9] Shohei Shimizu et al. "A Linear Non-Gaussian Acyclic Model for Causal Discovery". In: *Journal of Machine Learning Research* 7.72 (2006), pp. 2003–2030. URL: http://jmlr.org/papers/v7/shimizu06a.html.

[10] Judea Pearl. *Causality.* 2nd ed. Cambridge University Press, 2009.

[11] Jean Kaddour et al. *Causal Machine Learning: A Survey and Open Problems.* 2022. arXiv: 2206.15475 [cs.LG].

[12] Dominik Janzig Jonas Peters and Bernhard Schölkopf. *Elements of Causal Inference.* MIT Press, 2017.

[13] Cencan Xing et al. "Regulatory factor identification for nodal genes in zebrafish by causal inference". In: *Frontiers in Cell and Developmental Biology* 10 (2022). ISSN: 2296-634X. DOI: 10.3389/fcell.2022.1047363. URL: https://www.frontiersin.org/articles/10.3389/fcell.2022.1047363.

[14] Stefan Feuerriegel et al. "Causal machine learning for predicting treatment outcomes". In: *Nature Medicine* 30.4 (2024), pp. 958–968. ISSN: 1546-170X. DOI: 10.1038/s41591-024-02902-1. URL: https://doi.org/10.1038/s41591-024-02902-1.

[15] Alex Eric Yuan and Wenying Shou. "Data-driven causal analysis of observational biological time series". In: *eLife* 11 (2022). Ed. by Meredith C Schuman, e72518. ISSN: 2050-084X. DOI: 10.7554/eLife.72518. URL: https://doi.org/10.7554/eLife.72518.

[16] Jakob Runge et al. "Causal inference for time series". In: *Nature Reviews Earth & Environment* 4.7 (2023), pp. 487–505. ISSN: 2662-138X. DOI: 10.1038/s43017-023-00431-y. URL: https://doi.org/10.1038/s43017-023-00431-y.

[17] Jakob Runge et al. "Detecting and quantifying causal associations in large nonlinear time series datasets". In: *Science Advances* 5.11 (2019), eaau4996. DOI: 10.1126/sciadv.aau4996. eprint: https://www.science.org/doi/pdf/10.1126/sciadv.aau4996. URL: https://www.science.org/doi/abs/10.1126/sciadv.aau4996.

[18] Andreas Gerhardus and Jakob Runge. "High-recall causal discovery for autocorrelated time series with latent confounders". In: *Advances in Neural Information Processing Systems.* Ed. by H. Larochelle et al. Vol. 33. Curran Associates, Inc., 2020, pp. 12615–12625. URL: https://proceedings.neurips.cc/paper_files/paper/2020/file/94e70705efae423efda1088614128d0b-Paper.pdf.

[19] Kun Zhang et al. “Causal Discovery from Nonstationary/Heterogeneous Data: Skeleton Estimation and Orientation Determination”. In: *Proceedings of the Twenty-Sixth International Joint Conference on Artificial Intelligence, IJCAI-17*. 2017, pp. 1347–1353. DOI: 10.24963/ijcai.2017/187. URL: https://doi.org/10.24963/ijcai.2017/187.

[20] Biwei Huang et al. “Causal Discovery and Forecasting in Nonstationary Environments with State-Space Models”. In: *Proceedings of the 36th International Conference on Machine Learning*. Ed. by Kamalika Chaudhuri and Ruslan Salakhutdinov. Vol. 97. Proceedings of Machine Learning Research. PMLR, 2019, pp. 2901–2910. URL: https://proceedings.mlr.press/v97/huang19g.html.

[21] Elena Saggioro et al. “Reconstructing regime-dependent causal relationships from observational time series”. In: *Chaos: An Interdisciplinary Journal of Nonlinear Science* 30.11 (2020), p. 113115. ISSN: 1054-1500. DOI: 10.1063/5.0020538. URL: https://doi.org/10.1063/5.0020538.

[22] Thomas Schreiber. “Measuring Information Transfer”. In: *Phys. Rev. Lett.* 85 (2 2000), pp. 461–464. DOI: 10.1103/PhysRevLett.85.461. URL: https://link.aps.org/doi/10.1103/PhysRevLett.85.461.

[23] Takuya Imaizumi et al. “Assessing transfer entropy from biochemical data”. In: *Physical Review E* 105.3 (2022), p. 034403. DOI: 10.1103/PhysRevE.105.034403. URL: https://link.aps.org/doi/10.1103/PhysRevE.105.034403.

[24] Germán Gómez-Herrero et al. “Assessing Coupling Dynamics from an Ensemble of Time Series”. In: *Entropy* 17.4 (2015), pp. 1958–1970. ISSN: 1099-4300. DOI: 10.3390/e17041958. URL: https://www.mdpi.com/1099-4300/17/4/1958.

[25] Patricia Wollstadt et al. “Efficient Transfer Entropy Analysis of Non-Stationary Neural Time Series”. In: *PLOS ONE* 9.7 (July 2014), pp. 1–21. DOI: 10.1371/journal.pone.0102833. URL: https://doi.org/10.1371/journal.pone.0102833.

[26] Charles R. Nelson and Charles R. Plosser. “Trends and random walks in macroeconmic time series: Some evidence and implications”. In: *Journal of Monetary Economics* 10.2 (1982), pp. 139–162. ISSN: 0304-3932. DOI: https://doi.org/10.1016/0304-3932(82)90012-5. URL: https://www.sciencedirect.com/science/article/pii/0304393282900125.

[27] Mark W. Watson. “Univariate detrending methods with stochastic trends”. In: *Journal of Monetary Economics* 18.1 (1986), pp. 49–75. ISSN: 0304-3932. DOI: https://doi.org/10.1016/0304-3932(86)90054-1. URL: https://www.sciencedirect.com/science/article/pii/0304393286900541.

[28] Kung-Sik Chan Jonathan D. Cryer. *Time Series Analysis*. Springer New York, NY, 2010. DOI: https://doi.org/10.1007/978-0-387-75959-3.

[29] Roger T. Dean and William T. M. Dunsmuir. “Dangers and uses of cross-correlation in analyzing time series in perception, performance, movement, and neuroscience: The importance of constructing transfer function autoregressive models”. In: *Behavior Research Methods* 48.2 (2016), pp. 783–802. ISSN: 1554-3528. DOI: 10.3758/s13428-015-0611-2. URL: https://doi.org/10.3758/s13428-015-0611-2.

[30] Boris Podobnik and H. Eugene Stanley. “Detrended Cross-Correlation Analysis: A New Method for Analyzing Two Nonstationary Time Series”. In: *Phys. Rev. Lett.* 100 (8 2008), p. 084102. DOI: 10.1103/PhysRevLett.100.084102. URL: https://link.aps.org/doi/10.1103/PhysRevLett.100.084102.

[31] Ladislav Kristoufek. “Measuring correlations between non-stationary series with DCCA coefficient”. In: *Physica A: Statistical Mechanics and its Applications* 402 (2014), pp. 291–298. ISSN: 0378-4371. DOI: https://doi.org/10.1016/j.physa.2014.01.058. URL: https://www.sciencedirect.com/science/article/pii/S037843711400079X.

[32] Andre Levchenko and Ilya Nemenman. “Cellular noise and information transmission”. In: *Current Opinion in Biotechnology* 28 (2014), pp. 156–164. ISSN: 0958-1669. DOI: 10.1016/j.copbio.2014.05.002. URL: https://www.sciencedirect.com/science/article/pii/S0958166914000925.

[33] Adam A. Margolin et al. “ARACNE: An Algorithm for the Reconstruction of Gene Regulatory Networks in a Mammalian Cellular Context”. In: *BMC Bioinformatics* 7.1 (2006), S7. ISSN: 1471-2105. DOI: 10.1186/1471-2105-7-S1-S7. URL: https://doi.org/10.1186/1471-2105-7-S1-S7.

[34] Thalia E. Chan, Michael P.H. Stumpf, and Ann C. Babtie. “Gene Regulatory Network Inference from Single-Cell Data Using Multivariate Information Measures”. In: *Cell Systems* 5.3 (2017), 251–267.e3. ISSN: 2405-4712. DOI: 10.1016/j.cels.2017.08.014. URL: https://doi.org/10.1016/j.cels.2017.08.014.

[35] P.L. Williams and R.D. Beer. “Nonnegative decomposition of multivariate information”. In: *arXiv* (2010). DOI: 10.48550/arXiv.1004.2515.

[36] Patrick S. Stumpf et al. “Stem Cell Differentiation as a Non-Markov Stochastic Process”. In: *Cell Systems* 5.3 (2017), 268–282.e7. ISSN: 2405-4712. DOI: 10.1016/j.cels.2017.08.009. URL: https://doi.org/10.1016/j.cels.2017.08.009.

[37] Julien O. Dubuis et al. "Positional information, in bits". In: *Proceedings of the National Academy of Sciences* 110.41 (2013), pp. 16301–16308. DOI: 10.1073/pnas.1315642110. eprint: https://www.pnas.org/doi/pdf/10.1073/pnas.1315642110. URL: https://www.pnas.org/doi/abs/10.1073/pnas.1315642110.

[38] Raymond Cheong et al. "Information Transduction Capacity of Noisy Biochemical Signaling Networks". In: *Science* 334.6054 (2011), pp. 354–358. DOI: 10.1126/science.1204553. URL: https://doi.org/10.1126/science.1204553.

[39] Rosa Aghdam et al. "CN: a consensus algorithm for inferring gene regulatory networks using the SORDER algorithm and conditional mutual information test". In: *Mol. BioSyst.* 11 (3 2015), pp. 942–949. DOI: 10.1039/C4MB00413B. URL: http://dx.doi.org/10.1039/C4MB00413B.

[40] Aleksandra M. Walczak, Gašper Tkačik, and William Bialek. "Optimizing information flow in small genetic networks. II. Feed-forward interactions". In: *Physical Review E* 81.4 (2010), p. 041905. DOI: 10.1103/PhysRevE.81.041905. URL: https://link.aps.org/doi/10.1103/PhysRevE.81.041905.

[41] Laura F. Bringmann Oisín Ryan and Noémi K. Schuurman. "The Challenge of Generating Causal Hypotheses Using Network Models". In: *Structural Equation Modeling: A Multidisciplinary Journal* 29.6 (2022), pp. 953–970. DOI: 10.1080/10705511.2022.2056039. eprint: https://doi.org/10.1080/10705511.2022.2056039. URL: https://doi.org/10.1080/10705511.2022.2056039.

[42] Vân Anh Huynh-Thu and Guido Sanguinetti. "Gene Regulatory Network Inference: An Introductory Survey". In: *Gene Regulatory Networks: Methods and Protocols*. Ed. by Guido Sanguinetti and Vân Anh Huynh-Thu. New York, NY: Springer New York, 2019, pp. 1–23. ISBN: 978-1-4939-8882-2. DOI: 10.1007/978-1-4939-8882-2_1. URL: https://doi.org/10.1007/978-1-4939-8882-2_1.

[43] Michael M Saint-Antoine and Abhyudai Singh. "Network inference in systems biology: recent developments, challenges, and applications". In: *Current Opinion in Biotechnology* 63 (2020). Nanobiotechnology Systems Biology, pp. 89–98. ISSN: 0958-1669. DOI: https://doi.org/10.1016/j.copbio.2019.12.002. URL: https://www.sciencedirect.com/science/article/pii/S0958166919301399.

[44] Yongsoo Kim et al. "Inference of dynamic networks using time-course data". In: *Briefings in Bioinformatics* 15.2 (2014), pp. 212–228. ISSN: 1467-5463. DOI: 10.1093/bib/bbt028. URL: https://doi.org/10.1093/bib/bbt028.

[45] Nicholas M. Luscombe et al. "Genomic analysis of regulatory network dynamics reveals large topological changes". In: *Nature* 431.7006 (2004), pp. 308–312. ISSN: 1476-4687. DOI: 10.1038/nature02782. URL: https://doi.org/10.1038/nature02782.

[46] Robert Tibshirani. "Regression Shrinkage and Selection via the Lasso". In: *Journal of the Royal Statistical Society. Series B (Methodological)* 58.1 (1996), pp. 267–288. ISSN: 00359246. URL: http://www.jstor.org/stable/2346178 (visited on 04/30/2024).

[47] Jerome Friedman, Trevor Hastie, and Robert Tibshirani. "Sparse inverse covariance estimation with the graphical lasso". In: *Biostatistics* 9.3 (2008), pp. 432–441. ISSN: 1465-4644. DOI: 10.1093/biostatistics/kxm045. URL: https://doi.org/10.1093/biostatistics/kxm045.

[48] Le Song, Mladen Kolar, and Eric P. Xing. "KELLER: estimating time-varying interactions between genes". In: *Bioinformatics* 25.12 (2009), pp. i128–i136. ISSN: 1367-4803. DOI: 10.1093/bioinformatics/btp192. URL: https://doi.org/10.1093/bioinformatics/btp192.

[49] Michael Banf and Seung Y. Rhee. "Enhancing gene regulatory network inference through data integration with markov random fields". In: *Scientific Reports* 7.1 (2017), p. 41174. ISSN: 2045-2322. DOI: 10.1038/srep41174. URL: https://doi.org/10.1038/srep41174.

[50] Amr Ahmed and Eric P. Xing. "Recovering time-varying networks of dependencies in social and biological studies". In: *Proceedings of the National Academy of Sciences* 106.29 (2009), pp. 11878–11883. DOI: 10.1073/pnas.0901910106. eprint: https://www.pnas.org/doi/pdf/10.1073/pnas.0901910106. URL: https://www.pnas.org/doi/abs/10.1073/pnas.0901910106.

[51] David Hallac et al. "Network Inference via the Time-Varying Graphical Lasso". In: *Proceedings of the 23rd ACM SIGKDD International Conference on Knowledge Discovery and Data Mining*. KDD '17. Halifax, NS, Canada: Association for Computing Machinery, 2017, 205–213. ISBN: 9781450348874. DOI: 10.1145/3097983.3098037. URL: https://doi.org/10.1145/3097983.3098037.

[52] Robert P. Fisher. "The CDK Network: Linking Cycles of Cell Division and Gene Expression". In: *Genes & Cancer* 3.11-12 (2012). PMID: 23634260, pp. 731–738. DOI: 10.1177/1947601912473308. eprint: https://doi.org/10.1177/1947601912473308. URL: https://doi.org/10.1177/1947601912473308.

[53] Shilu Zhang et al. "Inference of cell type-specific gene regulatory networks on cell lineages from single cell omic datasets". In: *Nature Communications* 14.1 (2023), p. 3064. ISSN: 2041-1723. DOI: 10.1038/s41467-023-38637-9. URL: https://doi.org/10.1038/s41467-023-38637-9.

[54] Marc Timme and Jose Casadiego. "Revealing networks from dynamics: an introduction". In: *Journal of Physics A: Mathematical and Theoretical* 47.34 (2014), p. 343001. DOI: 10.1088/1751-8113/47/34/343001. URL: https://dx.doi.org/10.1088/1751-8113/47/34/343001.

[55] Christian Bick et al. "What Are Higher-Order Networks?" In: *SIAM Review* 65.3 (2023), pp. 686–731. DOI: 10.1137/21M1414024. eprint: https://doi.org/10.1137/21M1414024. URL: https://doi.org/10.1137/21M1414024.

[56] Federico Battiston et al. "Networks beyond pairwise interactions: Structure and dynamics". In: *Physics Reports* 874 (2020). Networks beyond pairwise interactions: Structure and dynamics, pp. 1–92. ISSN: 0370-1573. DOI: https://doi.org/10.1016/j.physrep.2020.05.004. URL: https://www.sciencedirect.com/science/article/pii/S0370157320302489.

[57] Sergio Barbarossa and Stefania Sardellitti. "Topological Signal Processing: Making Sense of Data Building on Multiway Relations". In: *IEEE Signal Processing Magazine* 37.6 (2020), pp. 174–183. DOI: 10.1109/MSP.2020.3014067.

[58] Leonie Neuhäuser, Andrew Mellor, and Renaud Lambiotte. "Multibody interactions and nonlinear consensus dynamics on networked systems". In: *Phys. Rev. E* 101 (3 2020), p. 032310. DOI: 10.1103/PhysRevE.101.032310. URL: https://link.aps.org/doi/10.1103/PhysRevE.101.032310.

[59] Joris M. Mooij, Dominik Janzing, and Bernhard Schölkopf. "From ordinary differential equations to structural causal models: the deterministic case". In: *Proceedings of the Twenty-Ninth Conference on Uncertainty in Artificial Intelligence*. UAI'13. Bellevue, WA: AUAI Press, 2013, 440–448.

[60] Paul K. Rubenstein et al. "From Deterministic ODEs to Dynamic Structural Causal Models". In: *Proceedings of the 34th Annual Conference on Uncertainty in Artificial Intelligence (UAI-18)*. 2018, pp. 114–123.

[61] Stephan Bongers, Tineke Blom, and Joris M. Mooij. *Causal Modeling of Dynamical Systems*. 2022. arXiv: 1803.08784 [cs.AI].

[62] D.L. Donoho. "Compressed sensing". In: *IEEE Transactions on Information Theory* 52.4 (2006), pp. 1289–1306. DOI: 10.1109/TIT.2006.871582.

[63] Domenico Napoletani and Timothy D. Sauer. "Reconstructing the topology of sparsely connected dynamical networks". In: *Phys. Rev. E* 77 (2 2008), p. 026103. DOI: 10.1103/PhysRevE.77.026103. URL: https://link.aps.org/doi/10.1103/PhysRevE.77.026103.

[64] Xiao Han et al. "Robust Reconstruction of Complex Networks from Sparse Data". In: *Phys. Rev. Lett.* 114 (2 2015), p. 028701. DOI: 10.1103/PhysRevLett.114.028701. URL: https://link.aps.org/doi/10.1103/PhysRevLett.114.028701.

[65] Tiago P. Peixoto. "Network Reconstruction and Community Detection from Dynamics". In: *Phys. Rev. Lett.* 123 (12 2019), p. 128301. DOI: 10.1103/PhysRevLett.123.128301. URL: https://link.aps.org/doi/10.1103/PhysRevLett.123.128301.

[66] Srinivas Gorur Shandilya and Marc Timme. "Inferring network topology from complex dynamics". In: *New Journal of Physics* 13.1 (2011), p. 013004. DOI: 10.1088/1367-2630/13/1/013004. URL: https://dx.doi.org/10.1088/1367-2630/13/1/013004.

[67] Jose Casadiego et al. "Model-free inference of direct network interactions from nonlinear collective dynamics". In: *Nature Communications* 8.1 (2017), p. 2192. ISSN: 2041-1723. DOI: 10.1038/s41467-017-02288-4. URL: https://doi.org/10.1038/s41467-017-02288-4.

[68] Se Ho Park, Seokmin Ha, and Jae Kyoung Kim. "A general model-based causal inference method overcomes the curse of synchrony and indirect effect". In: *Nature Communications* 14.1 (2023), p. 4287. ISSN: 2041-1723. DOI: 10.1038/s41467-023-39983-4. URL: https://doi.org/10.1038/s41467-023-39983-4.

[69] Steven L. Brunton, Joshua L. Proctor, and J. Nathan Kutz. "Discovering governing equations from data by sparse identification of nonlinear dynamical systems". In: *Proceedings of the National Academy of Sciences* 113.15 (2016), pp. 3932–3937. DOI: 10.1073/pnas.1517384113. eprint: https://www.pnas.org/doi/pdf/10.1073/pnas.1517384113. URL: https://www.pnas.org/doi/abs/10.1073/pnas.1517384113.

[70] Kadierdan Kaheman, Steven L Brunton, and J Nathan Kutz. "Automatic differentiation to simultaneously identify nonlinear dynamics and extract noise probability distributions from data". In: *Machine Learning: Science and Technology* 3.1 (2022), p. 015031. DOI: 10.1088/2632-2153/ac567a. URL: https://dx.doi.org/10.1088/2632-2153/ac567a.

[71] Megan A. Coomer, Lucy Ham, and Michael P.H. Stumpf. "Noise distorts the epigenetic landscape and shapes cell-fate decisions". In: *Cell Systems* 13.1 (2022), 83–102.e6. ISSN: 2405-4712. DOI: https://doi.org/10.1016/j.cels.2021.09.002. URL: https://www.sciencedirect.com/science/article/pii/S2405471221003392.
